# Integrating Gene Expression Data into Single-Step Method (ssBLUP) Improves Genomic Prediction Accuracy for Complex Traits of Duroc × Erhualian F_2_ Pig Population

**DOI:** 10.3390/cimb46120819

**Published:** 2024-12-03

**Authors:** Fangjun Xu, Zhaoxuan Che, Jiakun Qiao, Pingping Han, Na Miao, Xiangyu Dai, Yuhua Fu, Xinyun Li, Mengjin Zhu

**Affiliations:** 1 Key Lab of Agricultural Animal Genetics, Breeding, and Reproduction of Ministry of Education, Huazhong Agricultural University, Wuhan 430070, China; fjxu@webmail.hzau.edu.cn (F.X.);; 2 The Cooperative Innovation Center for Sustainable Pig Production, Huazhong Agricultural University, Wuhan 430070, China; 3 College of Animal Science and Technology, Huazhong Agricultural University, Wuhan 430070, China

**Keywords:** genomic prediction, gene expression data, best linear unbiased prediction, single-step method, pig

## Abstract

The development of multi-omics has increased the likelihood of further improving genomic prediction (GP) of complex traits. Gene expression data can directly reflect the genotype effect, and thus, they are widely used for GP. Generally, the gene expression data are integrated into multiple random effect models as independent data layers or used to replace genotype data for genomic prediction. In this study, we integrated pedigree, genotype, and gene expression data into the single-step method and investigated the effects of this integration on prediction accuracy. The integrated single-step method improved the genomic prediction accuracy of more than 90% of the 54 traits in the Duroc × Erhualian F_2_ pig population dataset. On average, the prediction accuracy of the single-step method integrating gene expression data was 20.6% and 11.8% higher than that of the pedigree-based best linear unbiased prediction (ABLUP) and genome-based best linear unbiased prediction (GBLUP) when the weighting factor (*w*) was set as 0, and it was 5.3% higher than that of the single-step best linear unbiased prediction (ssBLUP) under different *w* values. Overall, the analyses confirmed that the integration of gene expression data into a single-step method could effectively improve genomic prediction accuracy. Our findings enrich the application of multi-omics data to genomic prediction and provide a valuable reference for integrating multi-omics data into the genomic prediction model.

## 1. Introduction

The emergence of the genomic prediction (GP) method [1] as a landmark has announced the transition of animal and plant breeding from the pedigree-based era to the genome-based era [2,3]. GP is well known for increasing the prediction accuracy of estimated breeding values (EBVs) and decreasing generation interval [4]. The genomic best linear unbiased prediction (GBLUP) is a simple and commonly used implementation of GP [5,6], and GBLUP exploits genomic information, rather than pedigree information, to estimate breeding values. The GBLUP is based on the linear mixed model (LMM), which typically assumes that the phenotypic variance consists of a genetic variance component (phenotypic variation explained by genetic factors) and a residual variance component [7]. In GBLUP, the genetic similarity between individuals is represented by a genetic relationship matrix (GRM). As a widespread genetic marker in the genome, single nucleotide polymorphism (SNP) is used to represent the genotype of individuals and construct GRM [8]. With the development of sequencing technology, high-density multi-type genetic markers have been applied in practice [9,10]. The employment of high-density SNPs can improve the accuracy of genomic prediction through simulation inference [11,12]. However, this inference is not always accurate in practice [13]. In addition to SNPs, other genetic markers such as copy number variation (CNV), structural variation (SV), and insertion/deletion (indel) have also been exploited to improve genomic prediction [14,15,16,17]. All these efforts are based on genomic information. However, genomic variation fails to explain all the phenotypic variation.

Currently, the increasing research on multi-omics has aroused an extensive interest in applying multi-omics data to genomic prediction for complex traits [18]. The integration of multi-omics data, as the intermediate between genotype and phenotype, into the prediction model is expected to explain more phenotypic variation and uncover the underlying phenotype-genotype associations [19]. The transcriptomic data, namely gene expression data, can directly reflect the genotype effect, and its effectiveness has been confirmed in multiple studies [20,21]. In addition to gene-related factors, the metabolism and intestinal microorganism composition can also affect the generation process of complex traits [22,23,24,25]. The employment of metabolomics and metagenome data can improve complex trait prediction [26,27,28]. Therefore, it is necessary to include multi-omics data in genomic prediction so as to provide higher prediction accuracy. Most studies view the multi-omics data as an independent data layer or a substitute for genotype data, ignoring the association between multi-omics and genotype data. Moreover, a useful genetic evaluation framework has been proposed to integrate multi-omics data into a joint model as intermediate traits [29].

In practice, the number of individuals with gene expression data is generally much less than the number of individuals with genotype or pedigree. Inspired by the single-step best linear unbiased prediction (ssBLUP) method [30,31], in this study, we investigated the feasibility of integrating pedigree, genotype, and gene expression data into the ssBLUP. Genetic similarity between individuals is represented by a GRM integrating the three types of data. We evaluated the performance of the four prediction methods (ABLUP, GBLUP, ssGBLUP, and our integrated method), confirmed the superiority of our integrated model, and tested different weighting factors (*w*) for 54 traits of a Duroc × Erhualian F_2_ pig population dataset. In summary, this research provides a valuable reference for the inclusion of multi-omics data in genomic prediction.

## 2. Materials and Methods

### 2.1. Duroc × Erhualian F_2_ Pig Population

#### 2.1.1. Ethics Statement

Sample collection and all experimental procedures were approved by the Institutional Animal Care and Use Committee of Huazhong Agricultural University, Wuhan, China (HZAUSW-2018-008).

#### 2.1.2. Population, Phenotype, Genotype, and Gene Expression Data

The hybrid F_2_ population utilized in this study was constructed by our previous research [32], consisting of 393 F_2_ pigs derived from the crossbreeding of Duroc boars and Erhualian sows. All the pigs in the population were raised under the same indoor conditions. The 54 traits investigated in the dataset included 39 hematological traits, 5 cytokine traits, 7 immune-related traits, and 3 economic traits. A total of 294 individuals with phenotypic records were retained. The description of the 54 traits was presented in Supplementary File S1: Table S1.

Genomic DNA was extracted from ear or tail tissue samples (collected from ear notching and tail docking procedures) of piglets. All samples were genotyped by the Illumina (San Diego, CA, USA) PorcineSNP60 BeadChip, and 62,163 SNPs were obtained. Quality control was performed using PLINK 2.0 software [33]. The SNPs with a call rate <0.95 were excluded from the analysis. Subsequently, missing genotype data were imputed using Beagle 5.0 software [34]. Then, SNPs with an MAF <0.01 were excluded. Finally, a total of 39,494 SNPs were obtained for subsequent analysis.

The gene expression data used in this study have been published in previous studies [32,35]. The RNA was extracted from blood samples collected from the anterior vena cava of piglets at the age of 33 and 35 days, and RNA tagging and hybridization were performed at a commercial Affymetrix array service company (GeneTech Biotechnology Limited Company, Shanghai, China). The resulting gene expression data consisted of a total of 42 pig samples and 10,261 unique annotated genes.

### 2.2. Box–Cox Transformation for Phenotypes

Limited by the small sample size, the employment of the Box–Cox transformation method on the initial phenotypes enabled the transformed data to satisfy the assumptions of a normal linear regression model. Due to the existence of negative values in the phenotypes, we used the negative-allowed Box–Cox transformation proposed by Hawkins and Weisberg (2017) [36]. The formula is as follows:

$$y^{\left(\lambda ,\gamma \right)}=\left\{\begin{array}{c} \frac{z^{\lambda }-1}{\lambda },\;\;\lambda \neq 0\\ ln(z),\;\;\;\lambda =0\; \end{array}\right.,$$

$$z=0.5(y+\sqrt{y^{2}+\gamma^{2}})$$

where y and $y^{\left(\lambda ,\gamma \right)}$ are the initial and transformed phenotypes; λ and γ are the transformation parameters. The transformation was conducted based on the “powerTransform” function in the car R package (https://cran.r-project.org/web/packages/car/index.html, accessed on 4 September 2024).

### 2.3. Statistical Model

The commonly used best linear unbiased prediction (BLUP) was used to estimate breeding values. The solution of the model was implemented by using the Gaston R package (https://cran.r-project.org/web/packages/gaston/index.html, accessed on 26 May 2024), and the variance components in the model were estimated by the average information restricted maximum likelihood (AIREML) algorithm [37].

Six types of linear mixed models with varied additive random effects were fitted to estimated EBVs. Since the data originated from the hybrid population, a dominant random effect was also included in each model. The descriptions of these models are in the following.

Model 1 (ABLUP_D) considers the dominant effect and additive effect derived from pedigree data:

$$y=Xb+Za_{A}+Wd+e,$$

Model 2 (GBLUP_D) considers the dominant effect and additive effect derived from genotype data:

$$y=Xb+Za_{G}+Wd+e,$$

Model 3 (ssEGBLUP_D) considers the dominant effect and additive effect derived from the combination of gene expression data and genotype data:

$$y=Xb+Za_{EG}+Wd+e,$$

Model 4 (ssGABLUP_D) considers the dominant effect and additive effect derived from the combination of genotype data and pedigree data:

$$y=Xb+Za_{GA}+Wd+e,$$

Model 5 (ssEABLUP_D) considers the dominant effect and additive effect derived from the combination of gene expression data and pedigree data:

$$y=Xb+Za_{EA}+Wd+e,$$

Model 6 (ssEGABLUP_D) considers the dominant effect and additive effect derived from the combination of gene expression data, genotype data, and pedigree data:

$$y=Xb+Za_{EGA}+Wd+e,$$

where ***y*** is the vector of phenotypic values; ***b*** is the vector of fixed effects, including overall mean, sex effect, and year of birth; X is a design matrix for the fixed effects; Z and W are the design matrix for the genetic random effect; $a_{l},\;l\in \{A,E,G,EG,EA,GA,EGA\}$ are the vector of additive genetic random effect derived from varied genetic information, following the distribution of $a_{l}\sim N(0,K_{l}\sigma_{a_{l}}^{2})$, in which $K_{l}$ is the additive genetic relationship matrix, and $\sigma_{a_{l}}^{2}$ is the additive genetic variance; d is the vector of dominant genetic random effect, following the distribution of $d\sim N(0,K_{D}\sigma_{d}^{2})$, in which $K_{D}$ is the dominant genetic relationship matrix, and $\sigma_{d}^{2}$ is the dominant genetic variance; and e is an overall random residual effect, following a normal distribution with a mean of **0** and variance of $I\sigma_{e}^{2}$, in which I is the identity matrix, and $\sigma_{e}^{2}$ is the residual variance.

The $K_{A}$ was constructed based on the pedigree data and was calculated using “makeA” function in the nadiv R package (https://cran.r-project.org/web/packages/nadiv/index.html, accessed on 26 May 2024).

The $K_{E}$ was constructed based on gene expression data, which was expressed as $E=RR^{T}/r$, in which R is a standardized gene expression matrix (the average value of each column was 0) with *r* columns (*r* = the total number of genes) and *n* rows (*n* = the total number of individuals with gene expression data).

The $K_{G}$ and $K_{D}$ were constructed based on genotype data, which were expressed as [6,38]:

$$K_{G}=\frac{M_{G}{M_{G}}^{T}}{\sum_{i=1}^{m}2p_{i}(1-p_{i})},$$

$$K_{D}=\frac{M_{D}{M_{D}}^{T}}{tr(M_{D}{M_{D}}^{T})/n}$$

in which n is the total number of genotyped individuals; *m* is the total number of markers; $p_{i}$ refers to the allele frequency of A at locus i computed from all genotyped animals; $M_{G}$ is the additive marker covariates matrix as follows:

$$M_{G}=\left\{\begin{array}{c} 0-2p_{i}\\ 1-2p_{i}\\ 2-2p_{i} \end{array}\right.\;for\;genotypes\left\{\begin{array}{c} aa\\ Aa\\ AA \end{array}\right.;$$

$M_{D}$ is the dominant marker covariates matrix as follows:

$$M_{D}=\left\{\begin{array}{c} -\frac{2p_{AA}p_{Aa}}{p_{AA}+p_{aa}-{\left(p_{AA}-p_{aa}\right)}^{2}}\\ \frac{4p_{Aa}p_{Aa}}{p_{AA}+p_{aa}-{\left(p_{AA}-p_{aa}\right)}^{2}}\\ -\frac{2p_{aa}p_{Aa}}{p_{AA}+p_{aa}-{\left(p_{AA}-p_{aa}\right)}^{2}} \end{array}\right.\;for\;genotypes\left\{\begin{array}{c} aa\\ Aa\\ AA \end{array}\right..$$

where $p_{AA}$, $p_{Aa}$, and $p_{aa}$ are the genotypic frequencies for the genotypes AA, Aa and aa, respectively.

The single-step method was proposed to integrate different genetic information [30,31], and its GRM was as follows:

$$H_{KV}=function\;(K,V)=\left[\begin{array}{cc} V_{11}+V_{12}V_{22}^{-1}\;(K_{w}-V_{22})V_{22}^{-1}V_{21} & V_{12}V_{22}^{-1}K_{w}\\ K_{w}V_{22}^{-1}V_{21} & K_{w} \end{array}\right],$$

where $V_{11}$, $V_{12}$, $V_{21}$, and $V_{22}$ are the block matrices of ***V*** (the GRM based on genetic information *v*), and subscripts 1 and 2 refer to individuals without and with genetic information *k*, respectively. $V_{22}^{-1}$ is the inverse of the corresponding matrix. $K_{w}$ is defined as: $K_{w}=(1-w)K^{*}+wV_{22}$, where *w* is a weighting factor, which refers to the fraction of the genetic variation uncaptured by *k*, and $K^{*}$ is the adjusted genomic relationship matrix. Due to the differences in scale between it and the matrix $V_{22}$, $K^{*}$ matrix is expressed as: $K^{*}=\alpha K+\beta$, where α and β are derived from the following equations:

$$vg\;(diag(K))*\alpha +\beta =Avg\;(diag\;(V_{22}))\;and$$

$$AAvg\;(offdiag\;(K))*\alpha +\beta =Avg\;(offdiag\;(V_{22}))$$

in which Avg is the average value, and diag and offdiag are matrix-diagonal elements and matrix non-diagonal elements, respectively. In this study, pedigree, genotype, and gene expression data were separately integrated into a single GRM.

The $K_{EG}$ was constructed based on the integration of gene expression and genotype data and was calculated as $H_{EG}$.

The $K_{GA}$ was constructed based on the integration of genotype and pedigree data and was calculated as $H_{GA}$.

The $K_{EA}$ was constructed based on the integration of gene expression and pedigree data and was calculated as $H_{EA}$.

The $K_{EGA}$ was constructed based on the integration of gene expression, genotype, and pedigree data and was calculated as $H_{H_{EG}A}$.

### 2.4. Cross-Validation

A 10 × 10-fold cross-validation procedure was performed to evaluate the prediction accuracy. Each dataset was repeatedly split randomly into a reference dataset containing 90% of individuals and a validation dataset containing the remaining 10% of individuals for 10 times, and this dataset splitting step was further repeated 10 times.

## 3. Results

### 3.1. Integrating Gene Expression Data into ssBLUP Could Improve Prediction Accuracy of Single Traits

**Figure 1:**
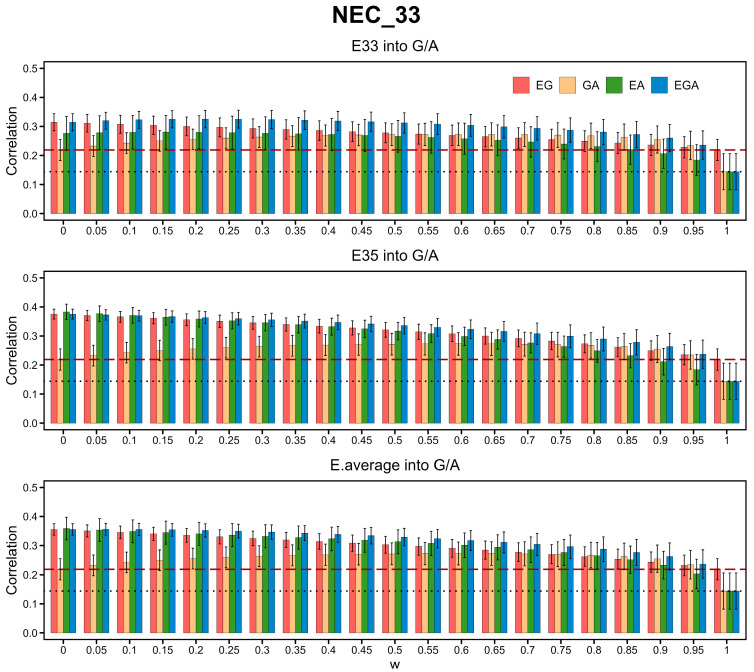
Prediction accuracy of the BLUP methods employing the genetic relationship matrix (GRM) $K_{A}$, $K_{G}$, $K_{EG}$, $K_{GA}$, $K_{EA}$, and $K_{EGA}$ for the neutrophil count trait (NEC_33) of pigs 33 days old under different *w* values. The black dotted lines and red dashed lines represent the prediction accuracy of ABLUP_D and GBLUP_D, respectively. The red, yellow, green, and blue bars represent the prediction accuracy of ssEGBLUP_D, ssGABLUP_D, ssEABLUP_D, and ssEGABLUP_D under different *w* values. E33, E35, and E.average represent gene expression data of pigs aged 33 and 35 days as well as the average gene expression value on which GRMs were constructed, respectively.

We compared the performance of BLUPs with or without gene expression information for the 54 traits of the Duroc × Erhualian F_2_ pig population dataset. All the gene expression data of pigs 33 days old (E33) and 35 days old (E35) as well as their average values (E.average) were integrated into each of $K_{A}$, $K_{G}$, and $K_{GA}.$ The prediction accuracy was evaluated based on the 10 × 10-fold cross-validation procedure, as described in the Section 2. We also tested the *w* values ranging from 0 to 1 with an interval of 0.05. For instance, the integration of gene expression data significantly improved the prediction accuracy in the trait of neutrophil count at 33 days old (NEC_33) (Figure 1). For E33, the prediction accuracy of the single-step method integrating gene expression data was 74.4%, 25.3%, and 16.6% higher than that of the ABLUP, GBLUP, and ssGBLUP. For E35, the prediction accuracy of the single-step method integrating gene expression data was 109.0%, 43.2%, and 26.3% higher than that of the ABLUP, GBLUP, and ssGBLUP. For E.average, the prediction accuracy of the single-step method integrating gene expression data was 107.0%, 36.1%, and 23.5% higher than that of the ABLUP, GBLUP, and ssGBLUP. The details of the prediction performance for the rest of the traits were displayed in Supplementary File S2: Figures S1–S53.

### 3.2. Integrating Gene Expression Data from Different Sources into ssBLUP Could Emerge Varied Prediction Performance

**Figure 2:**
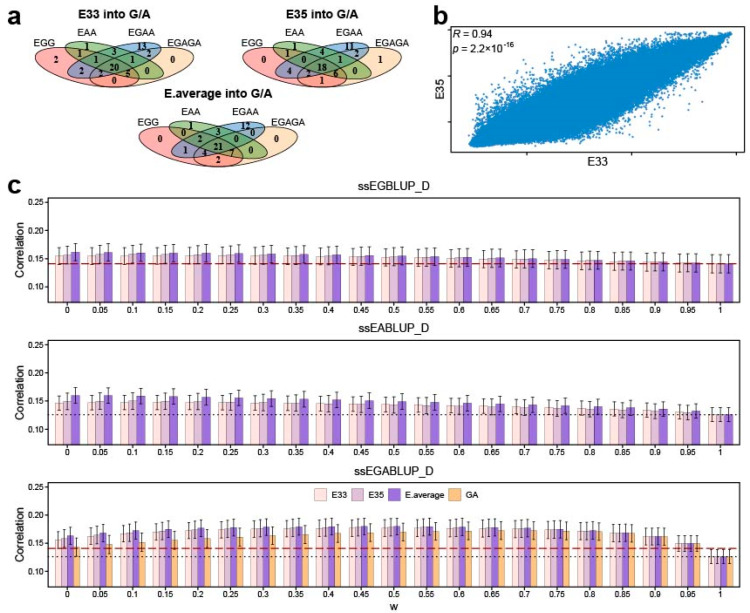
Overall prediction accuracy of methods integrating E33, E35, and E.average. (**a**) Venn diagram of the number of traits with high prediction accuracy when gene expression data were integrated into GRMs for the 54 traits of the Duroc × Erhualian F_2_ pig population. (**b**) Correlation between gene expression data of pigs aged 33 and those of pigs aged 35 days. (**c**) Average prediction accuracy of ssEGBLUP_D, ssEABLUP_D, and ssEGABLUP_D models integrating E33, E35, and E.average data for 54 traits of Duroc × Erhualian F_2_ pig population under different *w* values. The black dotted lines and red dashed lines represent the average prediction accuracy of ABLUP_D and GBLUP_D, respectively. The pale pink, light purple, purple, and orangebars indicate the average prediction accuracy of E33, E35, E.average, and ssGABLUP_D under different *w* values.

The relationship between the prediction accuracy and *w* varied with the different methods and traits. This variation was also observed between the gene expression data from different sources (Figure 1) for some traits, despite a high correlation between E33 and E35 (Figure 2b). Integration of E33, E35, and E.average improved the prediction accuracy for 53, 52, and 53 traits, respectively, and the single-step method integrating E33, E35, and E.average data outperformed all the corresponding initial BLUP methods in prediction accuracy for 20, 18, and 21 traits, respectively (Figure 2a). In general, compared with that of E33 or E35, the integration of E. average exhibited the greatest improvement in prediction accuracy (Figure 2c).

### 3.3. The Optimal w for ssBLUP Integrated Different Genetic Information

**Figure 3:**
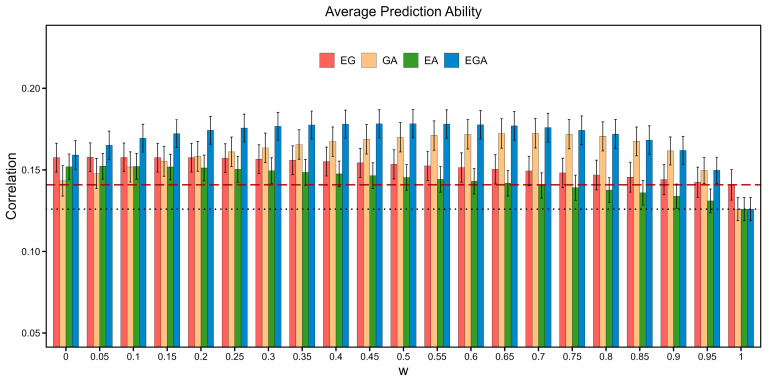
Average prediction accuracy of BLUP methods employing the genetic relationship matrix (GRM) $K_{A}$, $K_{G}$, $K_{EG}$, $K_{GA}$, $K_{EA}$, and $K_{EGA}$ for 54 traits of the Duroc × Erhualian F_2_ pig population dataset under different *w* values. The black dotted lines and red dashed lines represent the average prediction accuracy of ABLUP_D and GBLUP_D, respectively. The red, yellow, green, and blue bars denote the average prediction accuracy of ssEGBLUP_D, ssGABLUP_D, ssEABLUP_D, and ssEGABLUP_D under different *w* values.

Based on the average prediction accuracy of the BLUP methods for 54 traits, the optimal *w* value of ssBLUP using the $K_{EA}$, $K_{EG}$, $K_{GA}$, and $K_{EGA}$ was 0, 0, 0.7, and 0.55, respectively (Figure 3). The prediction accuracy of the single-step method integrating gene expression data was 20.6% and 11.8% higher than that of the ABLUP_D and GBLUP_D when the weighting factor (*w*) was set as 0, and it was 5.3% higher than that of the ssGABLUP_D under different *w* values. Overall, integrating gene expression data into the genetic evaluation model significantly improved the genomic prediction accuracy under a suitable *w* value.

## 4. Discussion

In this study, we compared the prediction accuracy of genomic prediction integrating and unintegrating gene expression data for 3 traits in the 54 traits from the Duroc × Erhualian F_2_ pig population dataset. We also explored the optimal weighting factor (*w*) of different integrative GRMs.

Theoretically, the association between phenotype and gene expression data is stronger than that between phenotype and genotype data due to the transmission of genetic information from the genome to the transcriptome. When gene expression data are used to predict phenotypic value, system error can be reduced, thus obtaining higher prediction accuracy [20,21]. However, the acquisition of gene expression data is affected by the choice of tissue [39], sampling time [40], and experimental conditions [41], which may reduce the accuracy of genomic prediction only using gene expression data [42]. In this study, we integrated pedigree, genotype, and gene expression data into the single-step method, thereby improving prediction accuracy compared with that of ABLUP, GBLUP, and ssGBLUP. As expected, the analysis results of the Duroc × Erhualian F_2_ pig population dataset indicated that for most traits, the single-step method integrating the gene expression data exhibited higher prediction accuracy than those unintegrating. For some traits, such as the traits of interferon-alpha optical density (IFNa_OD) and interferon-gamma optical density (IFNg_OD) (see Supplementary File S2 Figures S41 and S42), the prediction accuracy of the integrated method was more than twice that of the BLUP method without gene expression information.

E33 and E35 represented the gene expression data of the Duroc × Erhualian F_2_ pigs at the age of 33 days and 35 days, respectively. Although a high correlation existed between E33 and E35 and the overall prediction accuracy of the method integrating E33 was similar to that of the method integrating E35 for 54 traits, great differences in prediction accuracy between the method integrating E33 and that integrating E35 were observed for several traits, such as the traits of red cell distribution width of pigs aged 33 days (RDW_33) and white blood cell count of pigs aged 80 days (WBC_80) (see Supplementary File S2: Figures S27 and S31), indicating that the different sampling time points affected the prediction accuracy to some degree. Furthermore, we used the average value (E.average) of the two gene expression data to construct integrative GRMs for genetic evaluation, which resulted in a high average prediction accuracy for the 54 traits.

The gene expression value is also tissue-specific [39]. The differences in gene expression data from different tissues affect the accuracy of genomic prediction [43,44]. In this study, the two gene expression data (E33 and E35) of the F_2_ pig population were both obtained from a blood sample. The improvement degrees of the single-step method integrating gene expression data for cytokine traits were higher than those for other types of traits, such as economic traits (see Supplementary File S2 Figures S54–57). Therefore, using the gene expression data from the tissue related to the target trait was more appropriate.

An essential parameter of the integration of gene expression data into a single-step method was the weighting factor (*w*), which was used to ensure that the genetic relationship matrix was invertible in this study [30]. The *w* values were found to affect the prediction performance of ssBLUP [45,46]. Usually, the *w* was endowed with a small value, such as *w =* 0.01 [30]. In this study, we examined the effect of *w* values ranging from 0 to 1 with an interval of 0.05 on the prediction accuracy and found that the optimal *w* was trait- and model-specific. On average, the prediction accuracy decreased with increasing *w* values when gene expression data were integrated into ssEGBLUP and ssEABLUP. When the three types of genetic information (pedigree, genotype, and gene expression information) were integrated into a single GRM, the optimal *w* value was 0.55.

The above findings indicated that it was essential to investigate methods to reduce the uncertainty of gene expression data. The effective correction of gene expression data from different sources is expected to further improve genomic prediction accuracy. The appropriate weighting factor (*w*) could further improve the accuracy of genomic prediction with the integration of gene expression data; a low or moderate value (0.05~0.55) was recommended, resulting from our results. Regretfully, the small sample size indeed caused limitations for our analysis, which partly weakened the universality of our research. Nonetheless, our findings still provided valuable reference for integrating multi-omics data into genomic prediction. Further studies are worth implementing to validate the effectiveness of the proposed strategy conducted on a population with a large sample size.

## 5. Summary

With the development of research on multi-omics, there is an increasing need for proposing an effective strategy that integrates multi-omics data into genetic evaluation. The single-step genetic evaluation model was exploited to integrate gene expression data into genomic prediction, and this integration was confirmed to improve the accuracy of genomic prediction based on the prediction analysis of the Duroc × Erhualian F_2_ resource population. This research provides an effective reference strategy for the application of multi-omics data in genomic prediction.

## Data Availability

The genotype and gene expression data in this study are accessible on figshare (http://doi.org/10.6084/m9.figshare.26412913). The phenotype data are not publicly accessible due to their sourcing from Guangdong Wenshi Pig Breeding Technology Co., Ltd., but they can be obtained from the corresponding author upon reasonable request.

## Supplementary Materials

The following supporting information can be downloaded at: https://www.mdpi.com/article/10.3390/cimb46120819/s1, Table S1: Summary statistics of the 54 traits in F_2_ pig population; Figures S1–S53: The prediction accuracy for the rest traits; Figures S54–S57: The average prediction accuracy for immune-related, cytokine, hematological, and economic traits.

## References

[1] Meuwissen T.H., Hayes B.J., Goddard M.E. (2001). Prediction of total genetic value using genome-wide dense marker maps. Genetics.

[2] Desta Z.A., Ortiz R. (2014). Genomic selection: Genome-wide prediction in plant improvement. Trends Plant Sci..

[3] Meuwissen T., Hayes B., Goddard M. (2016). Genomic selection: A paradigm shift in animal breeding. Anim. Front..

[4] García-Ruiz A., Cole J.B., VanRaden P.M., Wiggans G.R., Ruiz-López F.J., Van Tassell C.P. (2016). Changes in genetic selection differentials and generation intervals in US Holstein dairy cattle as a result of genomic selection. Proc. Natl. Acad. Sci. USA.

[5] Henderson C.R. (1975). Best linear unbiased estimation and prediction under a selection model. Biometrics.

[6] VanRaden P.M. (2008). Efficient methods to compute genomic predictions. J. Dairy Sci..

[7] Henderson C.R. (1950). Estimation of Genetic Parameters.

[8] Marth G.T., Korf I., Yandell M.D., Yeh R.T., Gu Z., Zakeri H., Stitziel N.O., Hillier L., Kwok P.-Y., Gish W.R. (1999). A general approach to single-nucleotide polymorphism discovery. Nat. Genet..

[9] Mardis E.R. (2017). DNA sequencing technologies: 2006–2016. Nat. Protoc..

[10] Pareek C.S., Smoczynski R., Tretyn A. (2011). Sequencing technologies and genome sequencing. J. Appl. Genet..

[11] Iheshiulor O.O., Woolliams J.A., Yu X., Wellmann R., Meuwissen T.H. (2016). Within-and across-breed genomic prediction using whole-genome sequence and single nucleotide polymorphism panels. Genet. Sel. Evol..

[12] Meuwissen T., Goddard M. (2010). Accurate prediction of genetic values for complex traits by whole-genome resequencing. Genetics.

[13] Zhang C., Kemp R.A., Stothard P., Wang Z., Boddicker N., Krivushin K., Dekkers J., Plastow G. (2018). Genomic evaluation of feed efficiency component traits in Duroc pigs using 80K, 650K and whole-genome sequence variants. Genet. Sel. Evol..

[14] Chen L., Pryce J.E., Hayes B.J., Daetwyler H.D. (2021). Investigating the effect of imputed structural variants from whole-genome sequence on genome-wide association and genomic prediction in dairy cattle. Animals.

[15] Hay E.H.A., Utsunomiya Y.T., Xu L., Zhou Y., Neves H.H., Carvalheiro R., Bickhart D.M., Ma L., Garcia J.F., Liu G.E. (2018). Genomic predictions combining SNP markers and copy number variations in Nellore cattle. BMC Genom..

[16] Lyra D.H., Galli G., Alves F.C., Granato Í.S.C., Vidotti M.S., Bandeira e Sousa M., Morosini J.S., Crossa J., Fritsche-Neto R. (2019). Modeling copy number variation in the genomic prediction of maize hybrids. Theor. Appl. Genet..

[17] VanRaden P.M., Tooker M.E., O’connell J.R., Cole J.B., Bickhart D.M. (2017). Selecting sequence variants to improve genomic predictions for dairy cattle. Genet. Sel. Evol..

[18] Chung R.-H., Kang C.-Y. (2019). A multi-omics data simulator for complex disease studies and its application to evaluate multi-omics data analysis methods for disease classification. GigaScience.

[19] Vazquez A.I., Veturi Y., Behring M., Shrestha S., Kirst M., Resende M.F., de Los Campos G. (2016). Increased Proportion of Variance Explained and Prediction Accuracy of Survival of Breast Cancer Patients with Use of Whole-Genome Multiomic Profiles. Genetics.

[20] Azodi C.B., Pardo J., VanBuren R., de Los Campos G., Shiu S.H. (2020). Transcriptome-Based Prediction of Complex Traits in Maize. Plant Cell.

[21] Schrag T.A., Westhues M., Schipprack W., Seifert F., Thiemann A., Scholten S., Melchinger A.E. (2018). Beyond genomic prediction: Combining different types of omics data can improve prediction of hybrid performance in maize. Genetics.

[22] Boyle E.A., Li Y.I., Pritchard J.K. (2017). An expanded view of complex traits: From polygenic to omnigenic. Cell.

[23] Chan E.K., Rowe H.C., Hansen B.G., Kliebenstein D.J. (2010). The complex genetic architecture of the metabolome. PLoS Genet..

[24] Fan Y., Pedersen O. (2021). Gut microbiota in human metabolic health and disease. Nat. Rev. Microbiol..

[25] Vandeputte D., Falony G., Vieira-Silva S., Tito R.Y., Joossens M., Raes J. (2016). Stool consistency is strongly associated with gut microbiota richness and composition, enterotypes and bacterial growth rates. Gut.

[26] Guo Z., Magwire M.M., Basten C.J., Xu Z., Wang D. (2016). Evaluation of the utility of gene expression and metabolic information for genomic prediction in maize. Theor. Appl. Genet..

[27] Riedelsheimer C., Czedik-Eysenberg A., Grieder C., Lisec J., Technow F., Sulpice R., Altmann T., Stitt M., Willmitzer L., Melchinger A.E. (2012). Genomic and metabolic prediction of complex heterotic traits in hybrid maize. Nat. Genet..

[28] Weishaar R., Wellmann R., Camarinha-Silva A., Rodehutscord M., Bennewitz J. (2020). Selecting the hologenome to breed for an improved feed efficiency in pigs-A novel selection index. J. Anim. Breed. Genet..

[29] Christensen O.F., Borner V., Varona L., Legarra A. (2021). Genetic evaluation including intermediate omics features. Genetics.

[30] Christensen O.F., Lund M.S. (2010). Genomic prediction when some animals are not genotyped. Genet. Sel. Evol..

[31] Legarra A., Christensen O.F., Aguilar I., Misztal I. (2014). Single Step, a general approach for genomic selection. Livest. Sci..

[32] Liu X., Huang J., Yang S., Zhao Y., Xiang A., Cao J., Fan B., Wu Z., Zhao J., Zhao S. (2014). Whole blood transcriptome comparison of pigs with extreme production of in vivo dsRNA-induced serum IFN-a. Dev. Comp. Immunol..

[33] Purcell S., Neale B., Todd-Brown K., Thomas L., Ferreira M.A., Bender D., Maller J., Sklar P., de Bakker P.I., Daly M.J. (2007). PLINK: A tool set for whole-genome association and population-based linkage analyses. Am. J. Hum. Genet..

[34] Browning B.L., Zhou Y., Browning S.R. (2018). A One-Penny Imputed Genome from Next-Generation Reference Panels. Am. J. Hum. Genet..

[35] Han P., Wang C., Zhang W., Wu Y., Wang D., Zhao S., Zhu M. (2023). Pleiotropic architectures of porcine immune and growth trait pairs revealed by a self-product-based transcriptome method. Anim. Genet..

[36] Hawkins D., Weisberg S. (2017). Combining the box-cox power and generalised log transformations to accommodate nonpositive responses in linear and mixed-effects linear models. South Afr. Stat. J..

[37] Gilmour A.R., Thompson R., Cullis B.R. (1995). Average information REML: An efficient algorithm for variance parameter estimation in linear mixed models. Biometrics.

[38] Vitezica Z.G., Legarra A., Toro M.A., Varona L. (2017). Orthogonal estimates of variances for additive, dominance, and epistatic effects in populations. Genetics.

[39] Sonawane A.R., Platig J., Fagny M., Chen C.-Y., Paulson J.N., Lopes-Ramos C.M., DeMeo D.L., Quackenbush J., Glass K., Kuijjer M.L. (2017). Understanding tissue-specific gene regulation. Cell Rep..

[40] Bar-Joseph Z., Gerber G.K., Gifford D.K., Jaakkola T.S., Simon I. (2003). Continuous representations of time-series gene expression data. J. Comput. Biol..

[41] Schmittgen T.D., Zakrajsek B.A. (2000). Effect of experimental treatment on housekeeping gene expression: Validation by real-time, quantitative RT-PCR. J. Biochem. Biophys. Methods.

[42] Westhues M., Schrag T.A., Heuer C., Thaller G., Utz H.F., Schipprack W., Thiemann A., Seifert F., Ehret A., Schlereth A. (2017). Omics-based hybrid prediction in maize. Theor. Appl. Genet..

[43] Fryett J.J., Morris A.P., Cordell H.J. (2020). Investigation of prediction accuracy and the impact of sample size, ancestry, and tissue in transcriptome-wide association studies. Genet. Epidemiol..

[44] Weisweiler M., Montaigu A.d., Ries D., Pfeifer M., Stich B. (2019). Transcriptomic and presence/absence variation in the barley genome assessed from multi-tissue mRNA sequencing and their power to predict phenotypic traits. BMC Genom..

[45] Kang H., Ning C., Zhou L., Zhang S., Yan Q., Liu J.-F. (2018). Single-step genomic evaluation of milk production traits using multiple-trait random regression model in Chinese Holsteins. J. Dairy Sci..

[46] Martini J.W., Schrauf M.F., Garcia-Baccino C.A., Pimentel E.C., Munilla S., Rogberg-Muñoz A., Cantet R.J., Reimer C., Gao N., Wimmer V. (2018). The effect of the H^−1^ scaling factors τ and ω on the structure of H in the single-step procedure. Genet. Sel. Evol..

