# Supplementary material for: Integrating Gene Expression Data into Single-Step Method (ssBLUP) Improves Genomic Prediction Accuracy for Complex Traits of Duroc × Erhualian F2 Pig Population
Source: Curr Issues Mol Biol. 2024 Dec 3;46(12):13713–24. doi: 10.3390/cimb46120819 (PMC11727526; doi:10.3390/cimb46120819)
Supplement: Supplementary file 1 [file cimb-46-00819-s001.zip › Supplementary Figures S1-S57.pdf]

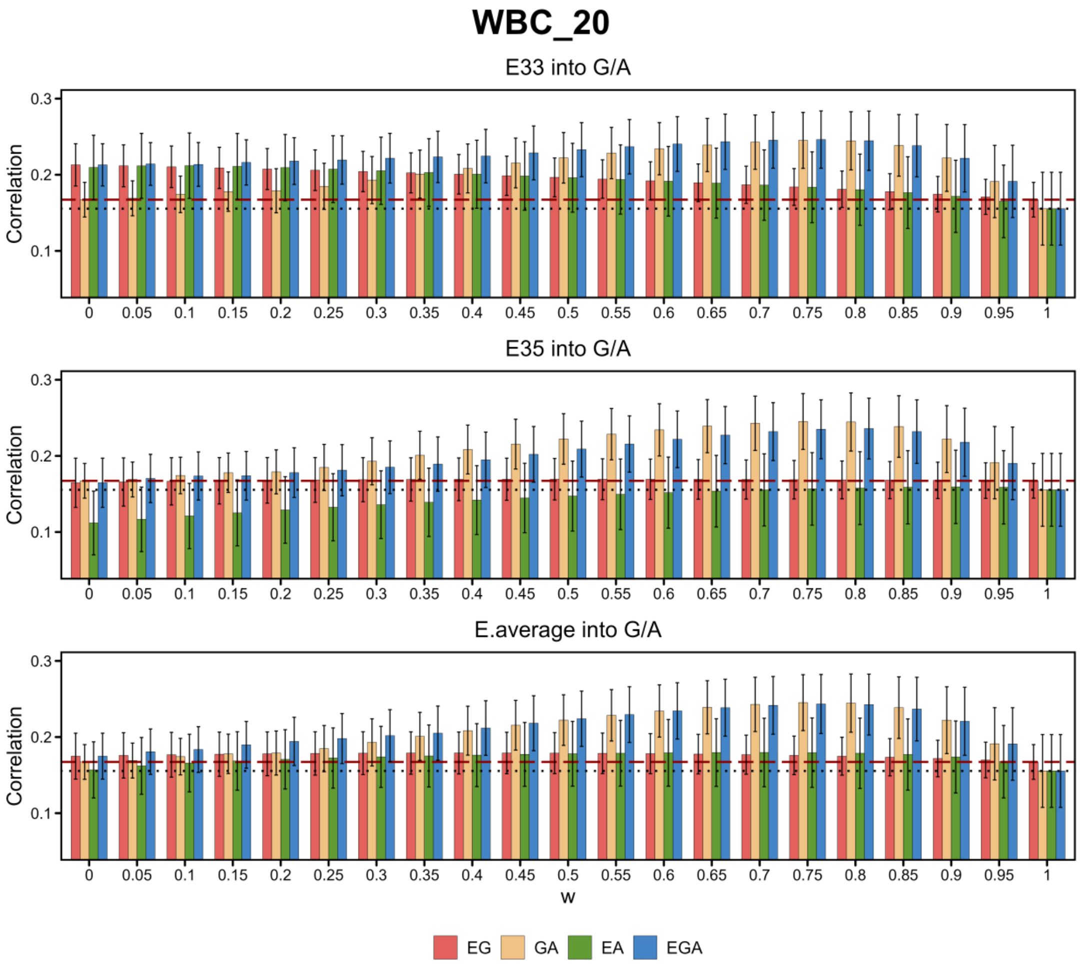

Figure S1. The Prediction Accuracy for trait of WBC_20

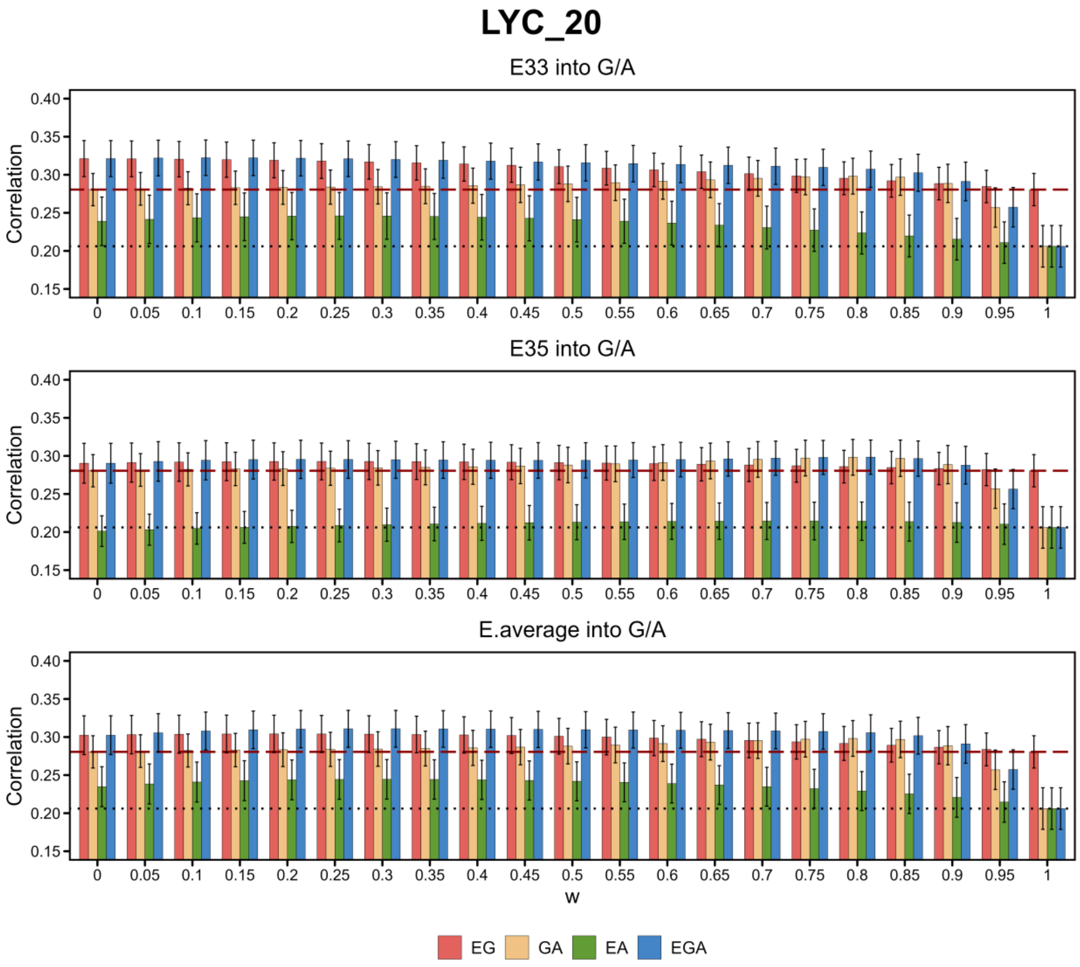

Figure S2. The Prediction Accuracy for trait of LYC_20

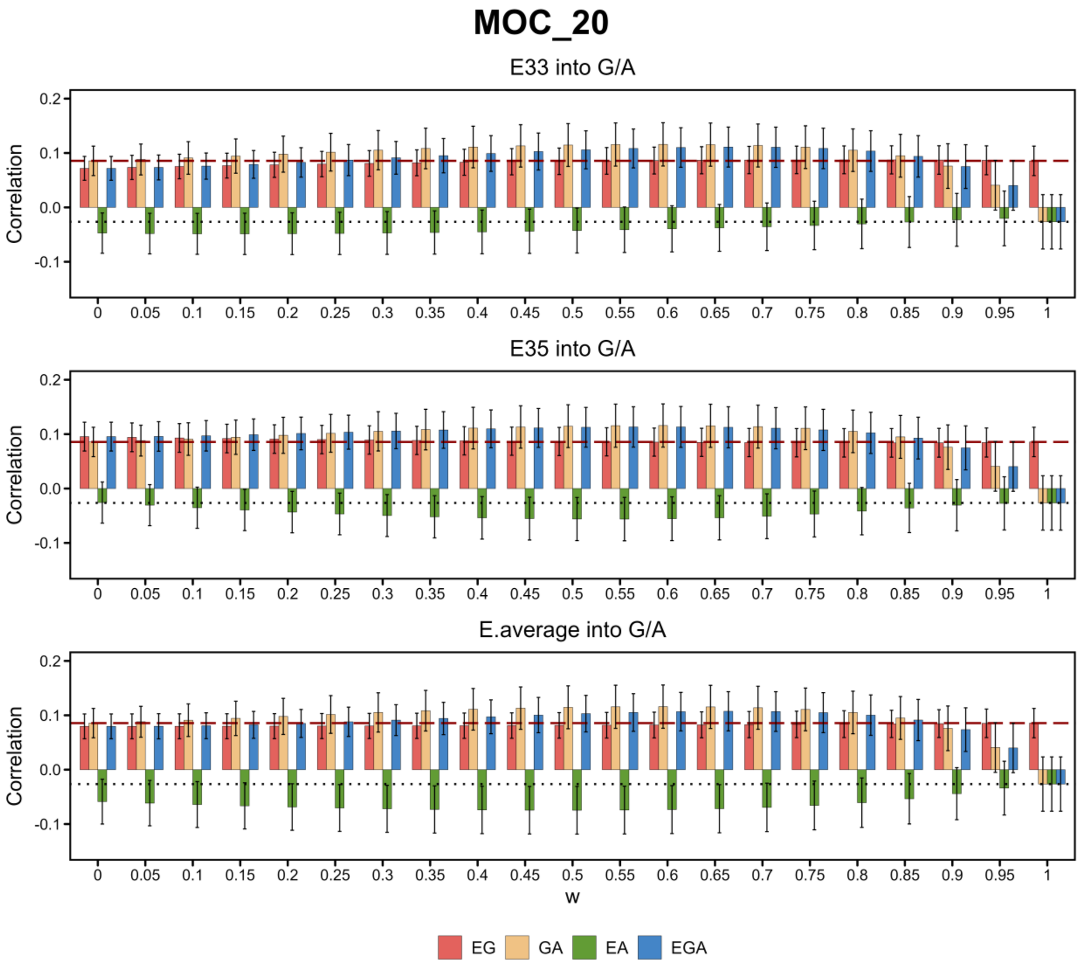

Figure S3. The Prediction Accuracy for trait of MOC_20

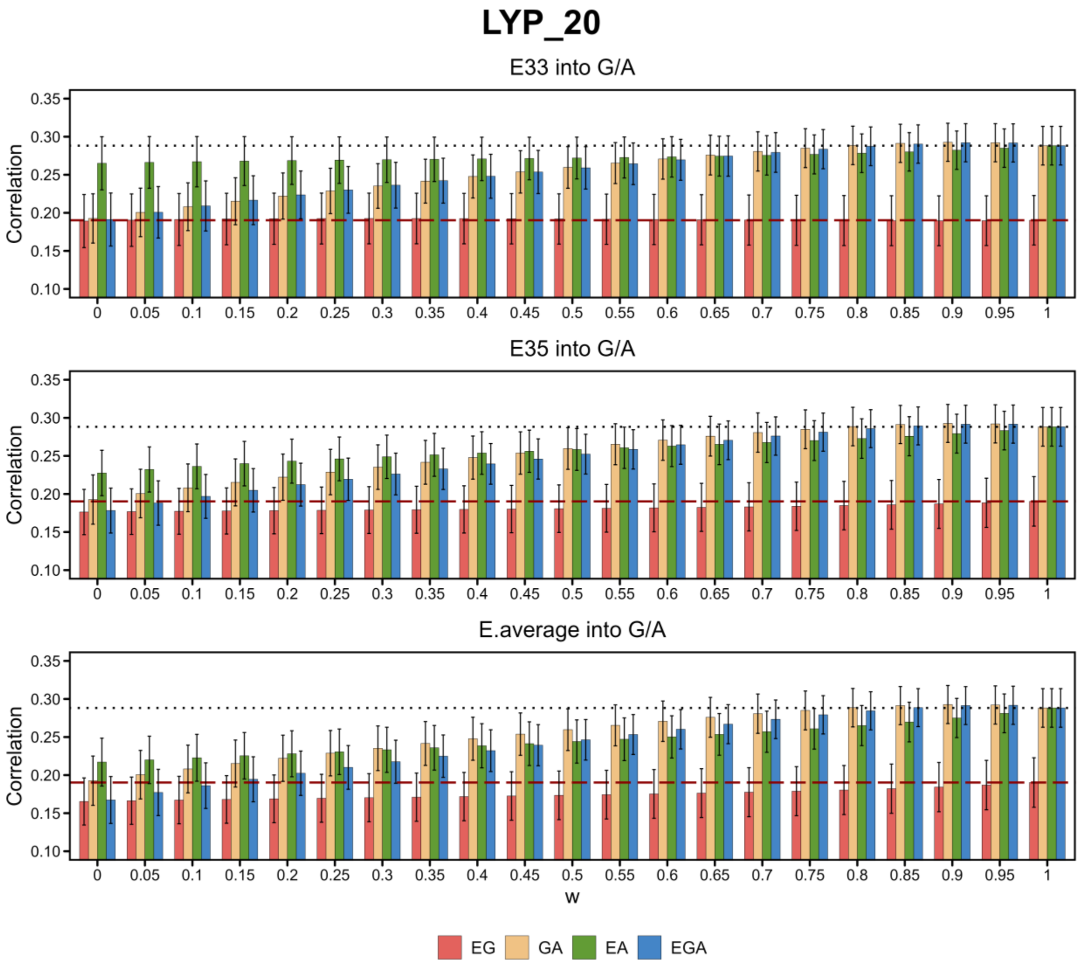

Figure S4. The Prediction Accuracy for trait of LYP_20

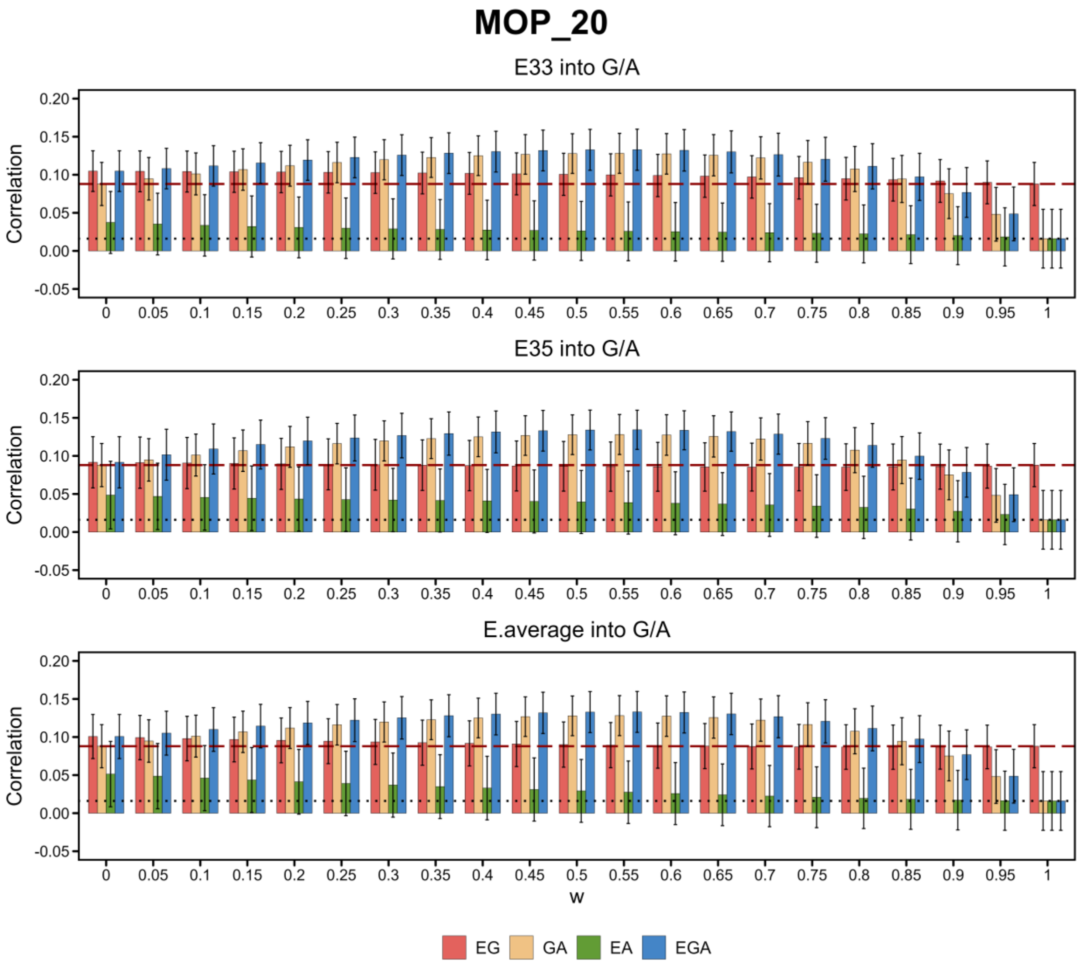

Figure S5. The Prediction Accuracy for trait of MOP_20

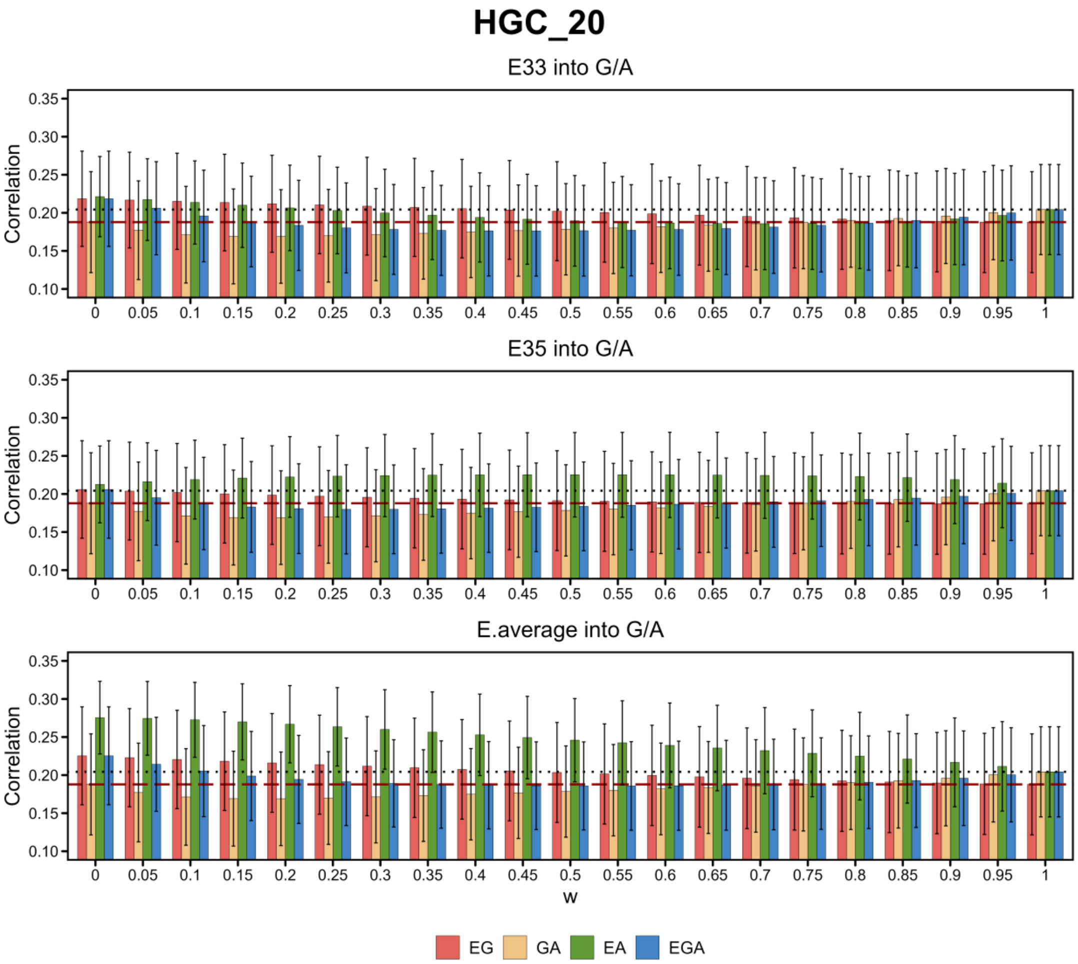

Figure S6. The Prediction Accuracy for trait of HGC_20

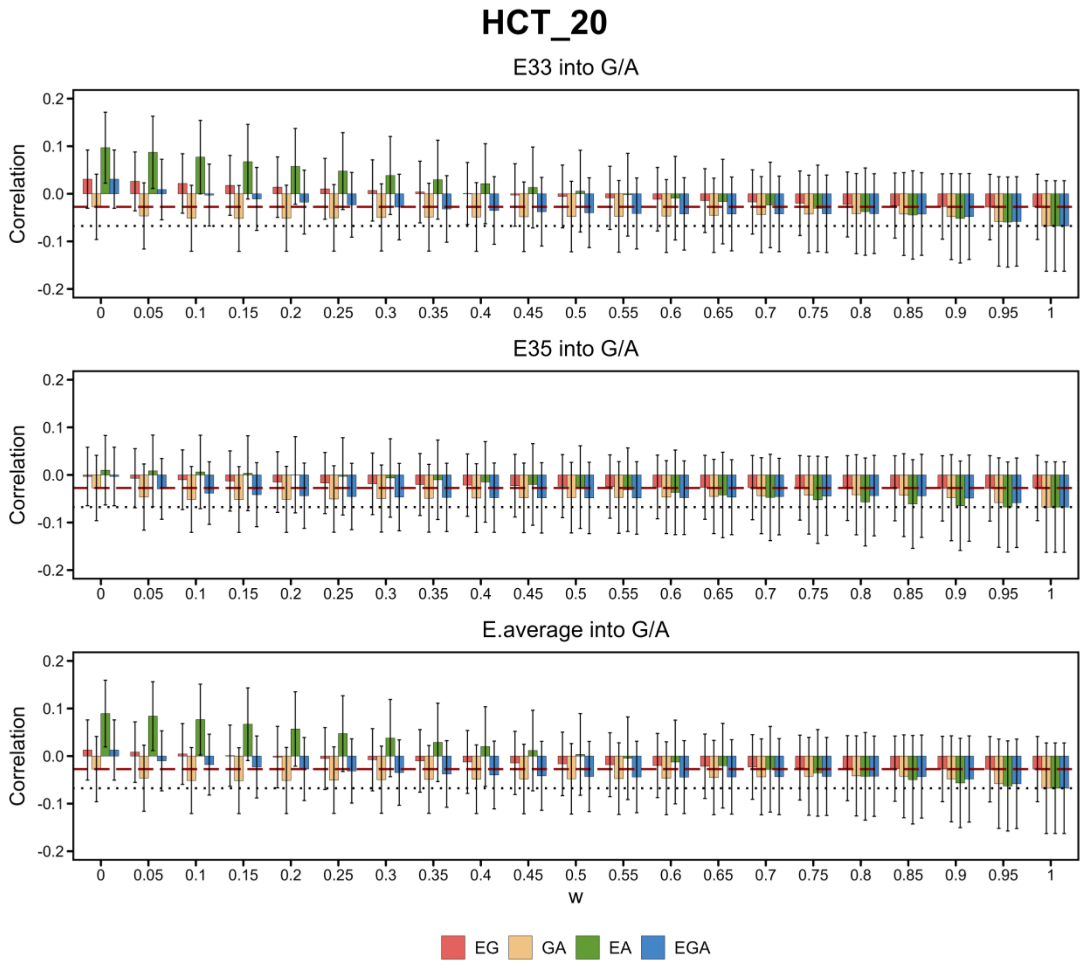

Figure S7. The Prediction Accuracy for trait of HCT_20

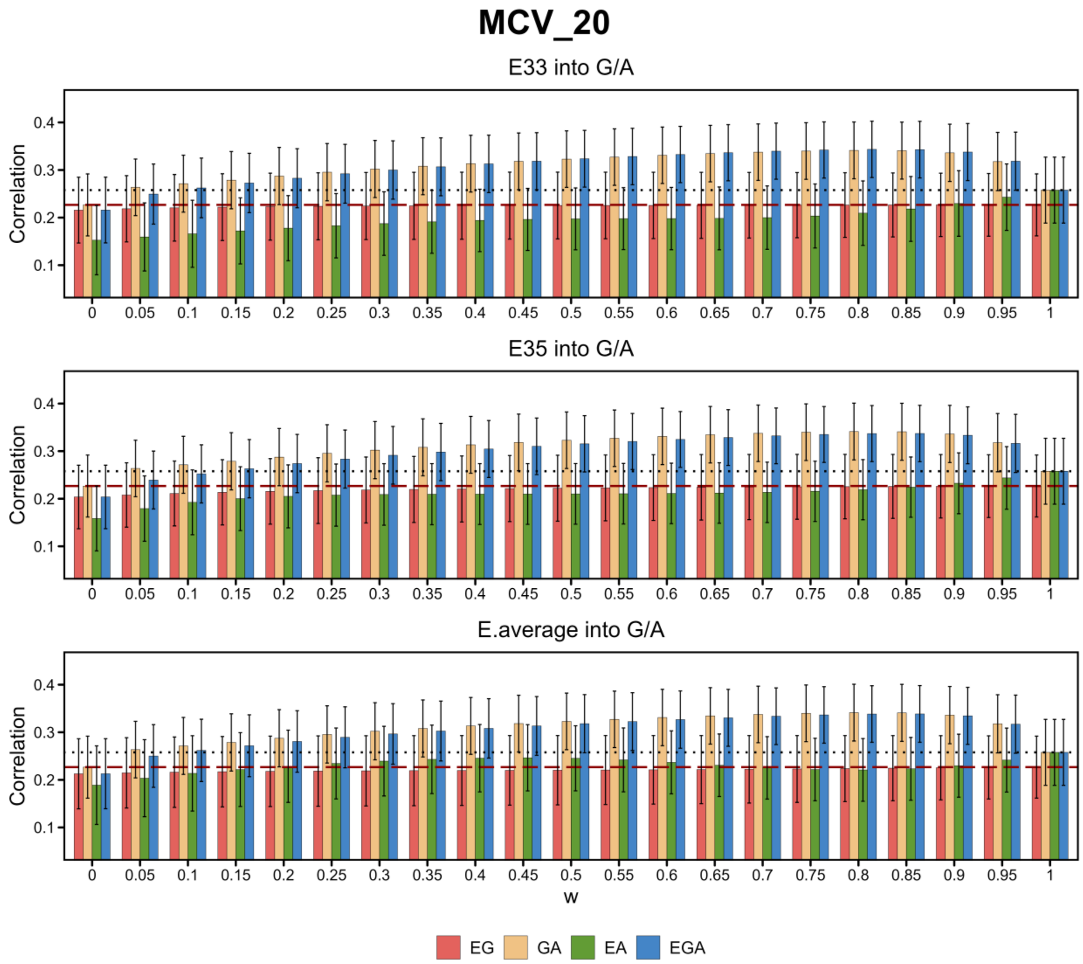

Figure S8. The Prediction Accuracy for trait of MCV_20

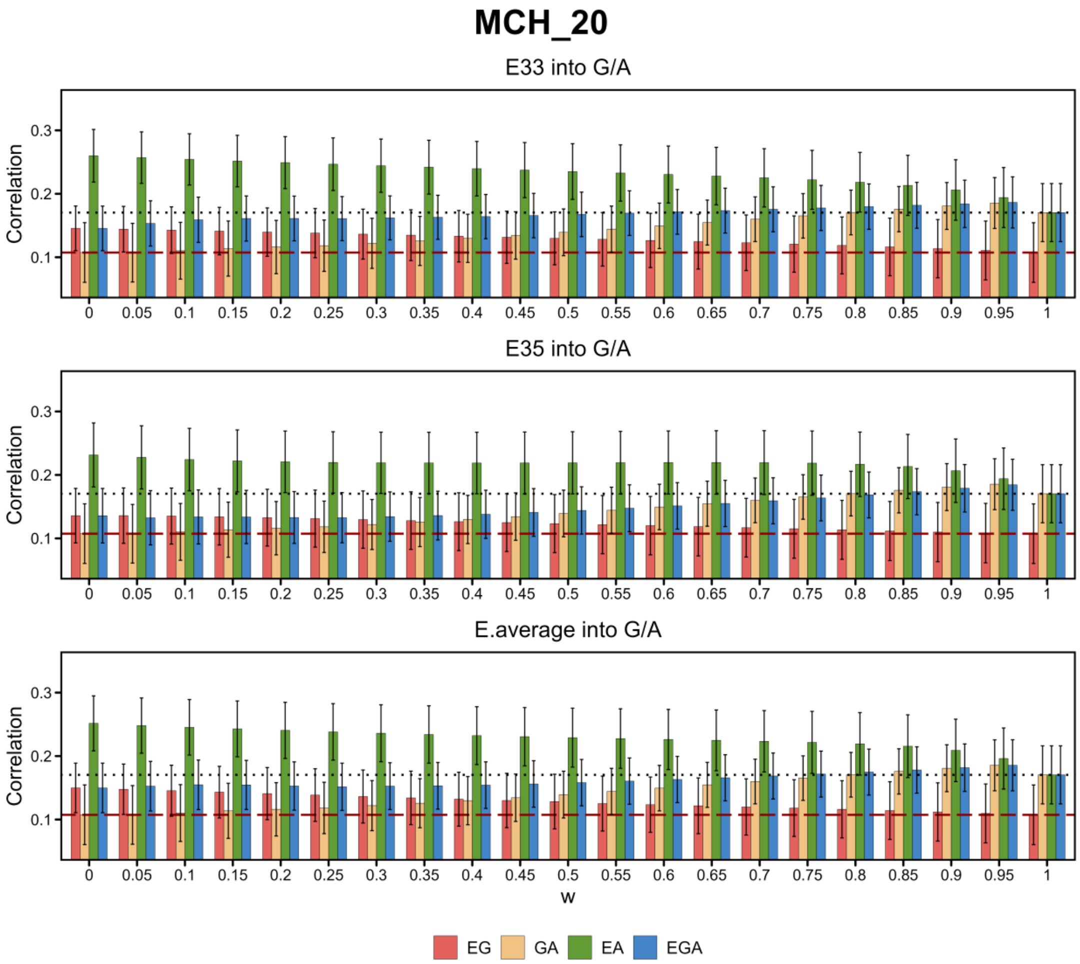

Figure S9. The Prediction Accuracy for trait of MCH_20

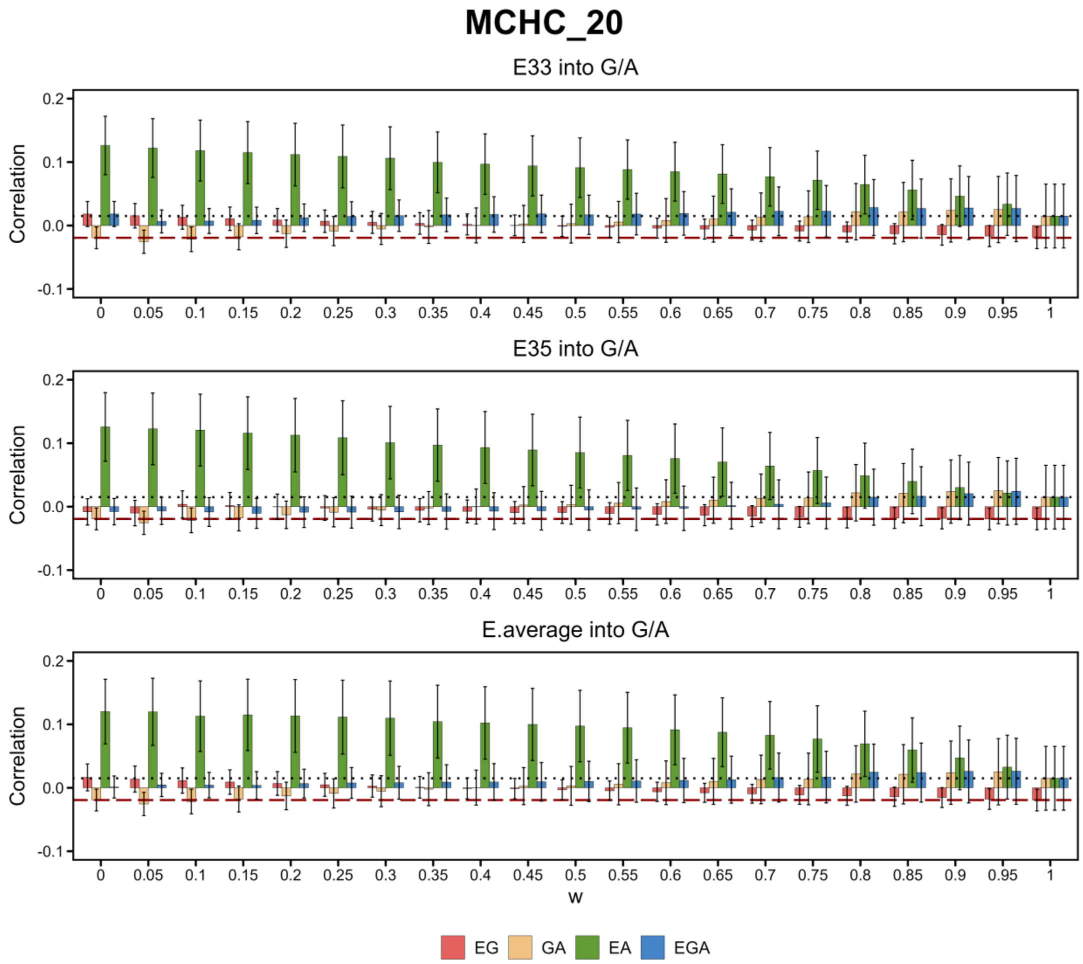

Figure S10. The Prediction Accuracy for trait of MCHC_20

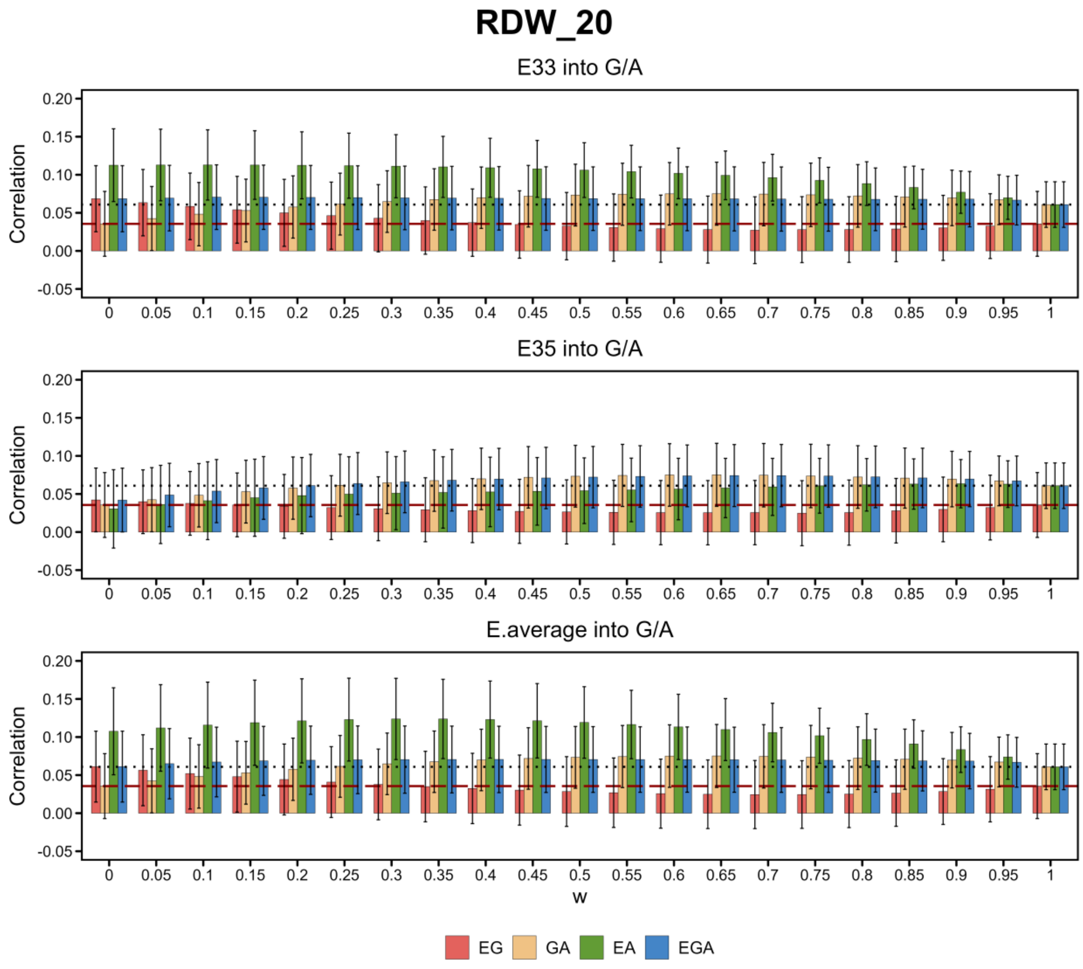

Figure S11. The Prediction Accuracy for trait of RDW_20

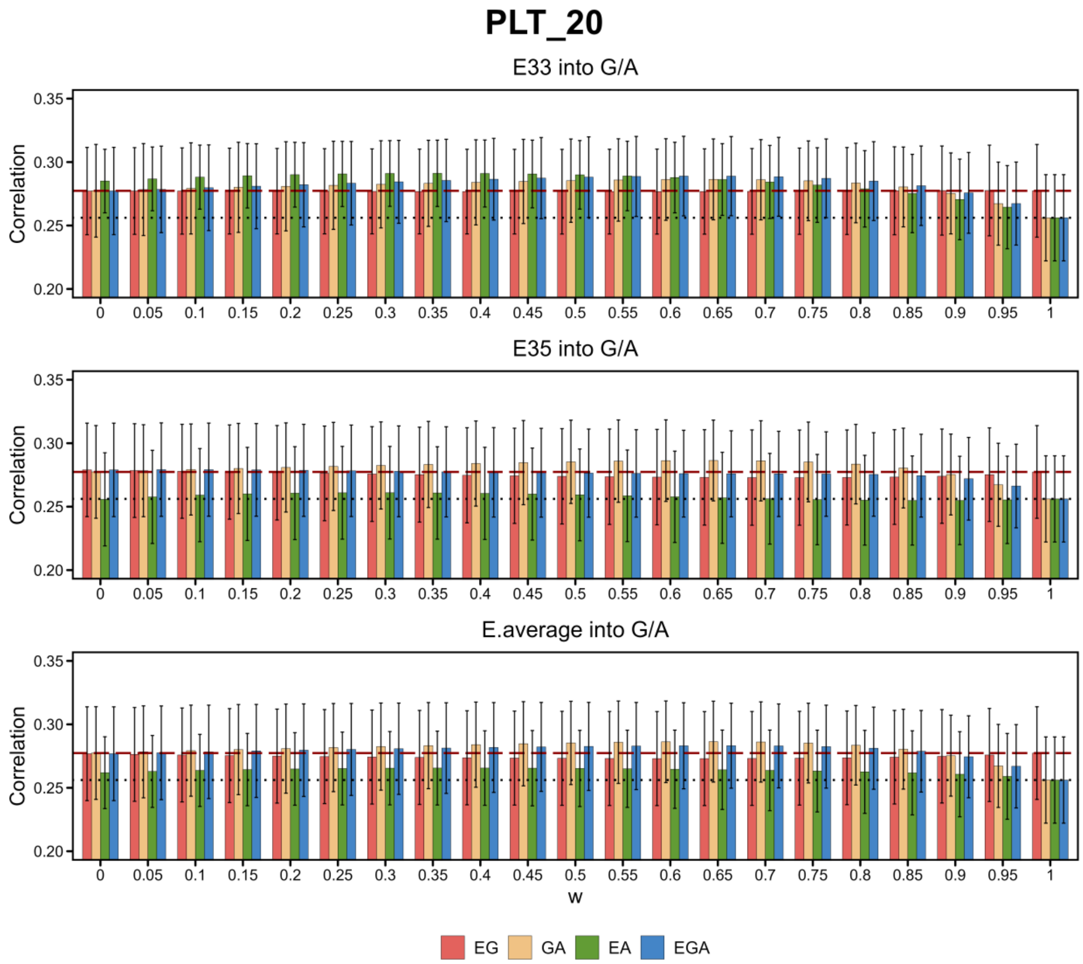

Figure S12. The Prediction Accuracy for trait of PLT_20

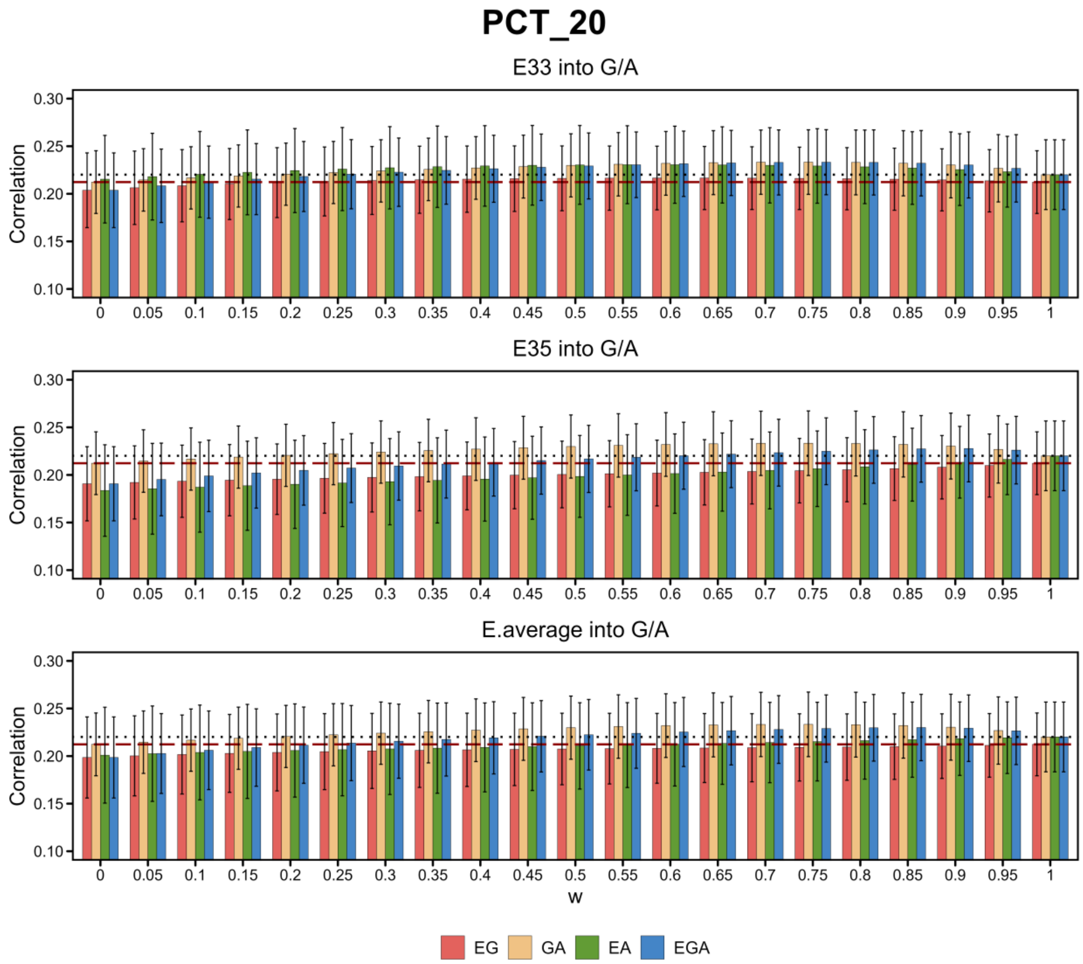

Figure S13. The Prediction Accuracy for trait of PCT_20

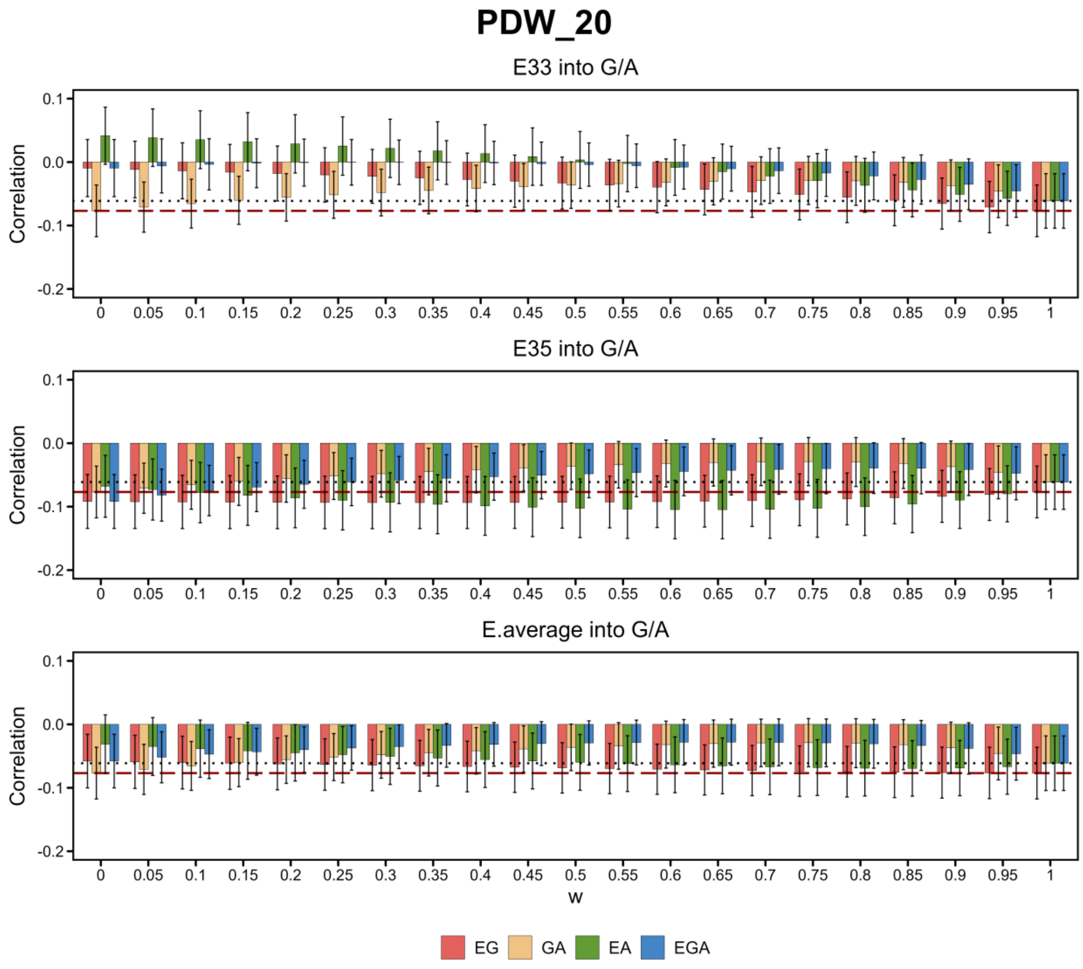

Figure S14. The Prediction Accuracy for trait of PDW_20

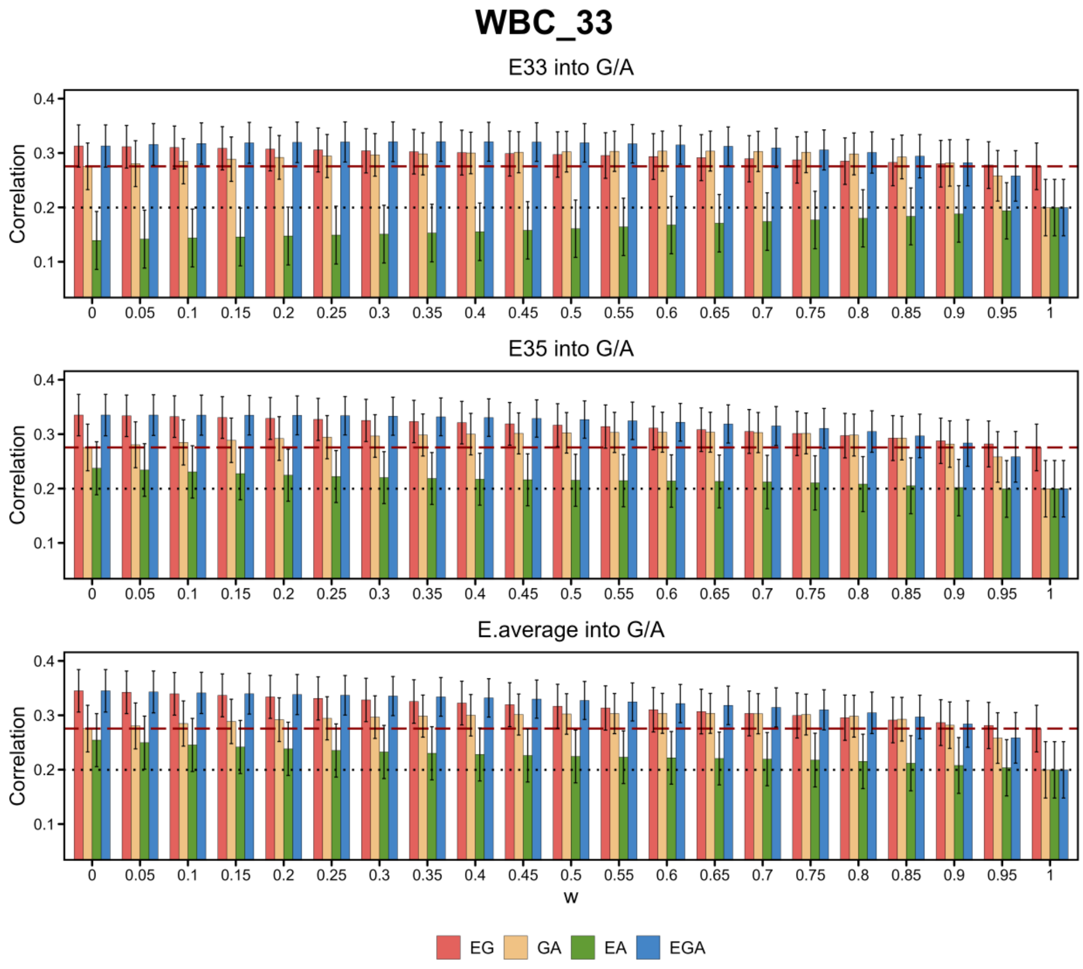

Figure S15. The Prediction Accuracy for trait of WBC_33

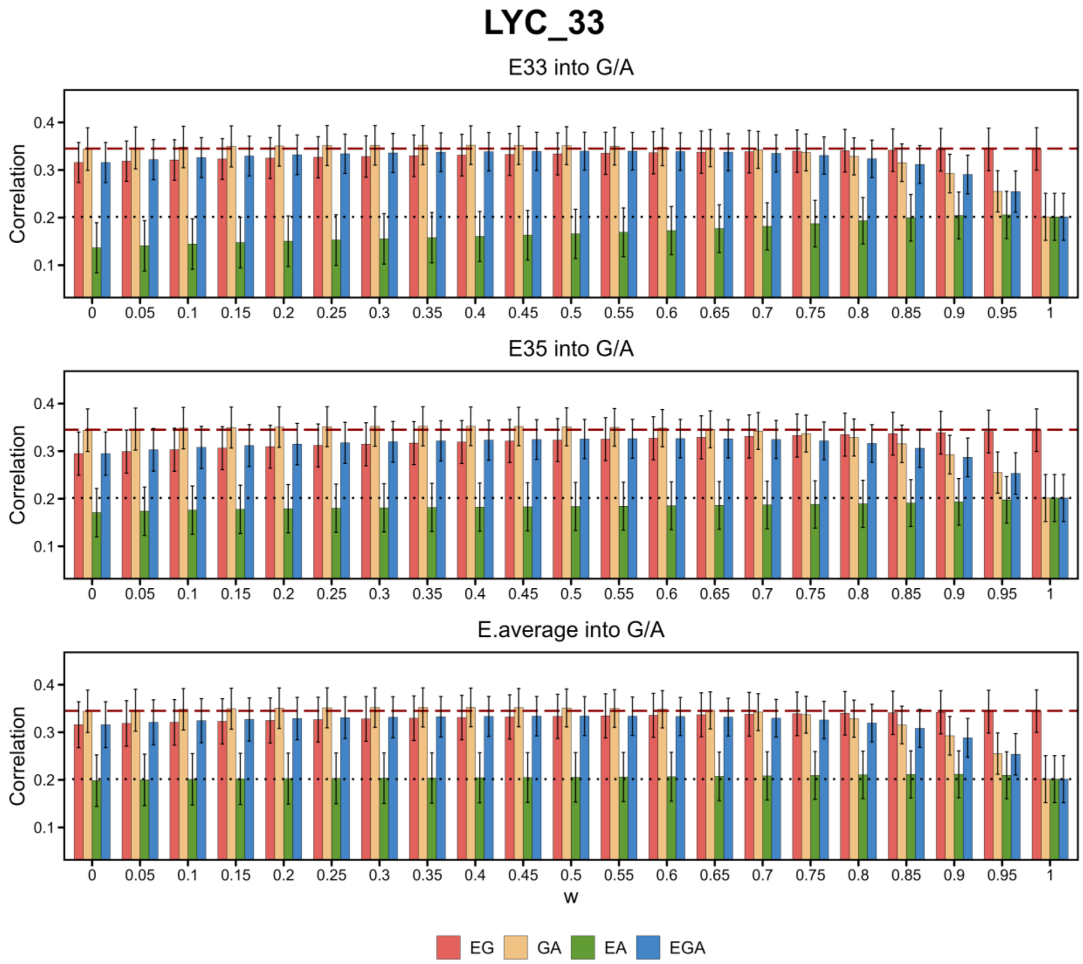

Figure S16. The Prediction Accuracy for trait of LYC_33

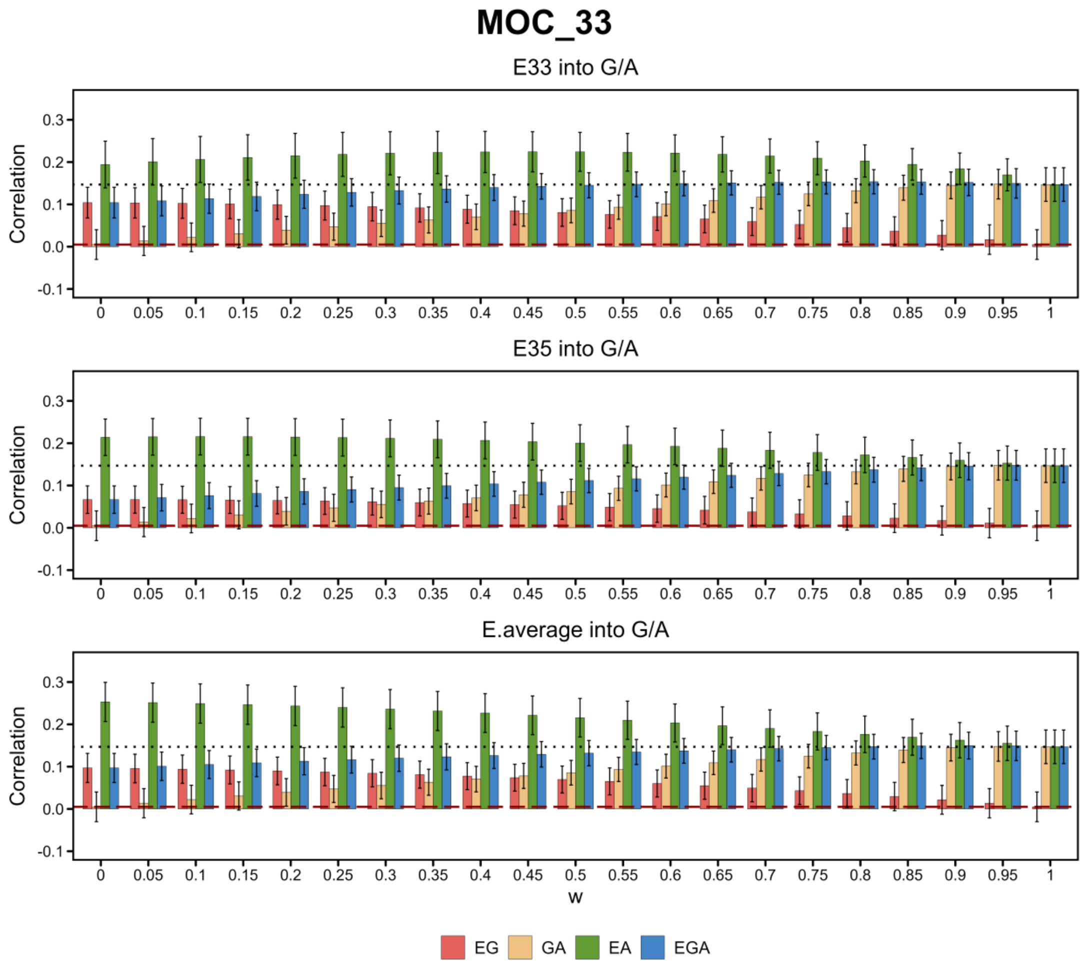

Figure S17. The Prediction Accuracy for trait of MOC_33

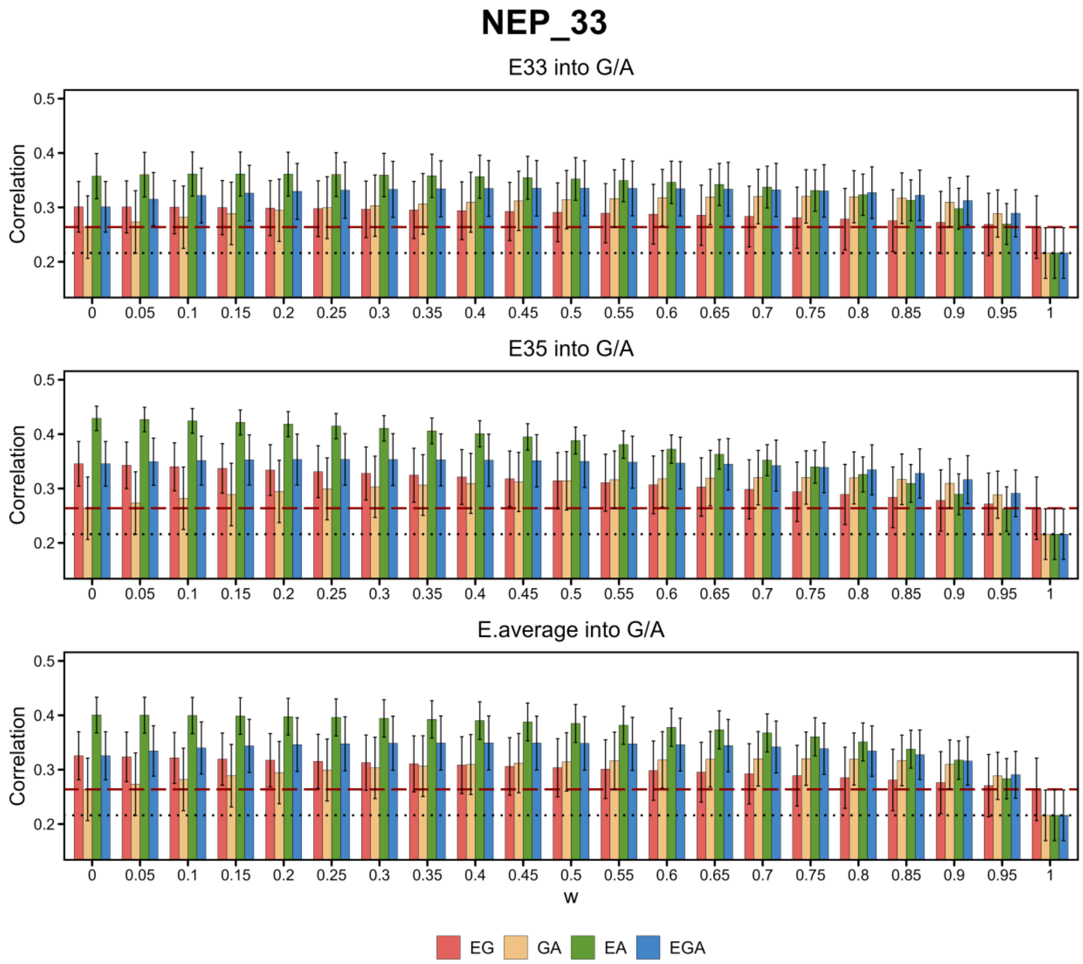

Figure S18. The Prediction Accuracy for trait of NEP_33

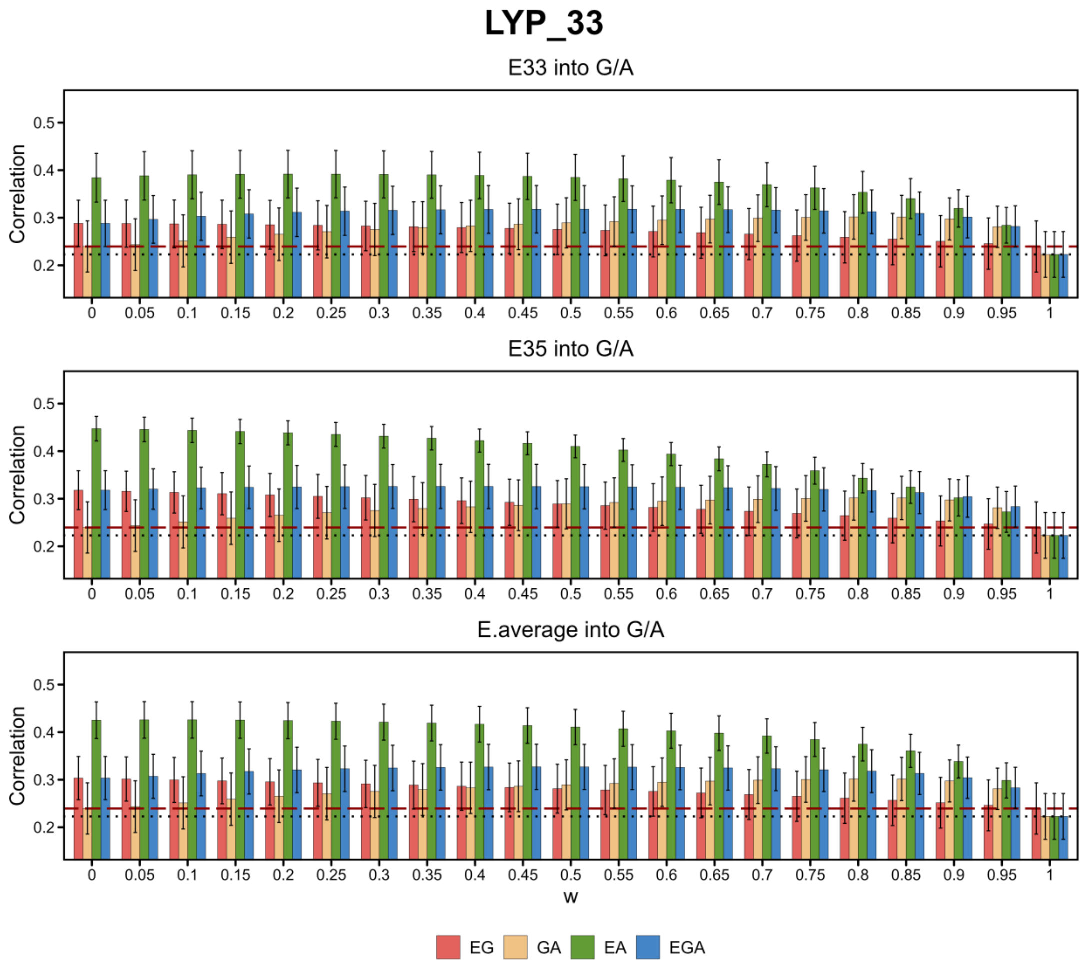

Figure S19. The Prediction Accuracy for trait of LYP_33

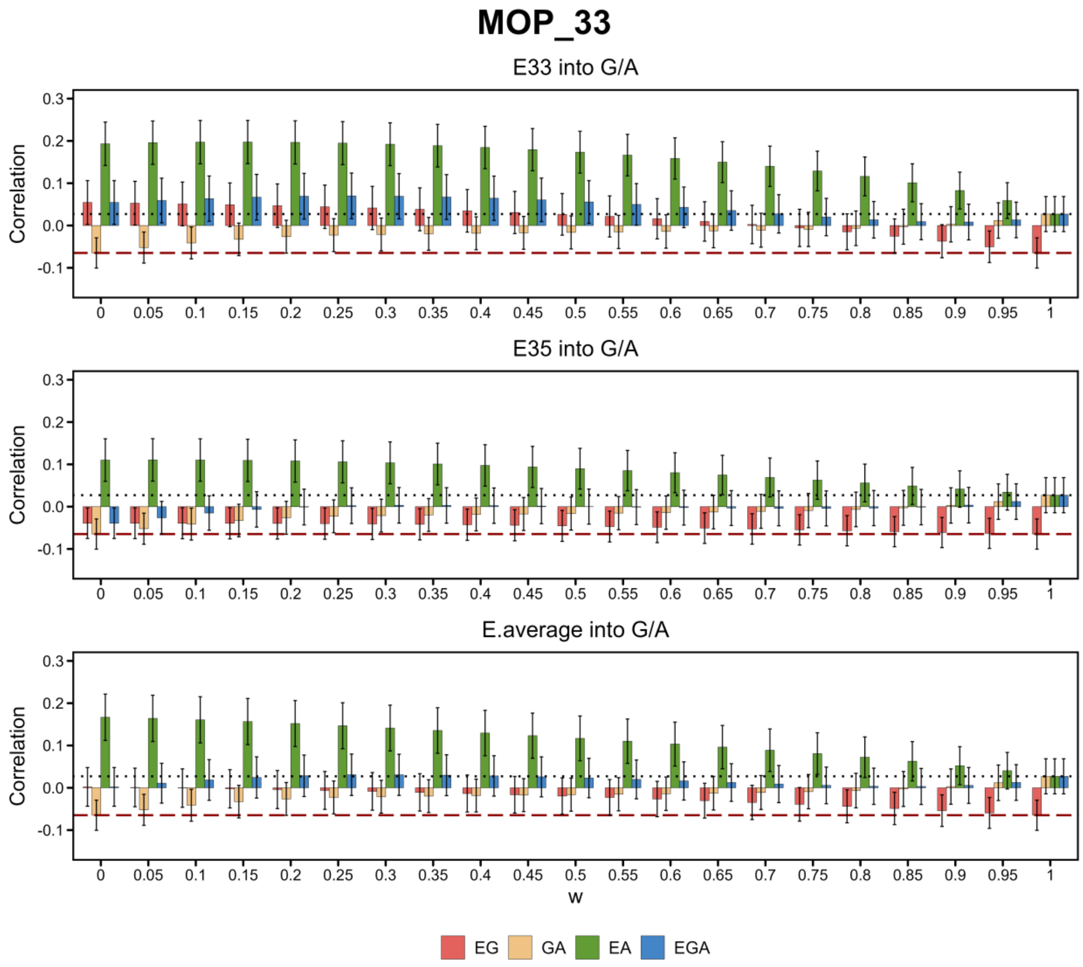

Figure S20. The Prediction Accuracy for trait of MOP_33

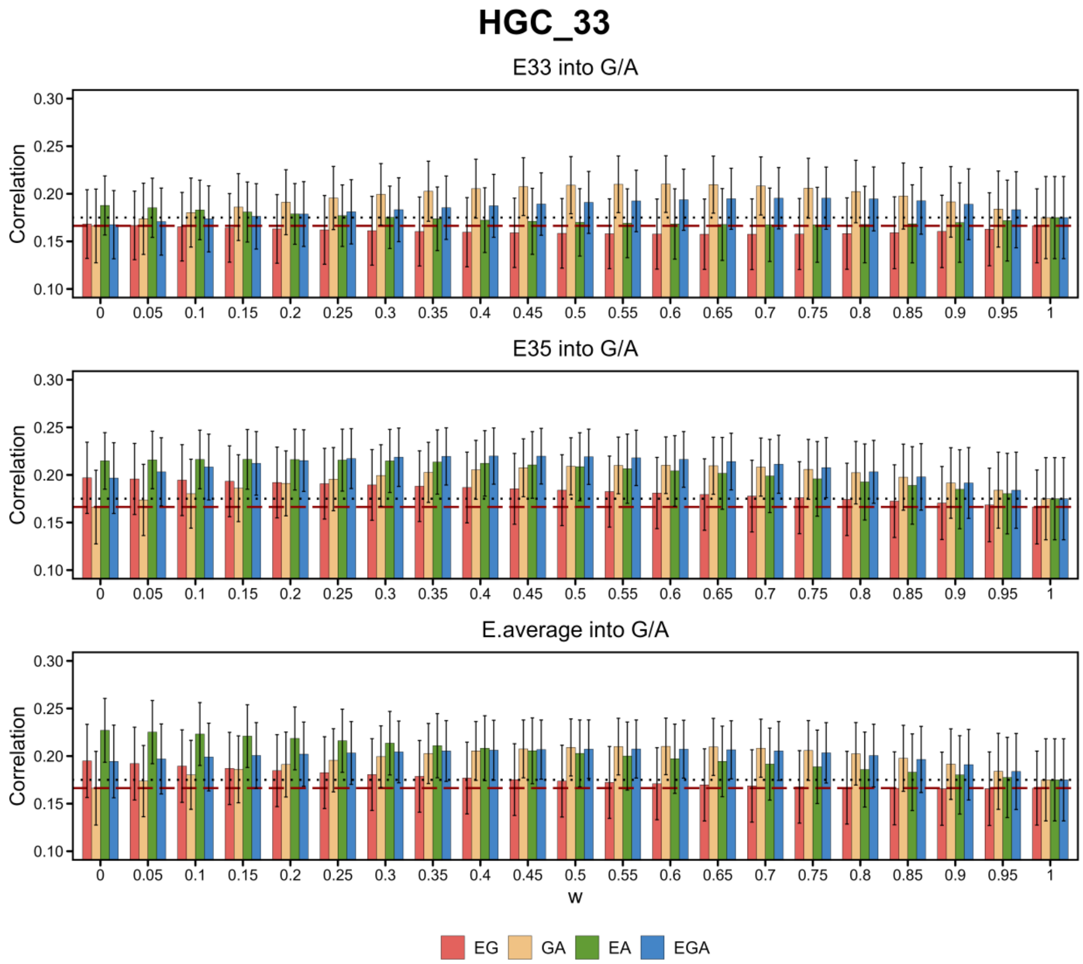

Figure S21. The Prediction Accuracy for trait of HGC_33

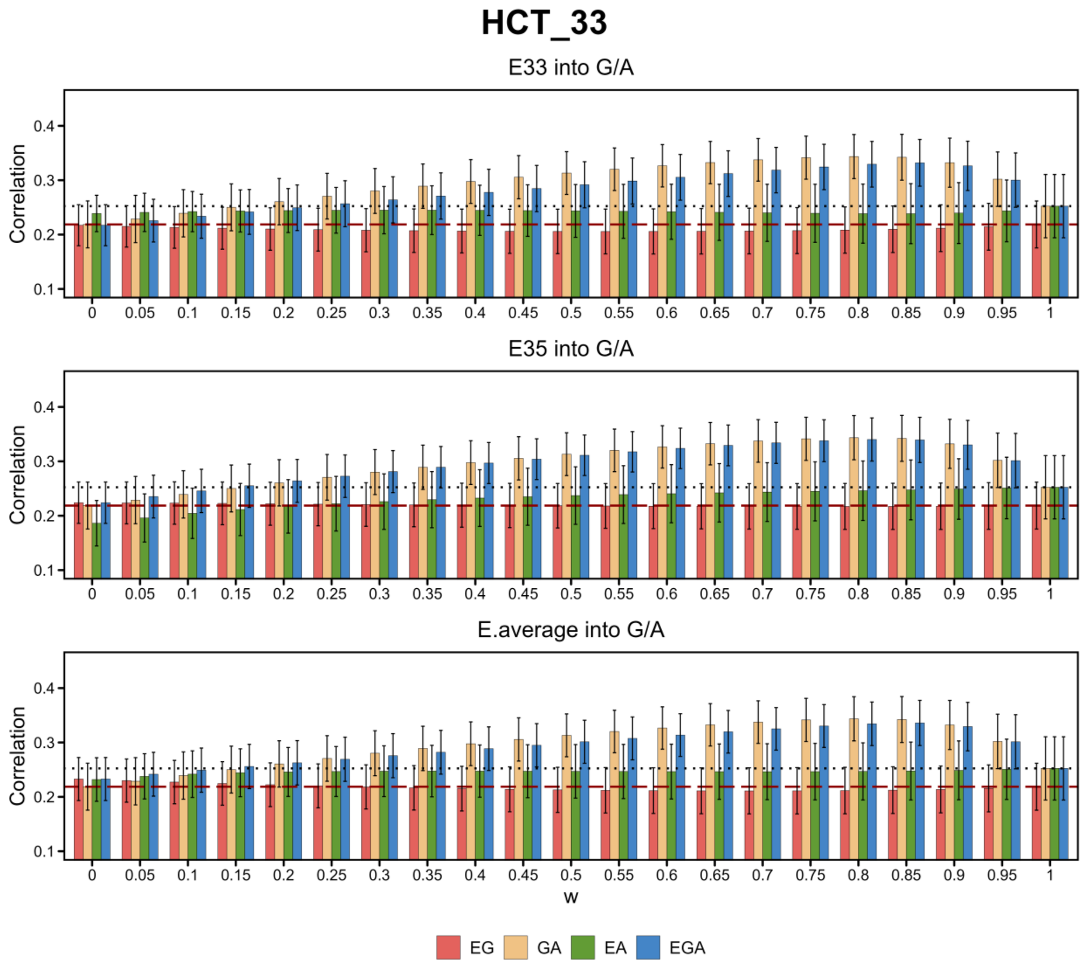

Figure S22. The Prediction Accuracy for trait of HCT_33

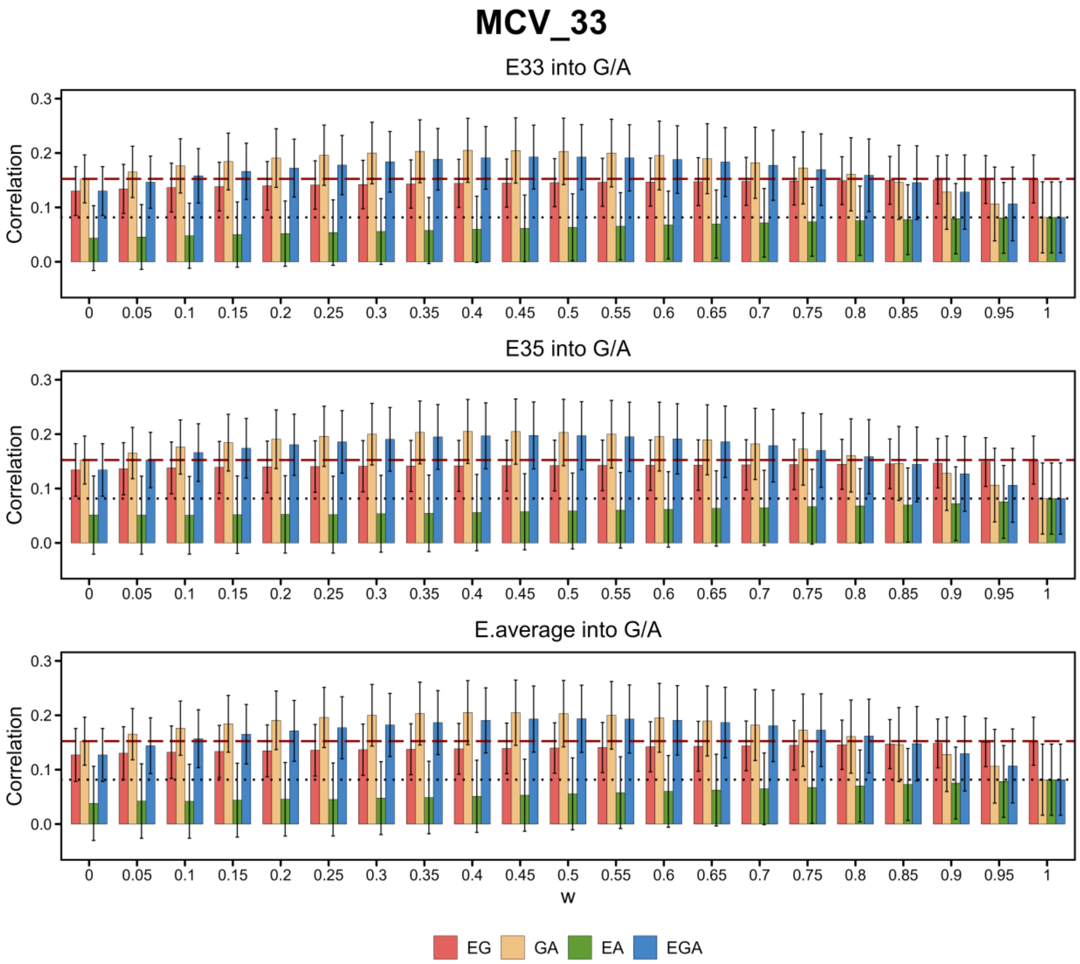

Figure S23. The Prediction Accuracy for trait of MCV_33

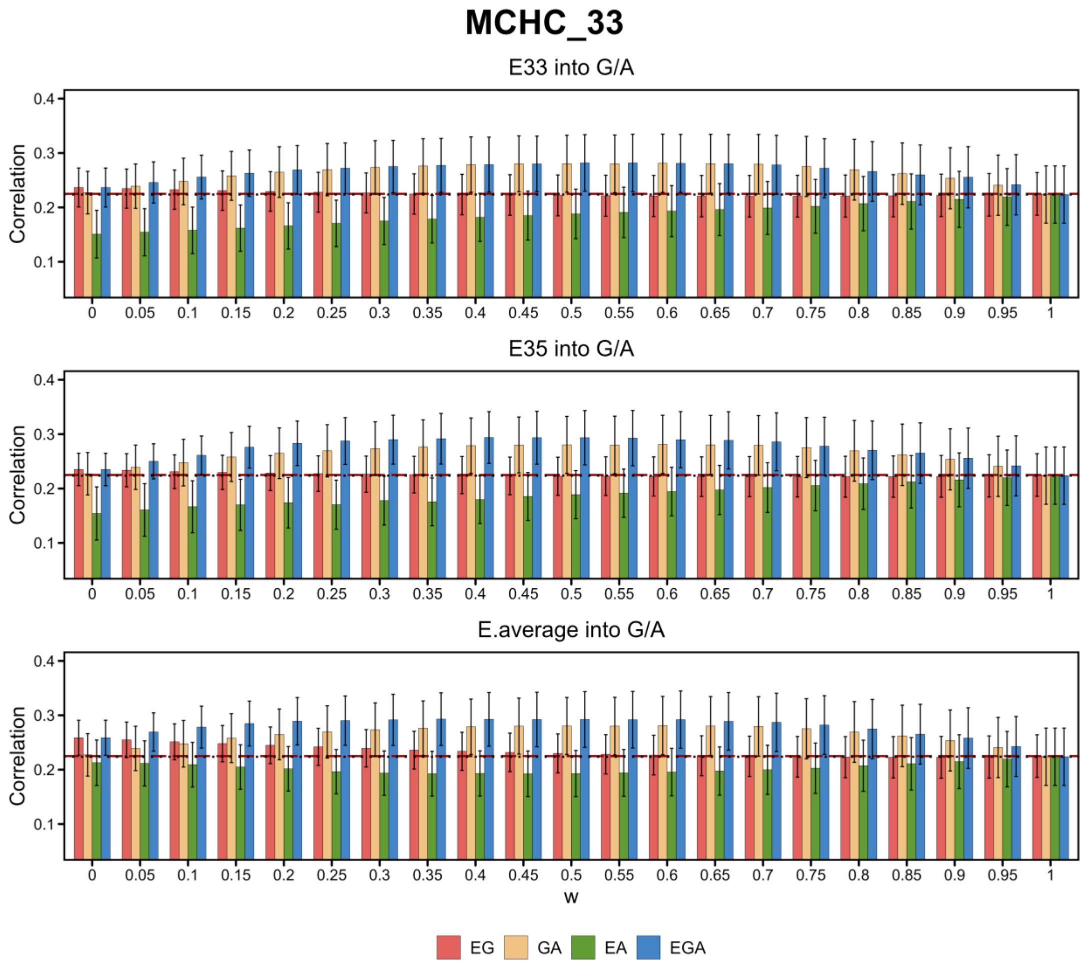

Figure S24. The Prediction Accuracy for trait of MCHC_33

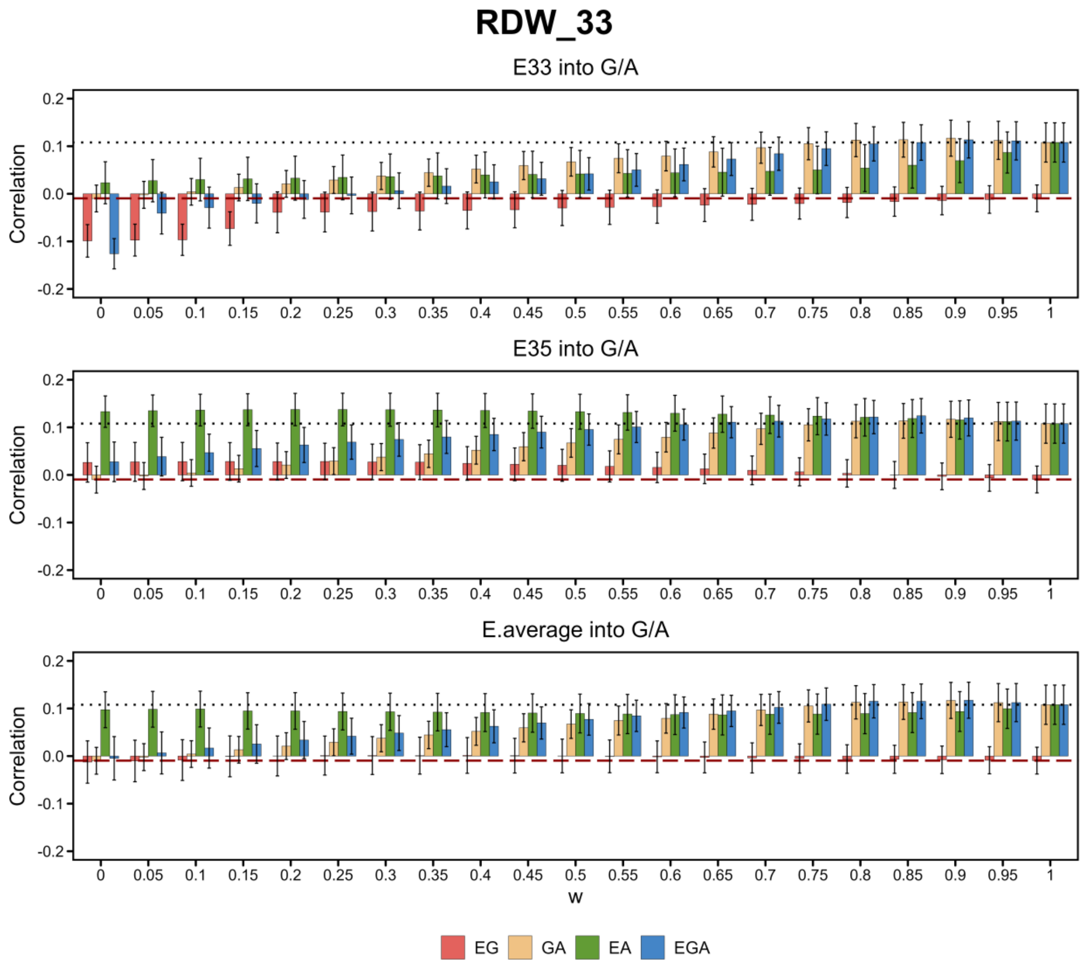

Figure S25. The Prediction Accuracy for trait of RDW_33

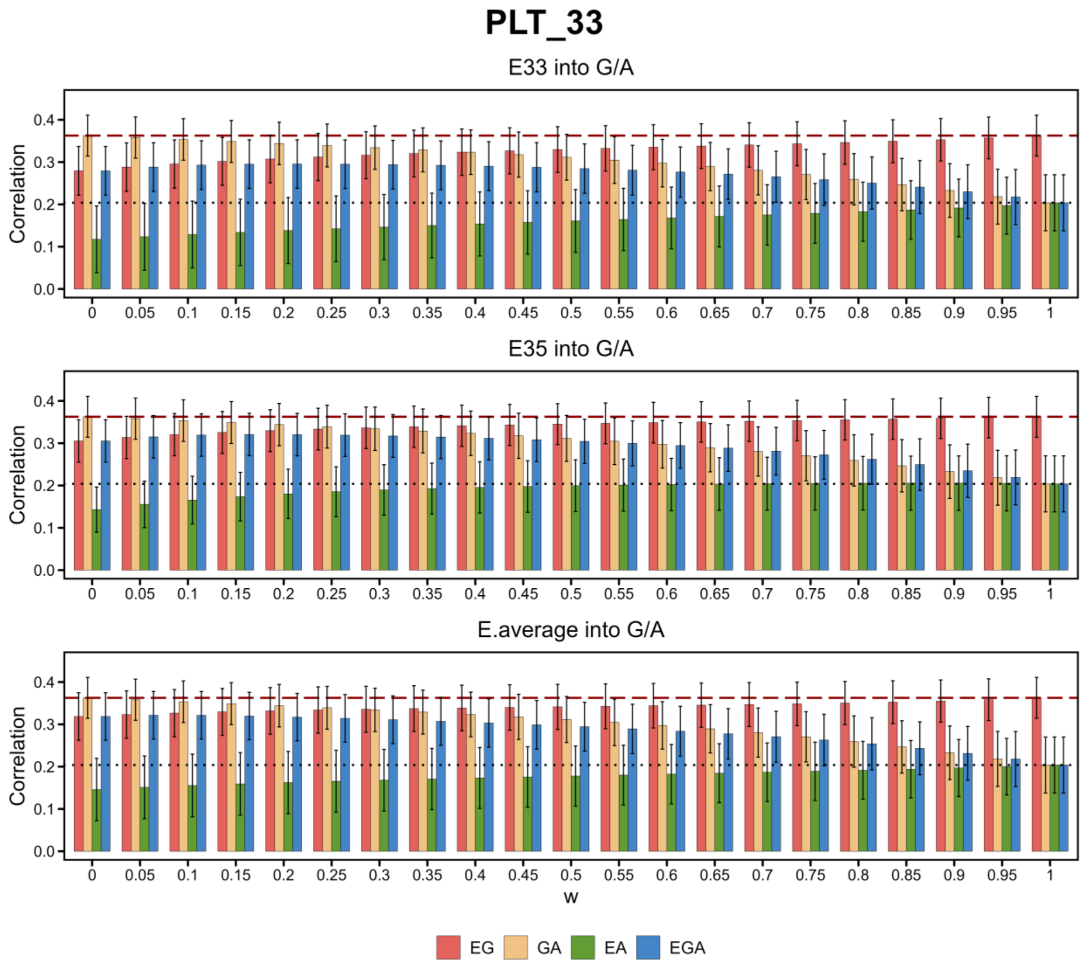

Figure S26. The Prediction Accuracy for trait of PLT_33

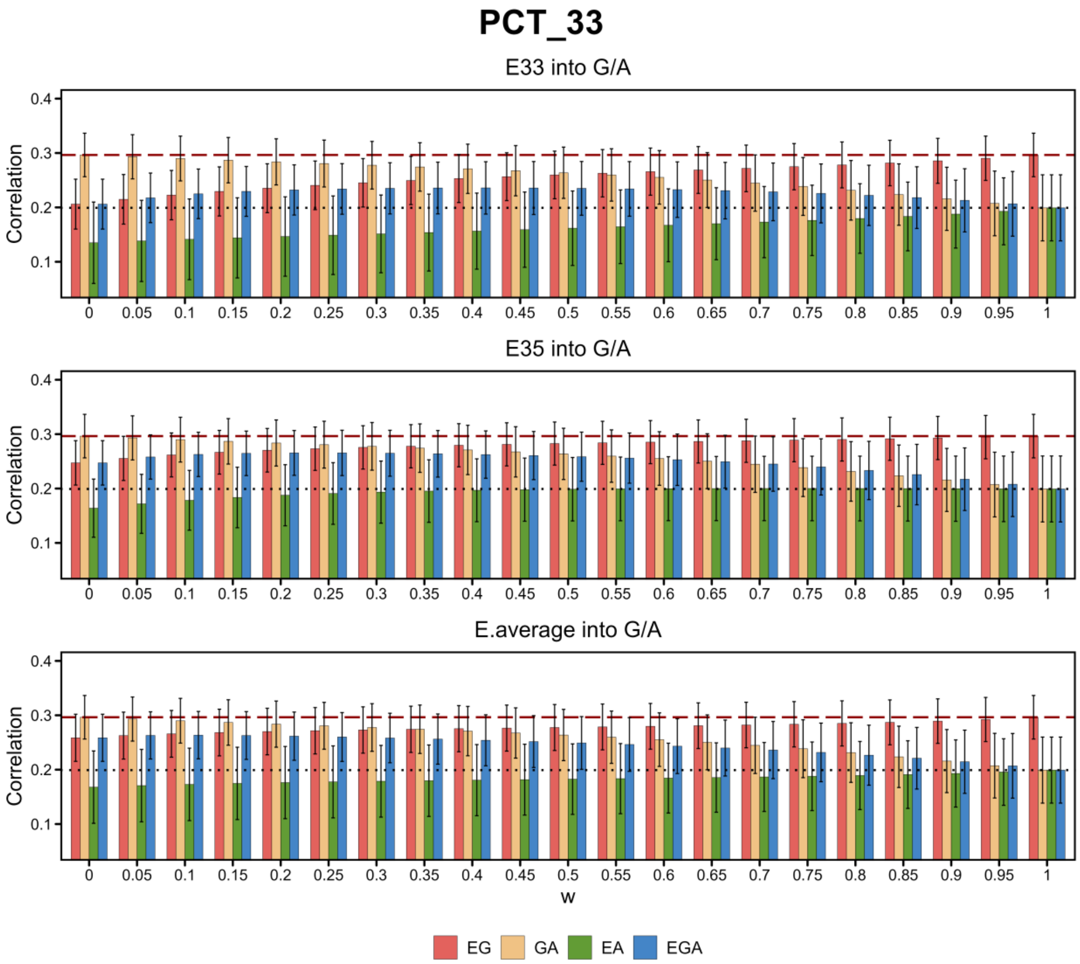

Figure S27. The Prediction Accuracy for trait of PCT_33

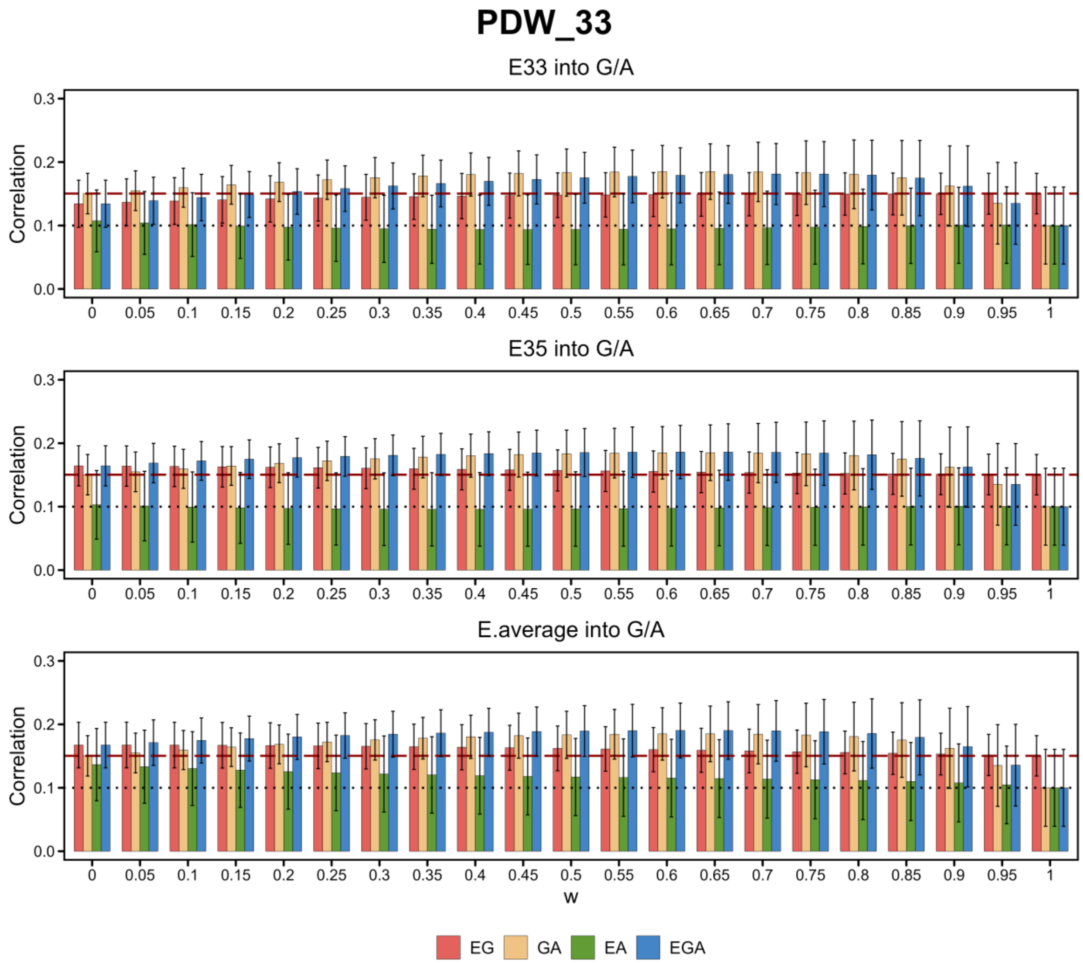

Figure S28. The Prediction Accuracy for trait of PDW_33

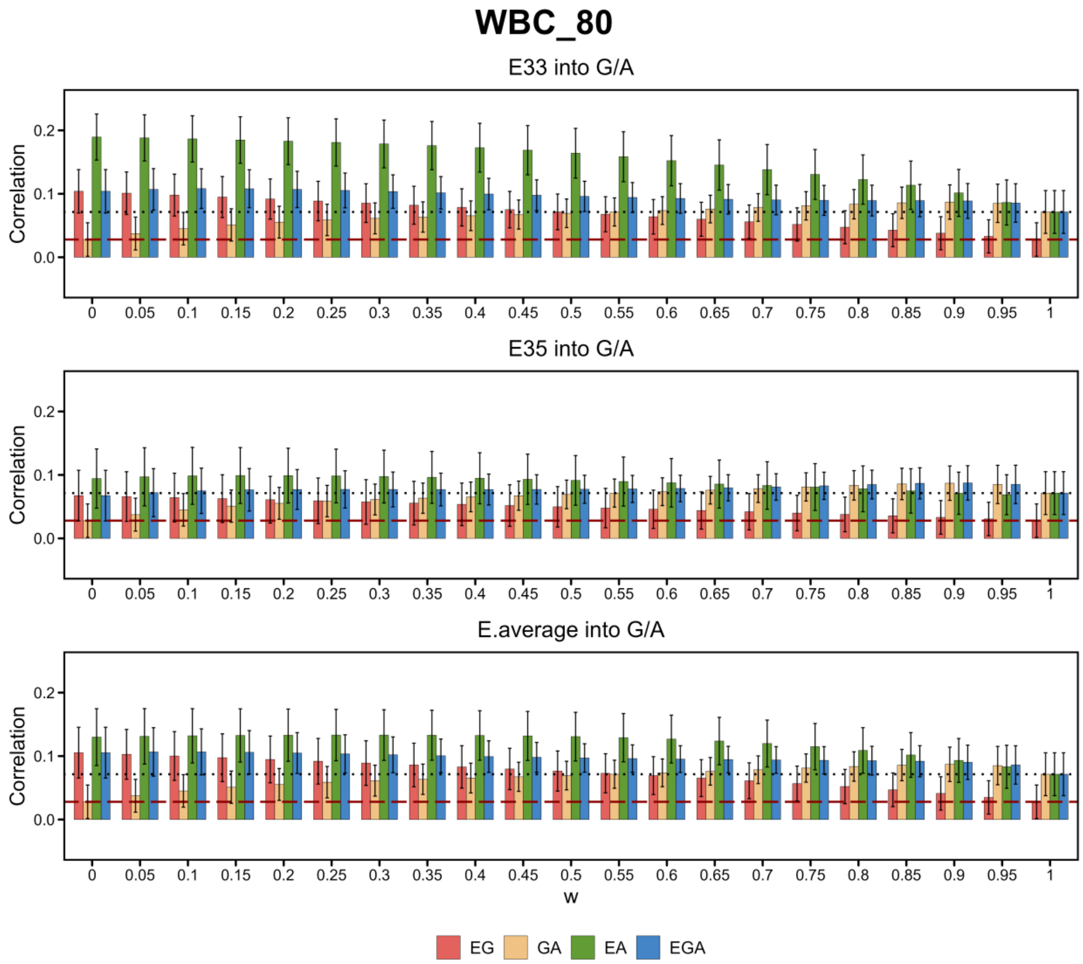

Figure S29. The Prediction Accuracy for trait of WBC_80

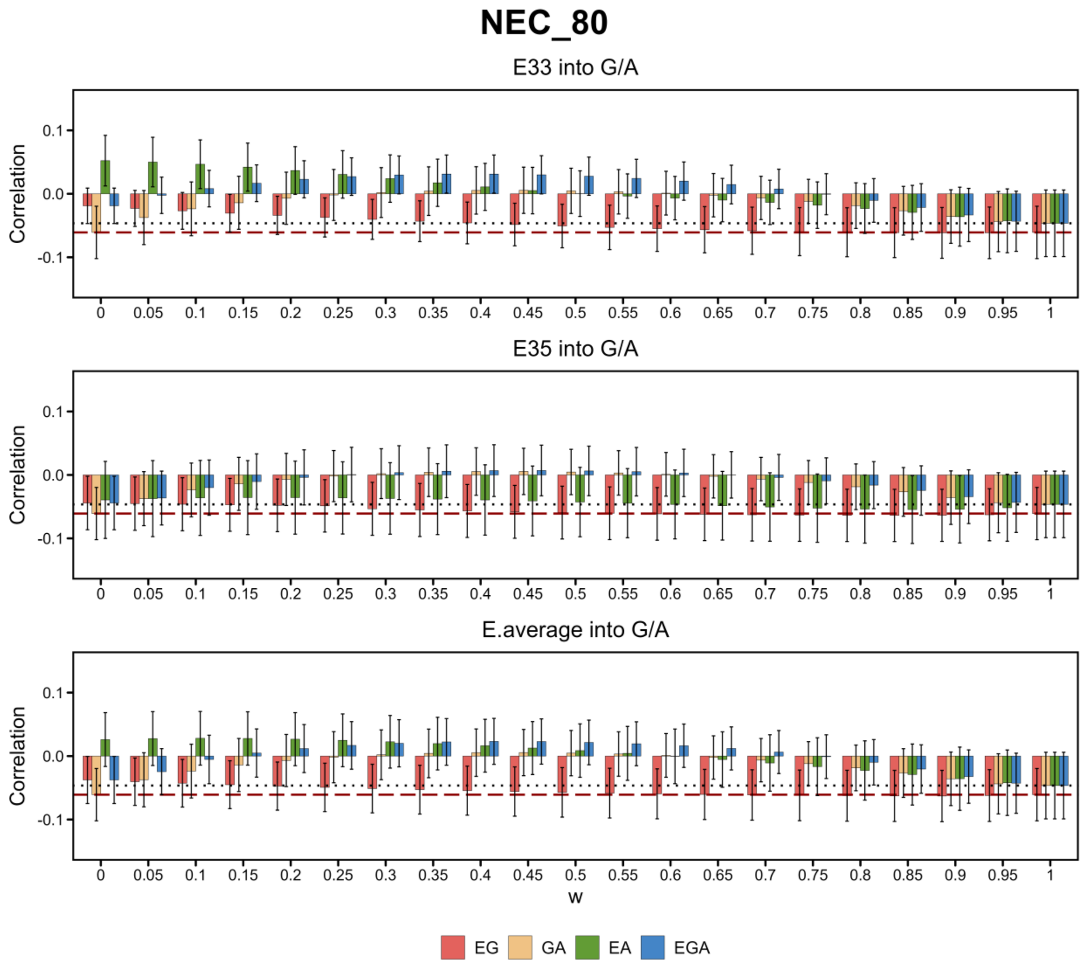

Figure S30. The Prediction Accuracy for trait of NEC_80

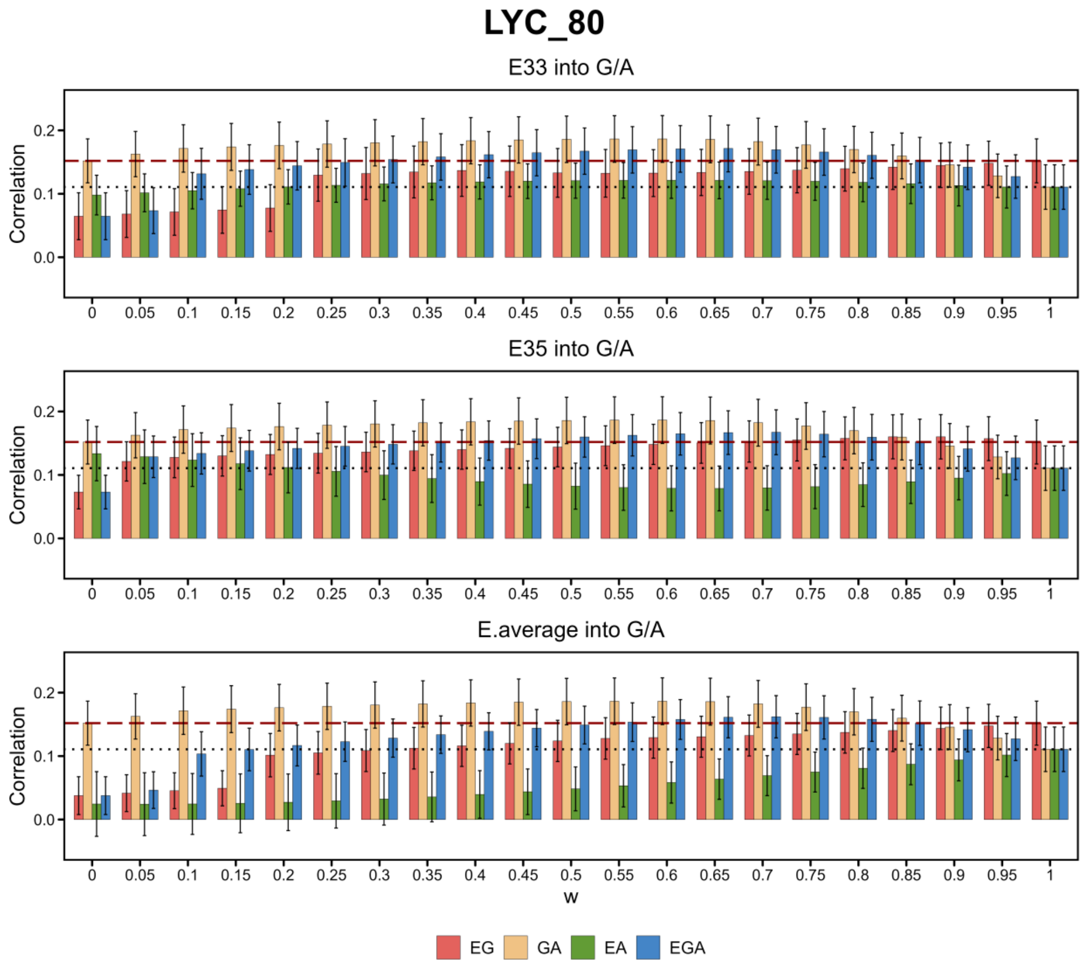

Figure S31. The Prediction Accuracy for trait of LYC_80

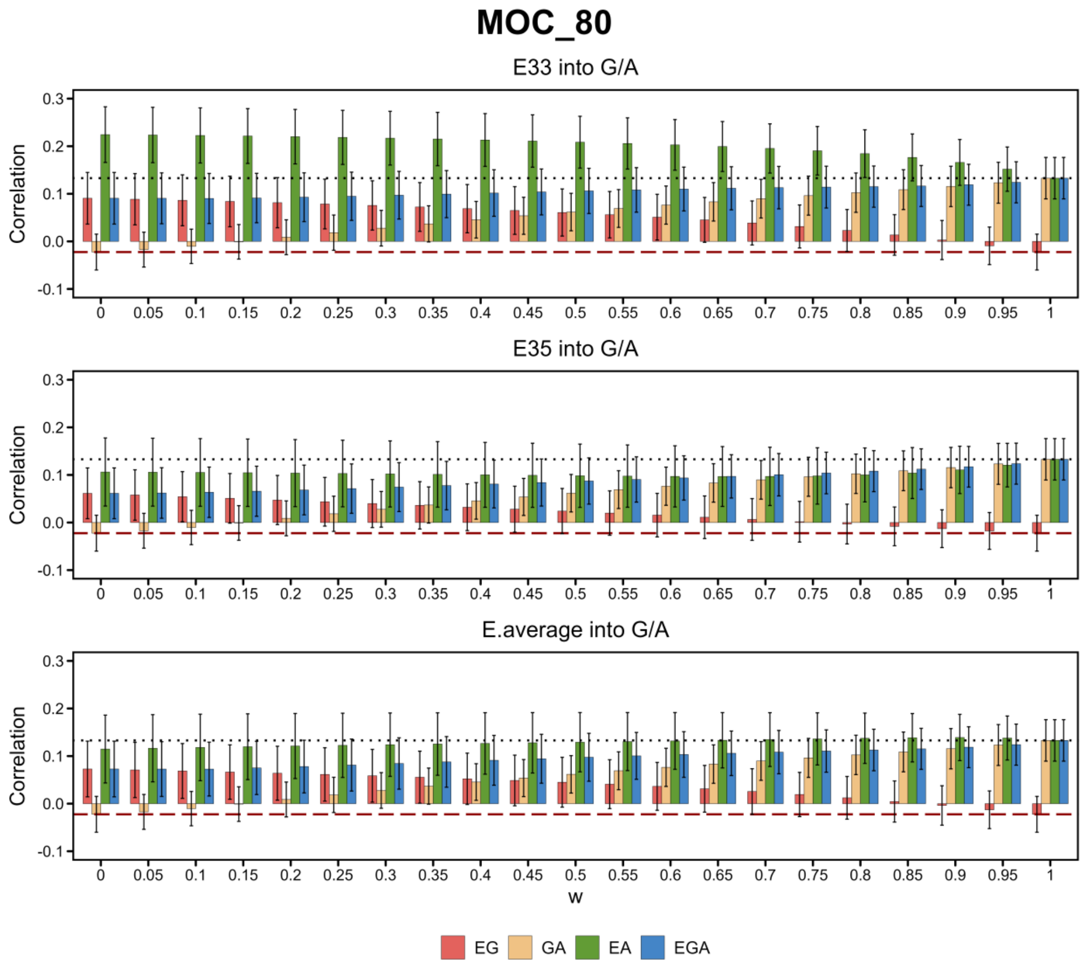

Figure S32. The Prediction Accuracy for trait of MOC_80

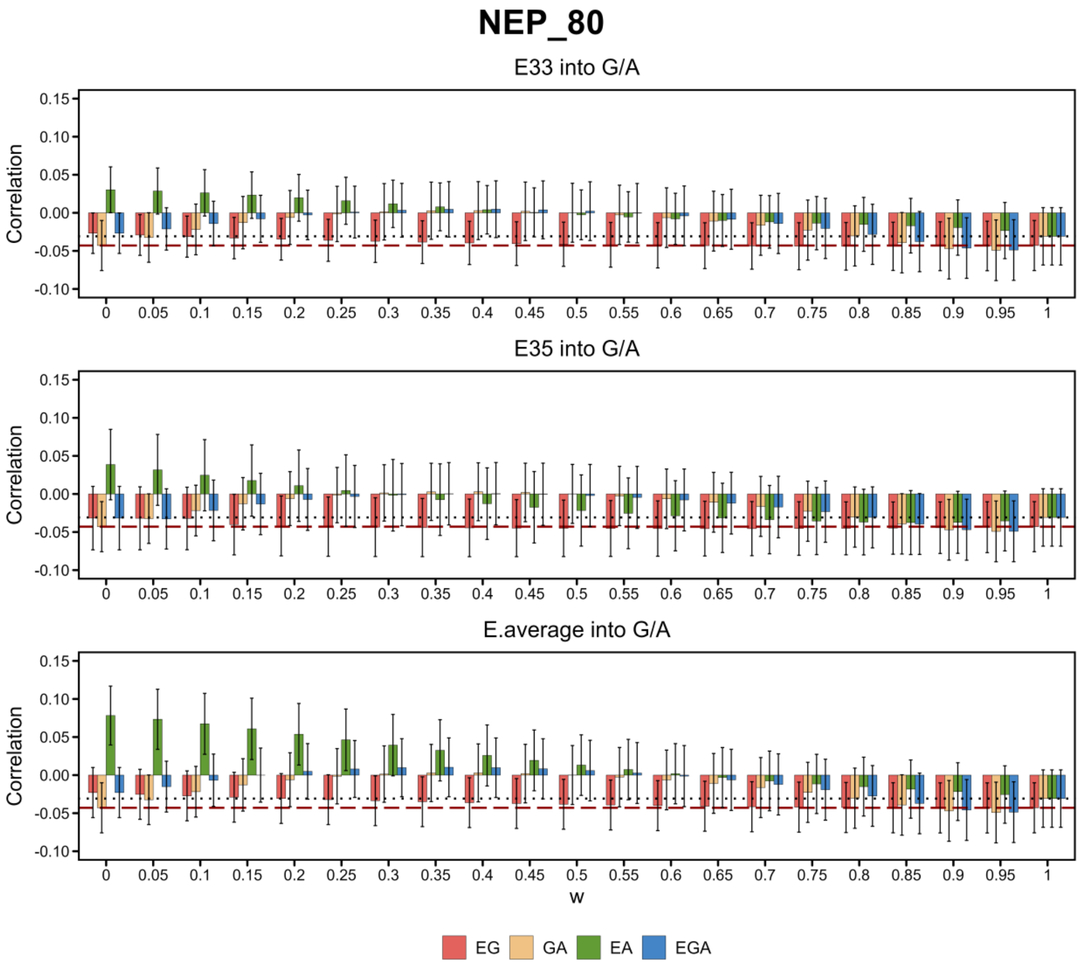

Figure S33. The Prediction Accuracy for trait of NEP_80

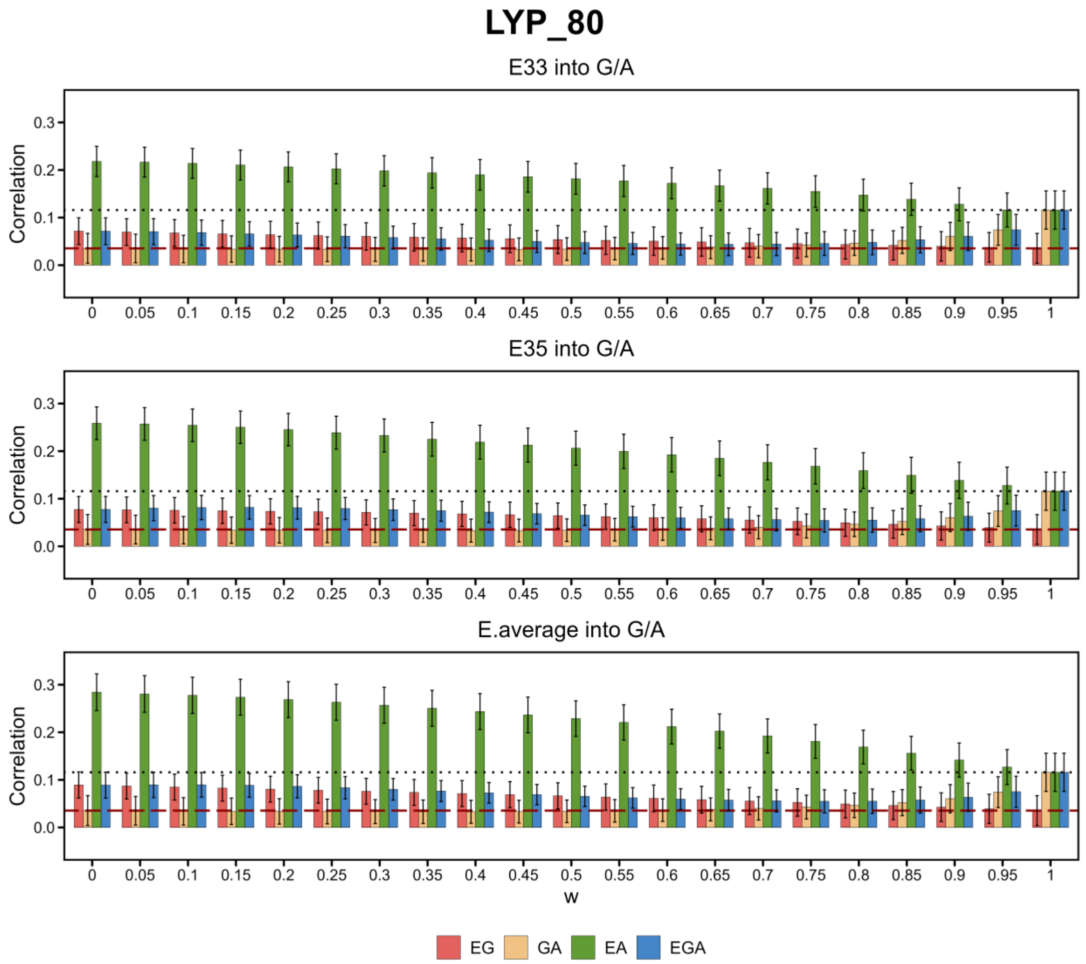

Figure S34. The Prediction Accuracy for trait of LYP_80

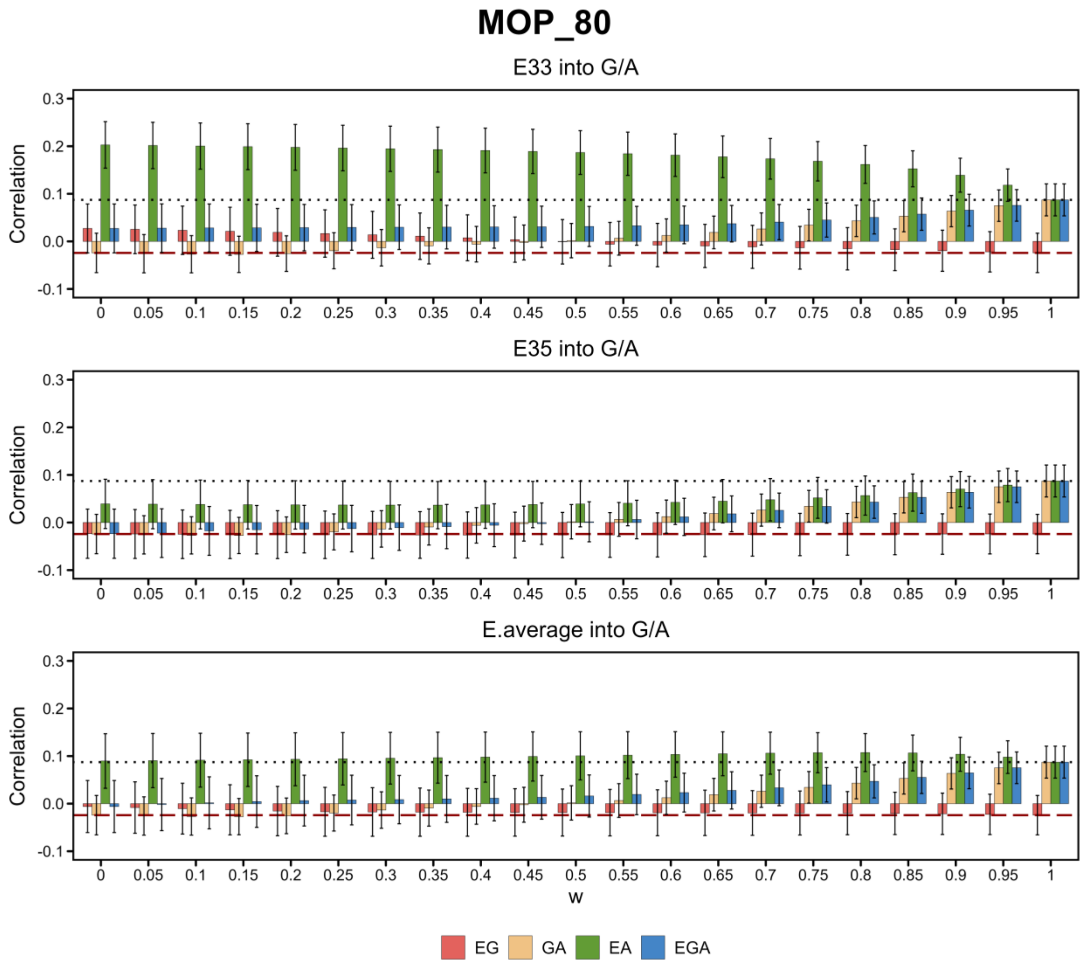

Figure S35. The Prediction Accuracy for trait of MOP_80

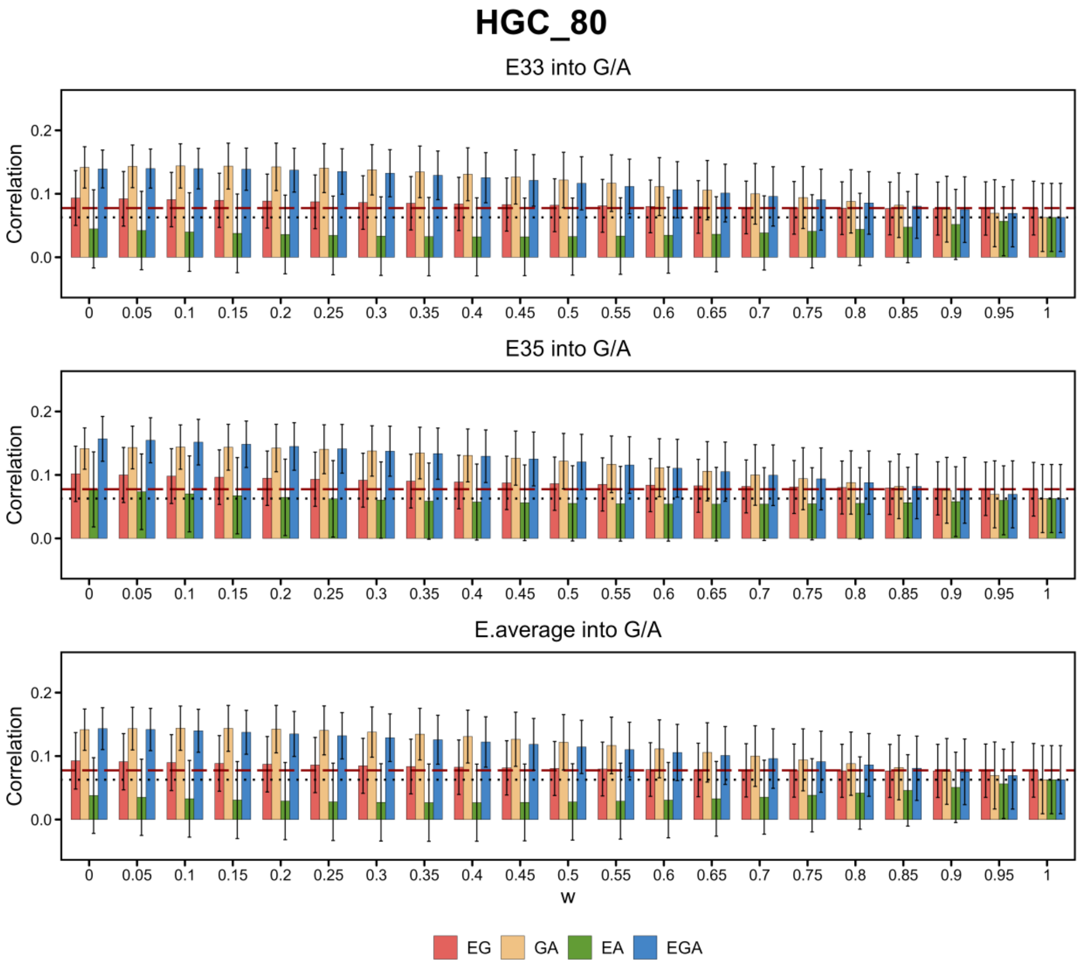

Figure S36. The Prediction Accuracy for trait of HGC_80

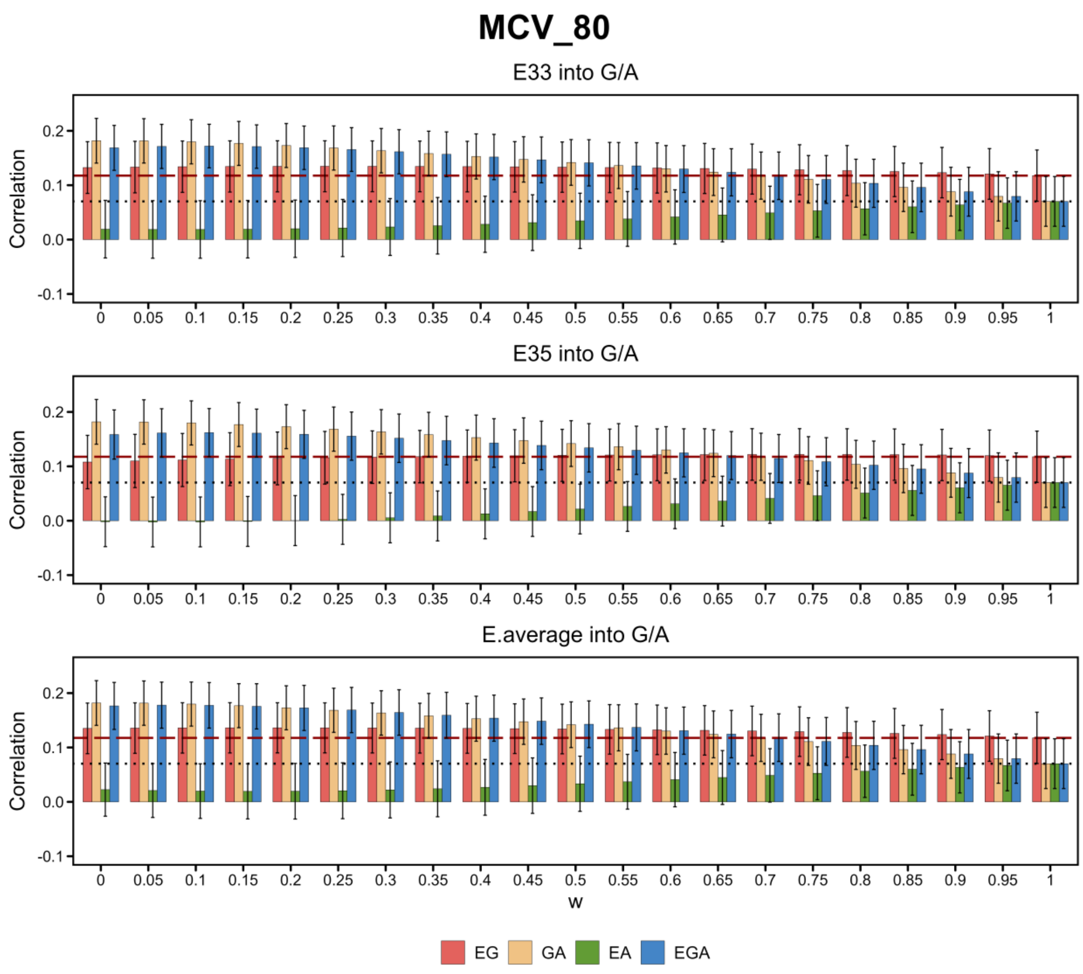

Figure S37. The Prediction Accuracy for trait of MCV_80

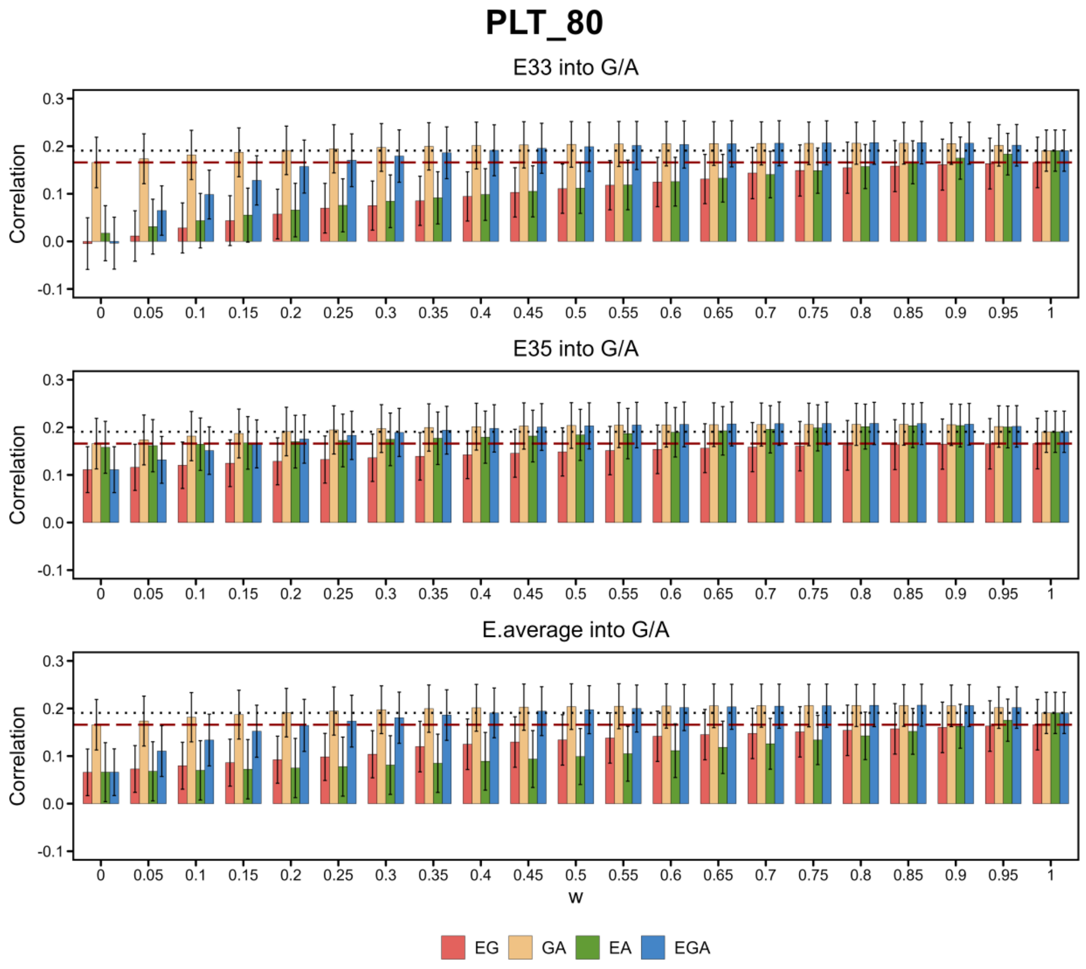

Figure S38. The Prediction Accuracy for trait of PLT_80

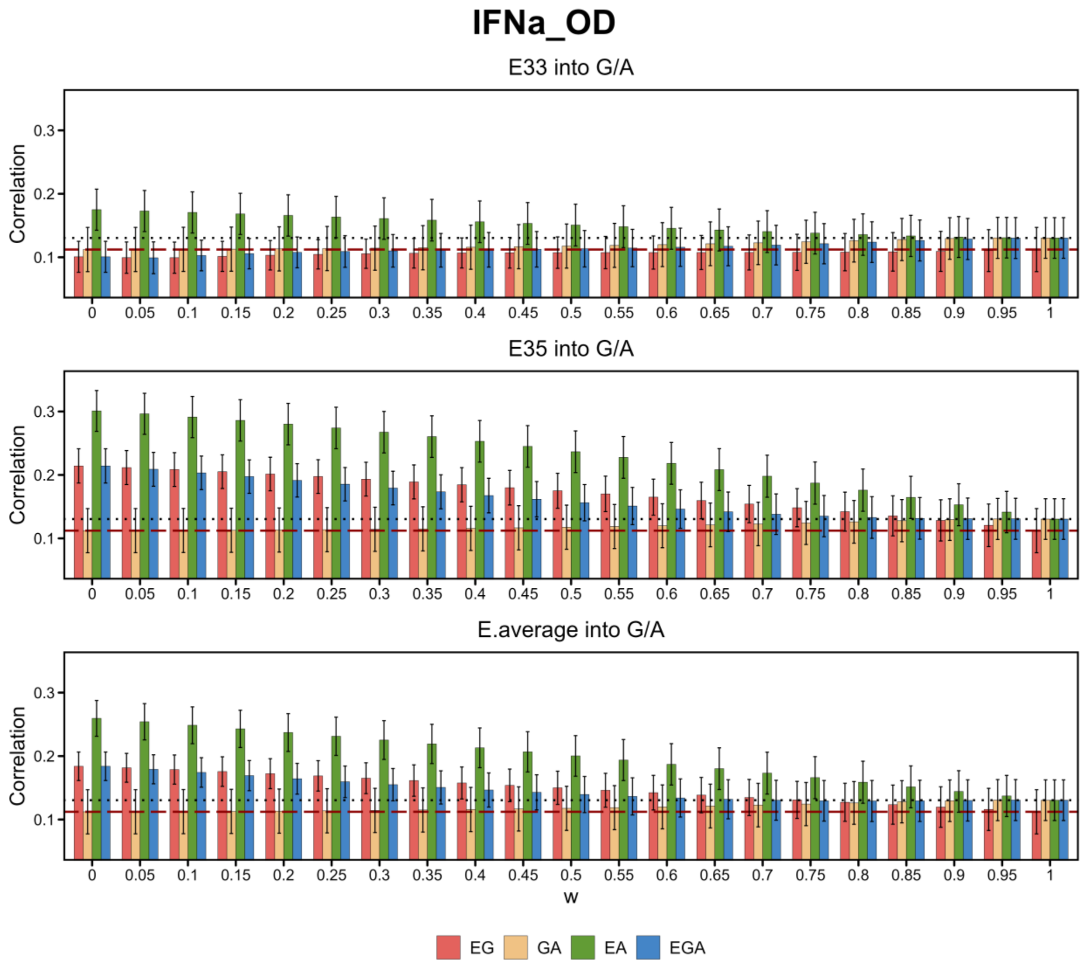

Figure S39. The Prediction Accuracy for trait of IFNa_OD

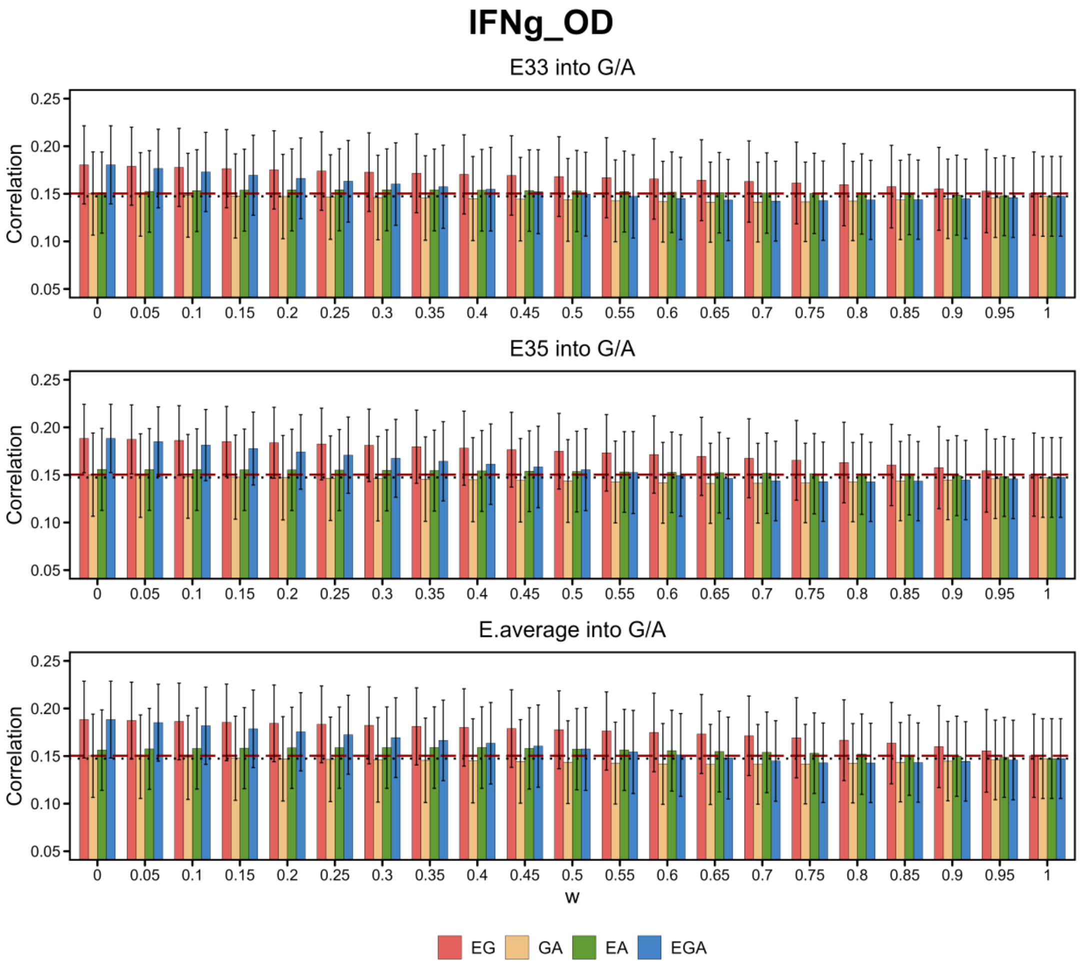

Figure S40. The Prediction Accuracy for trait of IFNg_OD

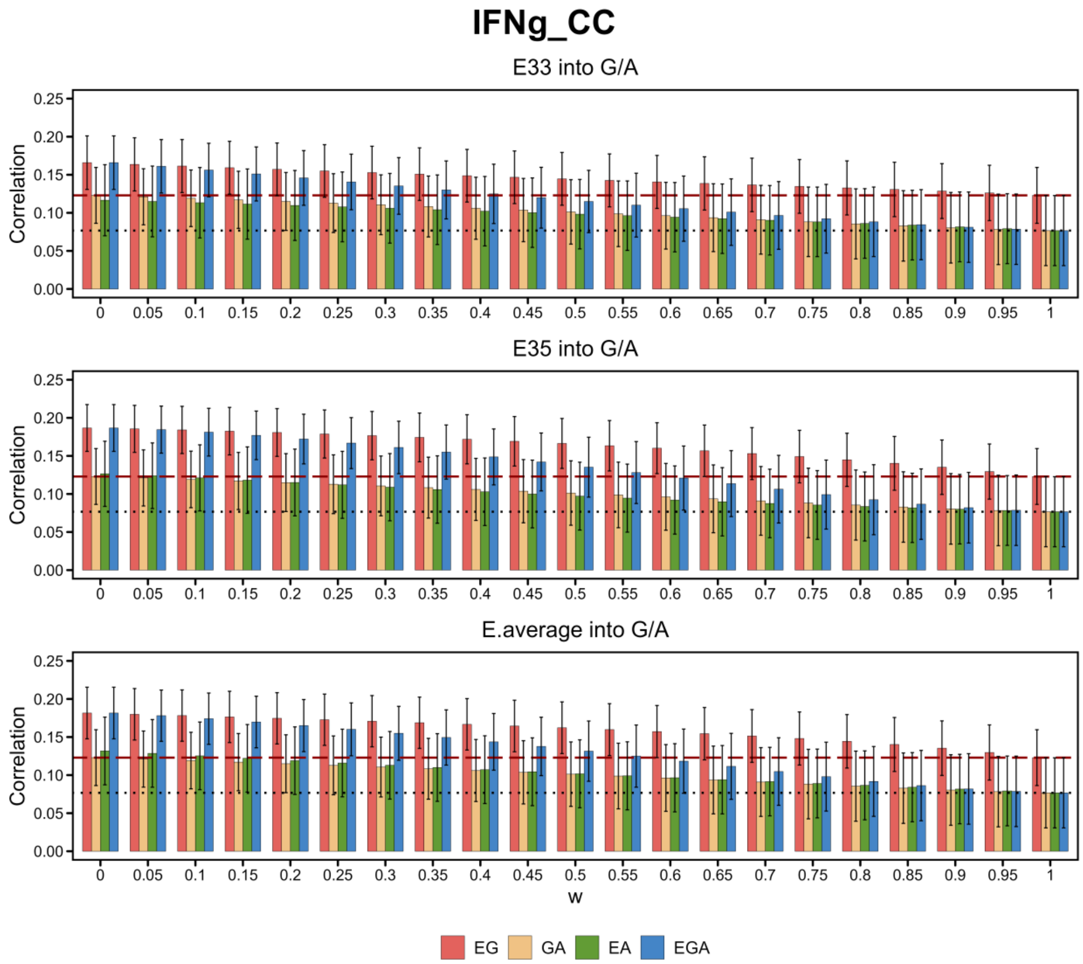

Figure S41. The Prediction Accuracy for trait of IFNg_CC

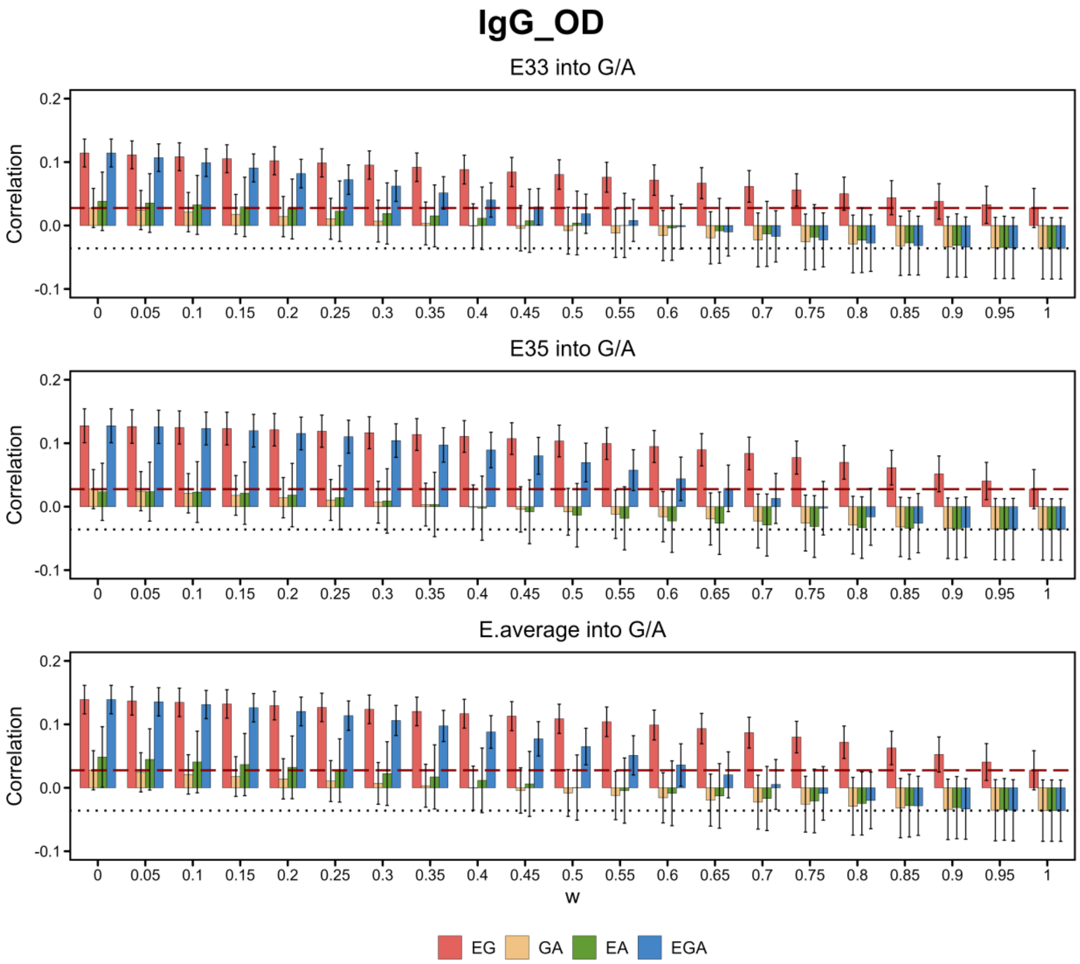

Figure S42. The Prediction Accuracy for trait of IgG_OD

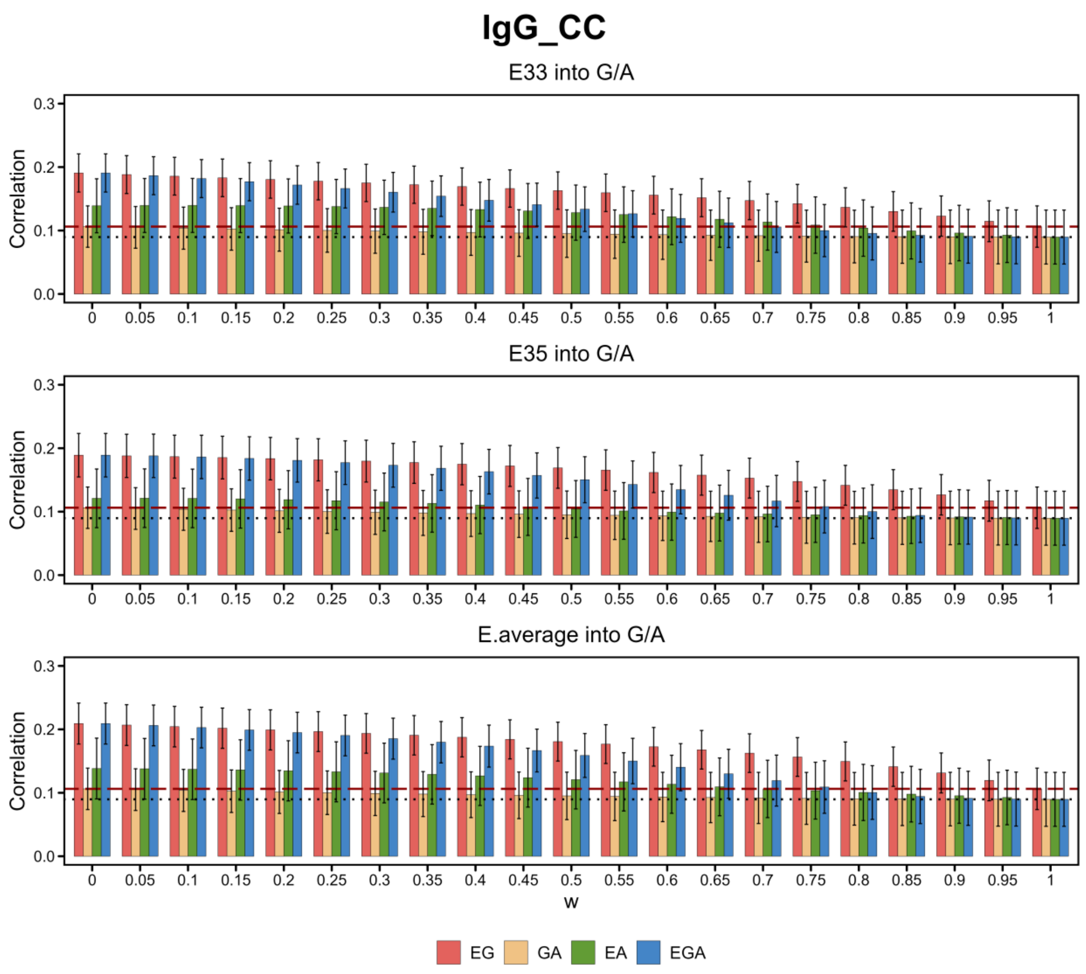

Figure S43. The Prediction Accuracy for trait of IgG_CC

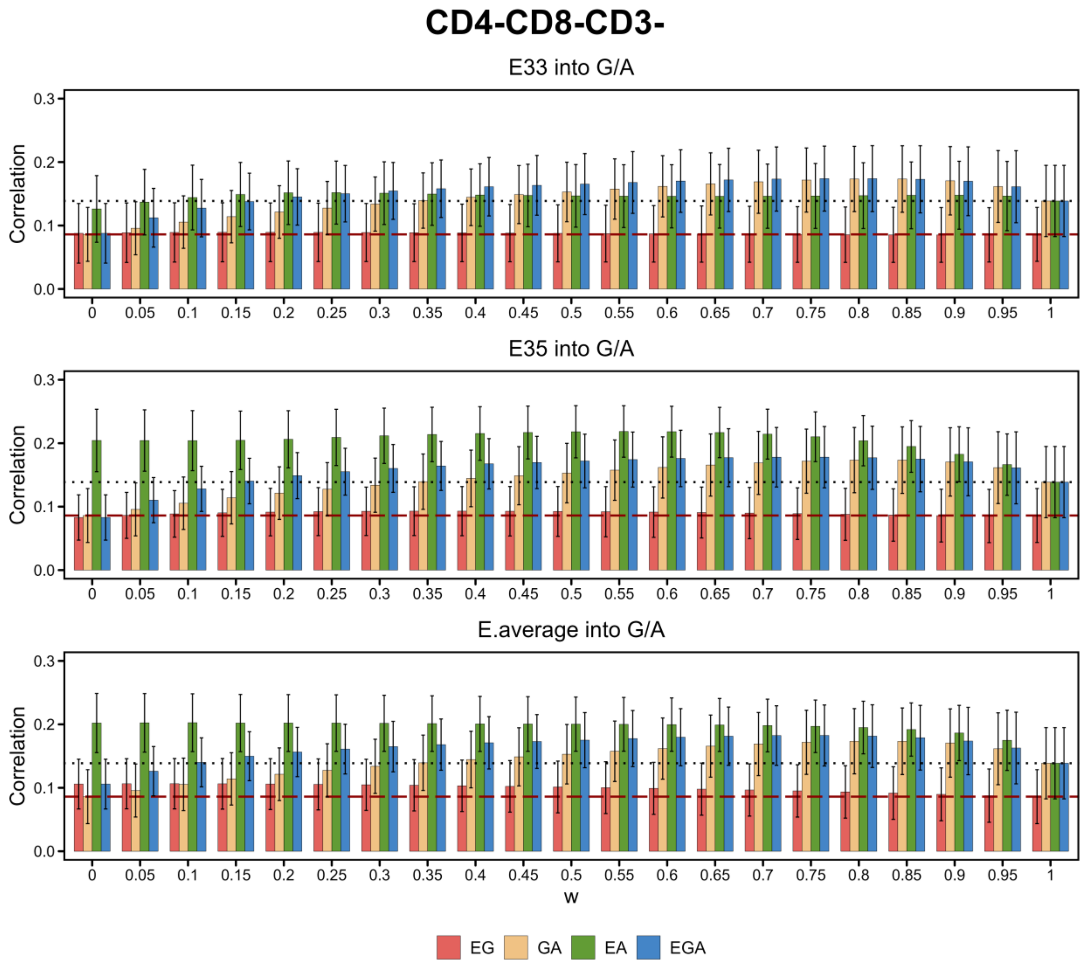

Figure S44. The Prediction Accuracy for trait of CD4-CD8-CD3-

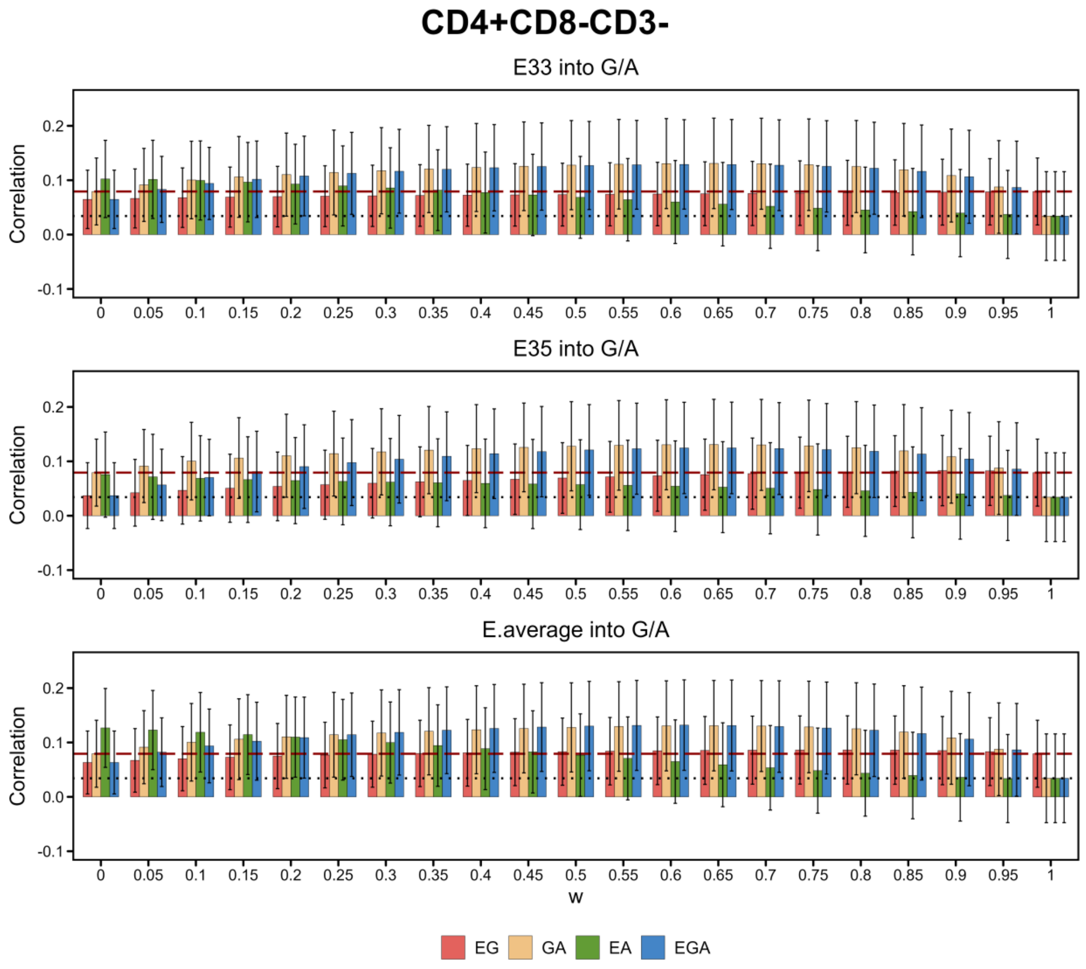

Figure S45. The Prediction Accuracy for trait of CD4+CD8-CD3-

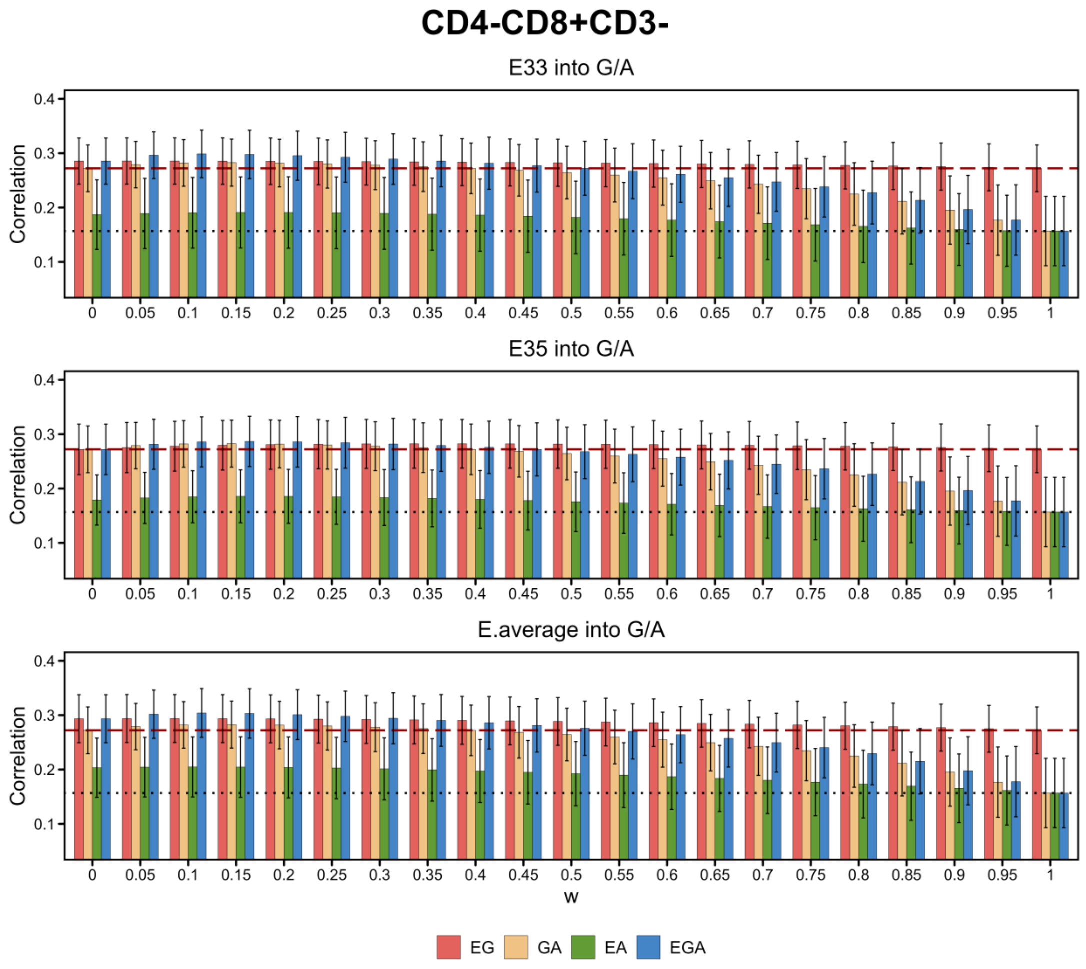

Figure S46. The Prediction Accuracy for trait of CD4-CD8+CD3-

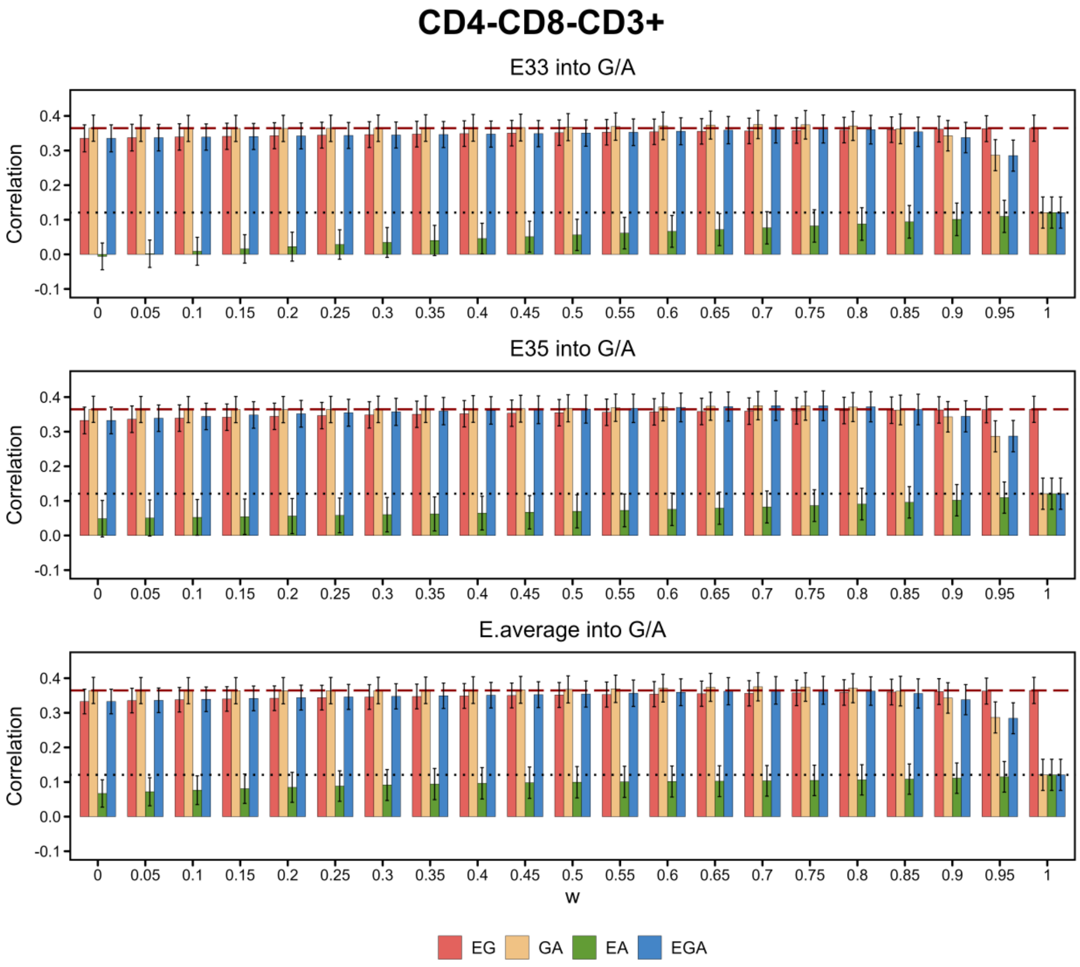

Figure S47. The Prediction Accuracy for trait of CD4-CD8-CD3+

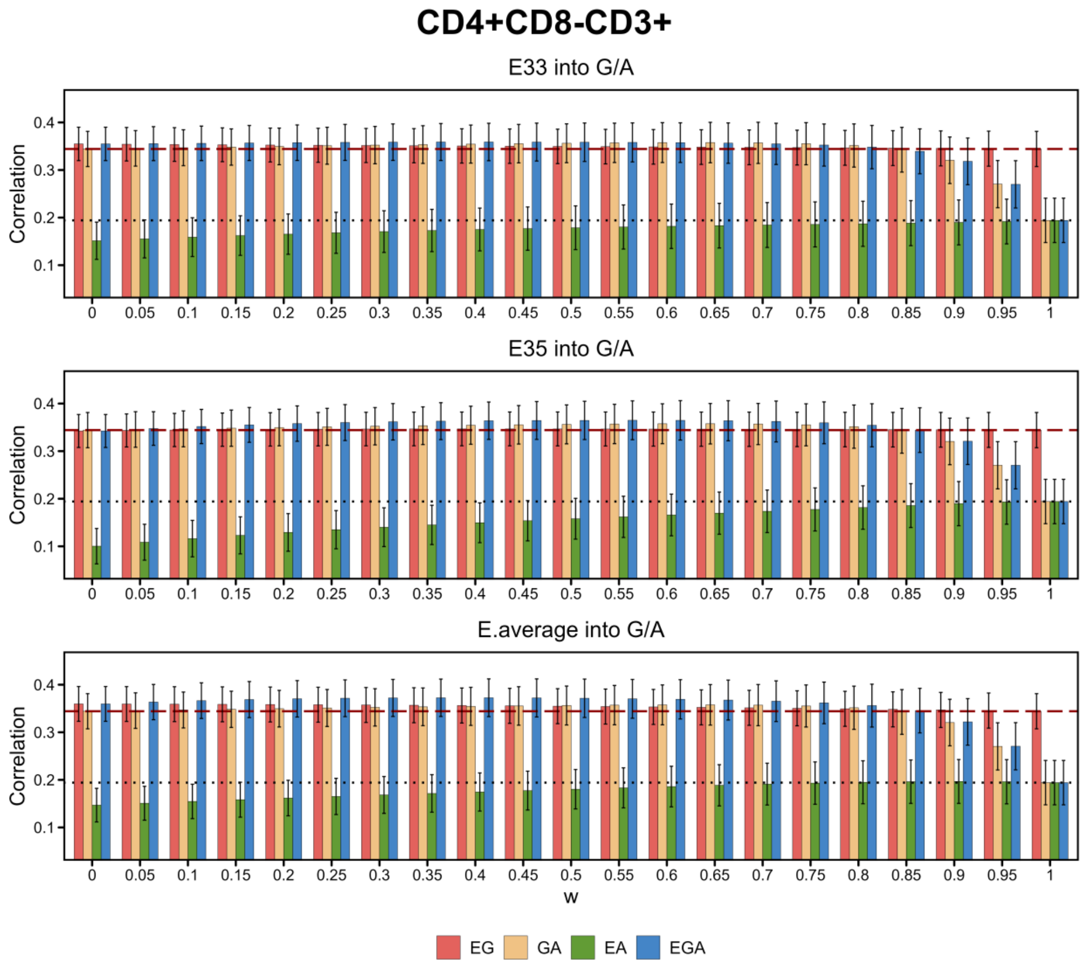

Figure S48. The Prediction Accuracy for trait of CD4+CD8-CD3+

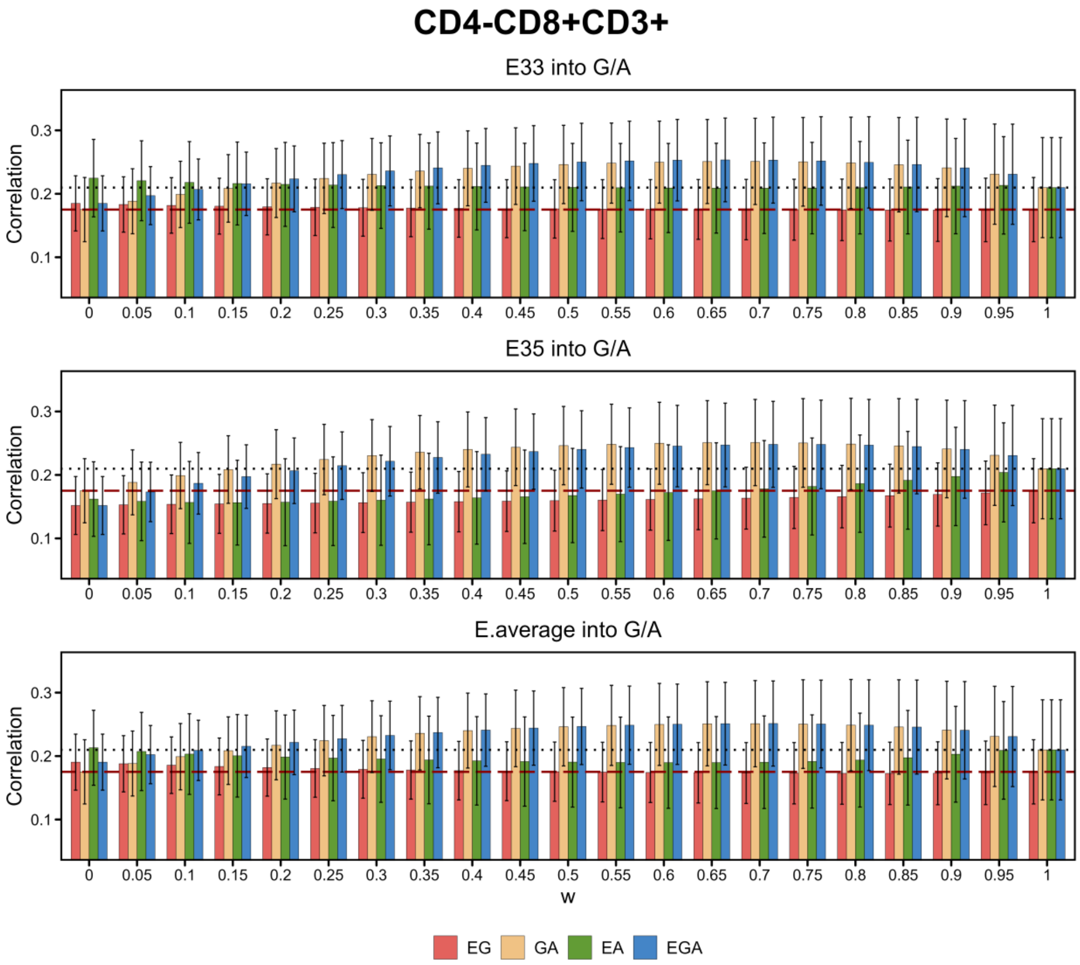

Figure S49. The Prediction Accuracy for trait of CD4-CD8+CD3+

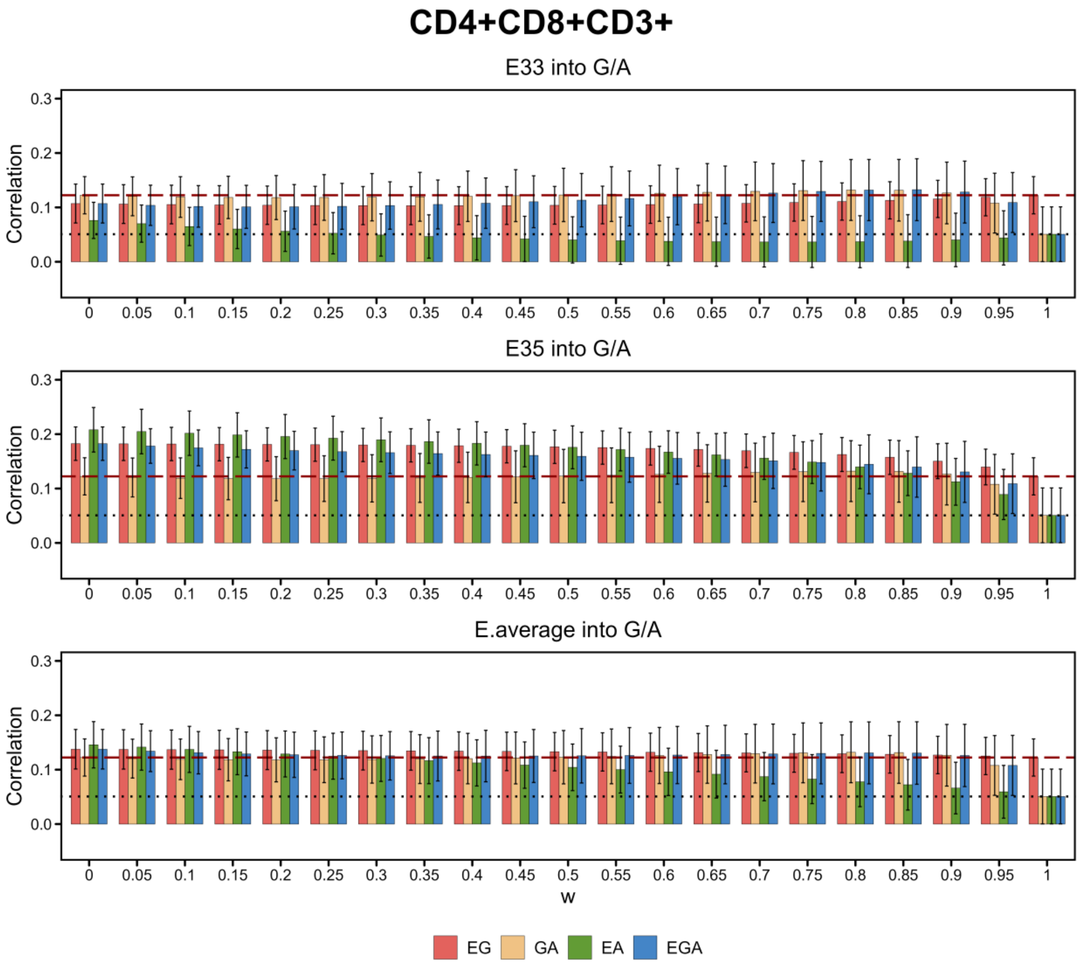

Figure S50. The Prediction Accuracy for trait of CD4+CD8+CD3+

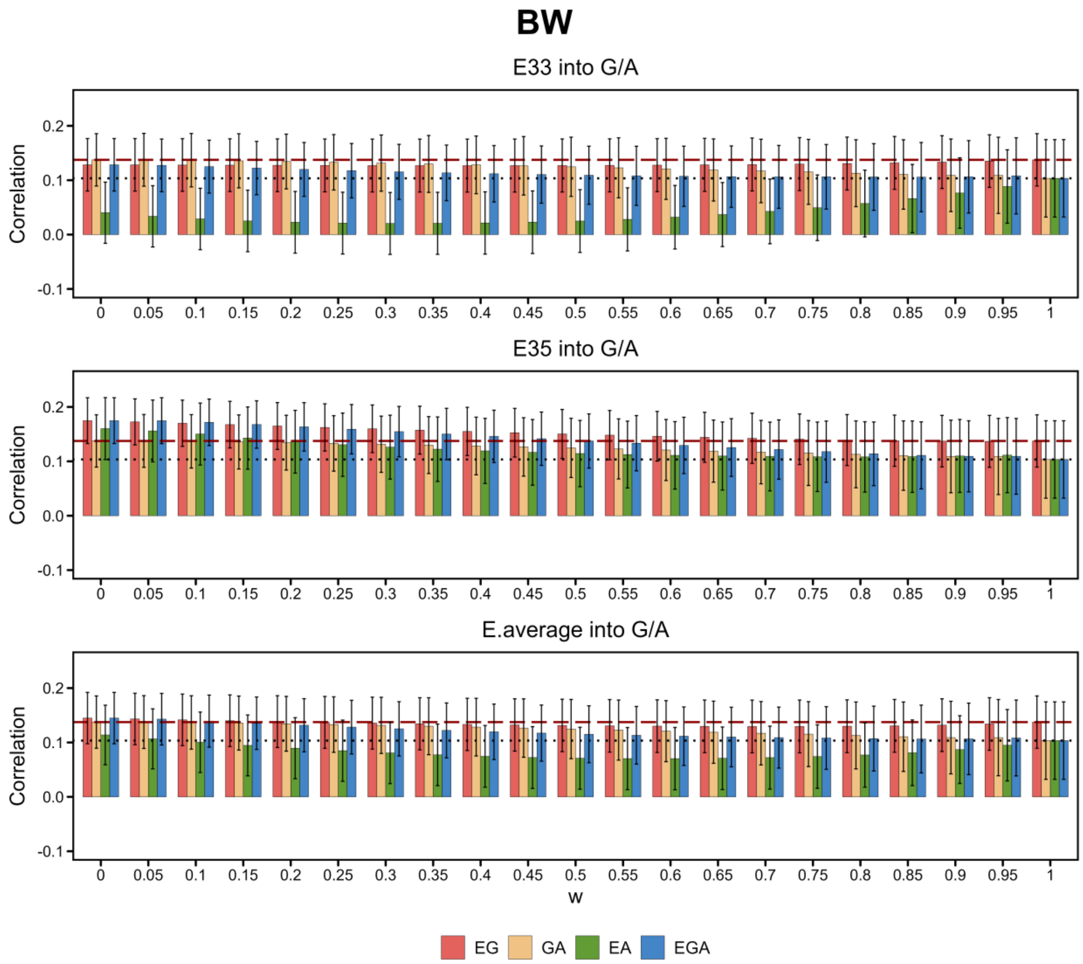

Figure S51. The Prediction Accuracy for trait of BW

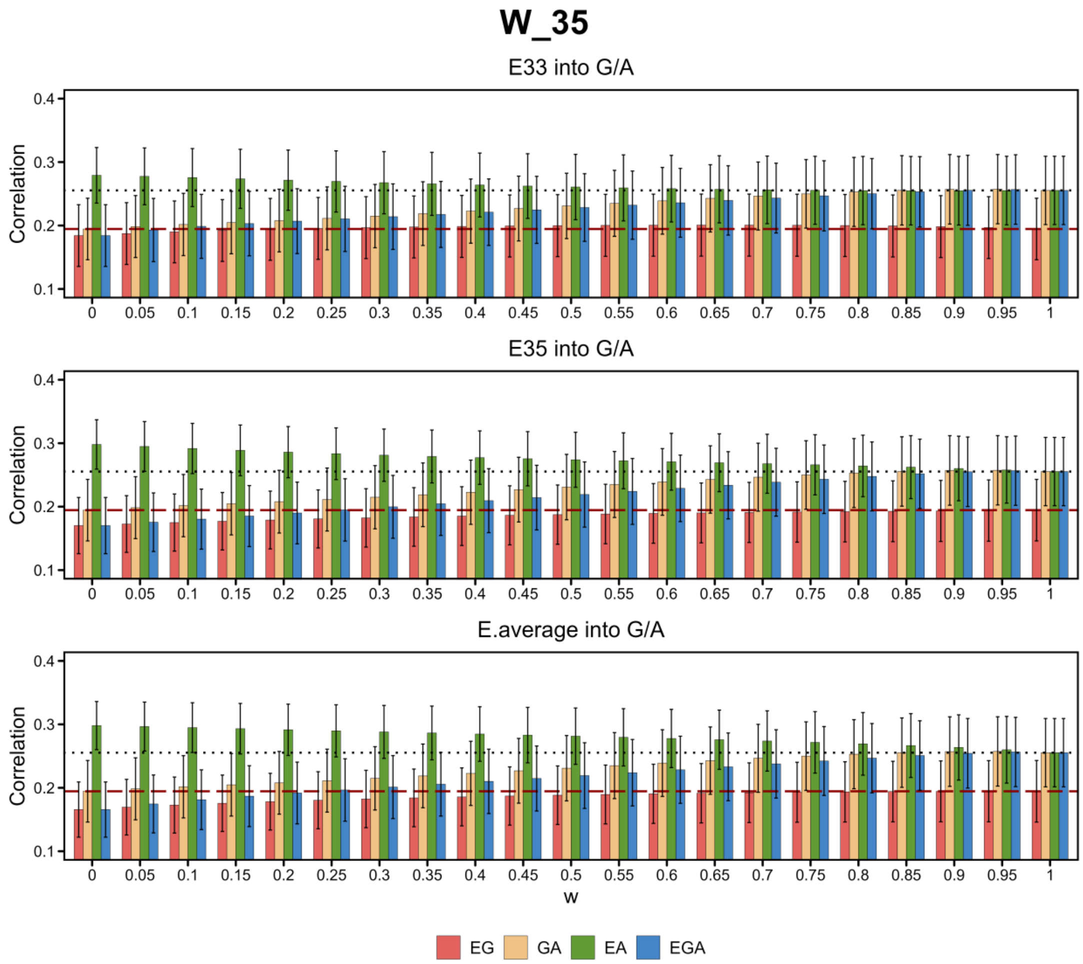

Figure S52. The Prediction Accuracy for trait of W_35

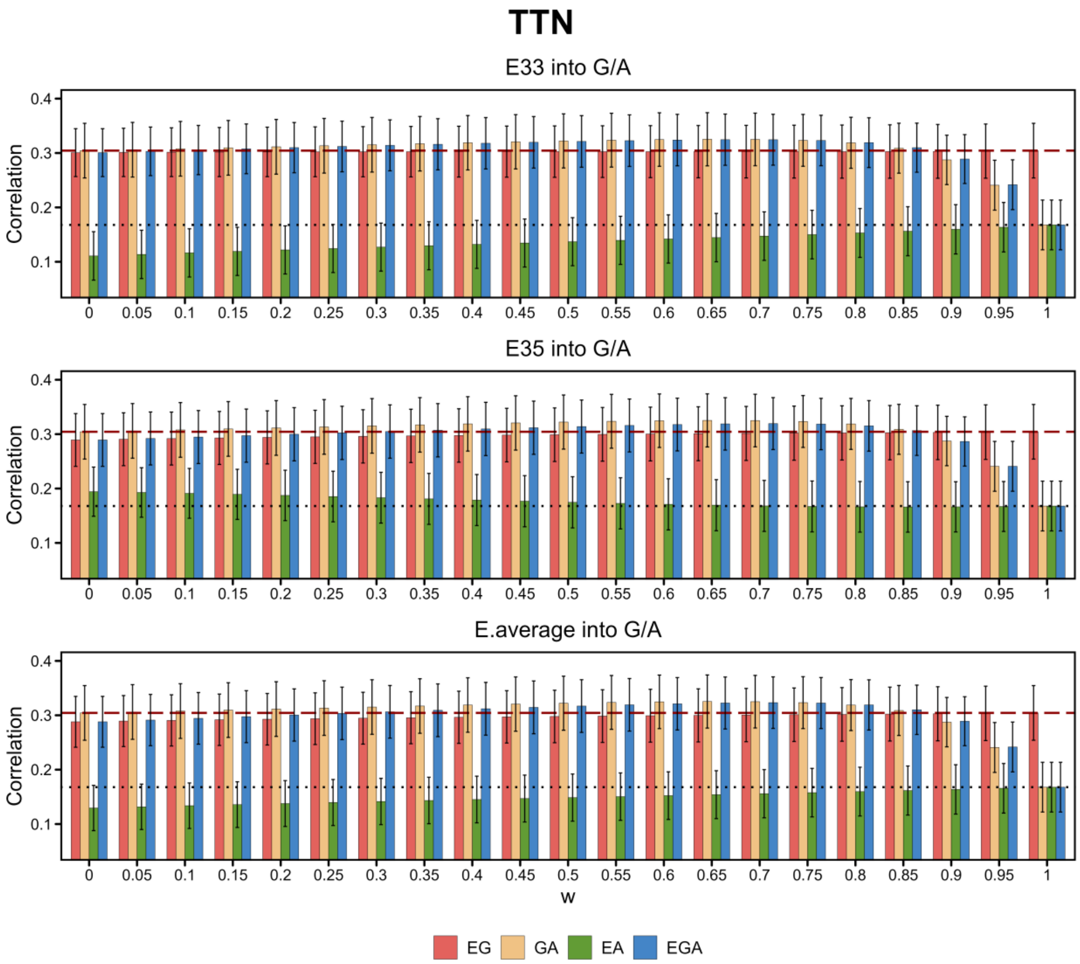

Figure S53. The Prediction Accuracy for trait of TTN

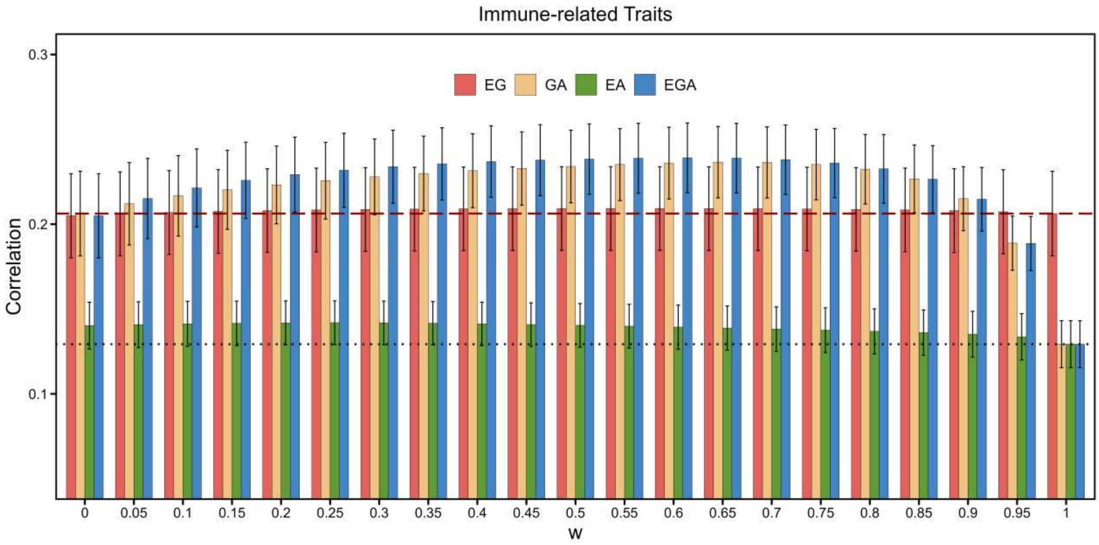

Figure S54. The Average Prediction Accuracy for Immune-related Traits

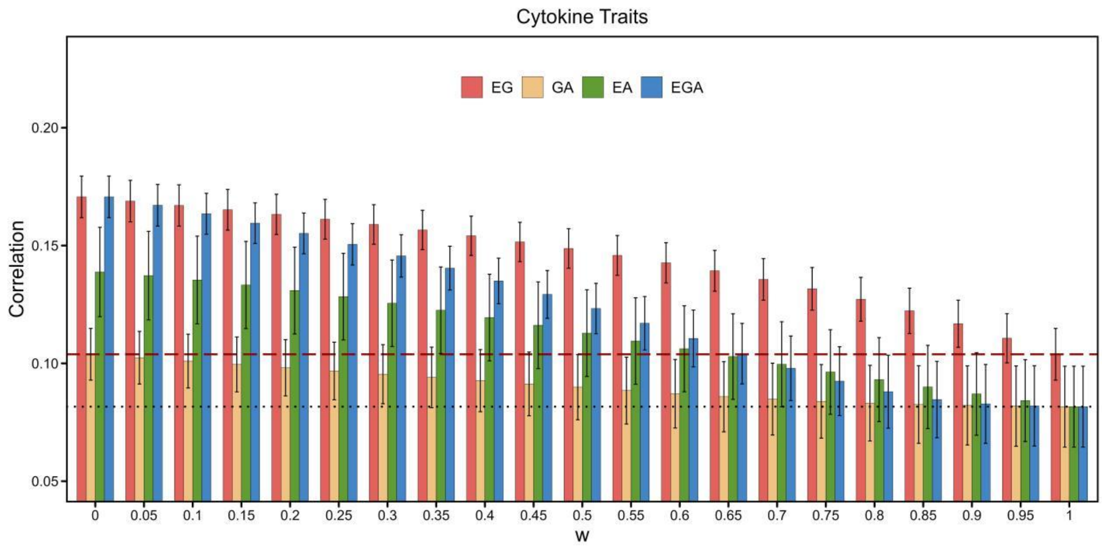

Figure S55. The Average Prediction Accuracy for Cytokine Traits

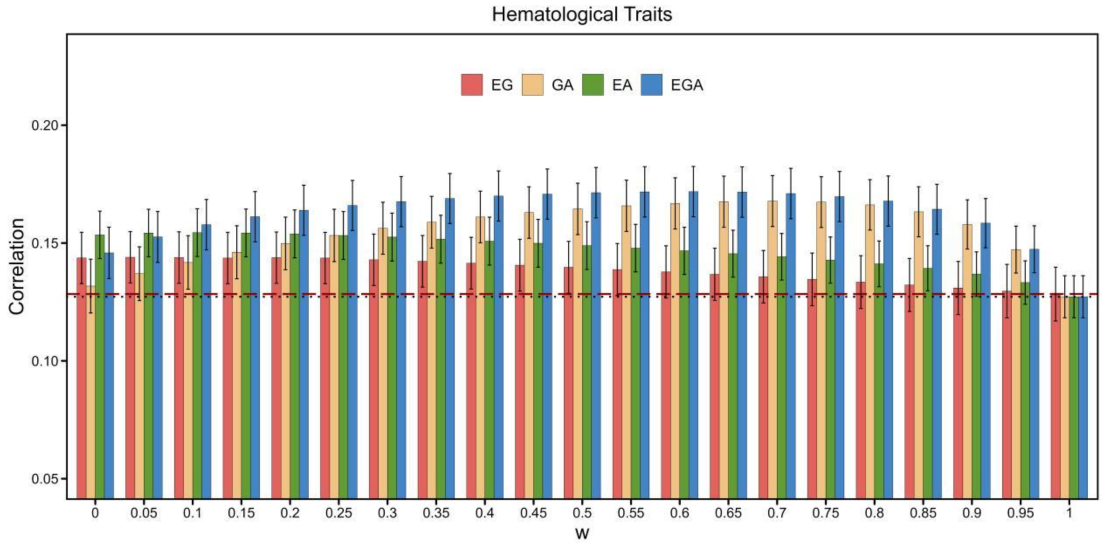

Figure S56. The Average Prediction Accuracy for Hematological Traits

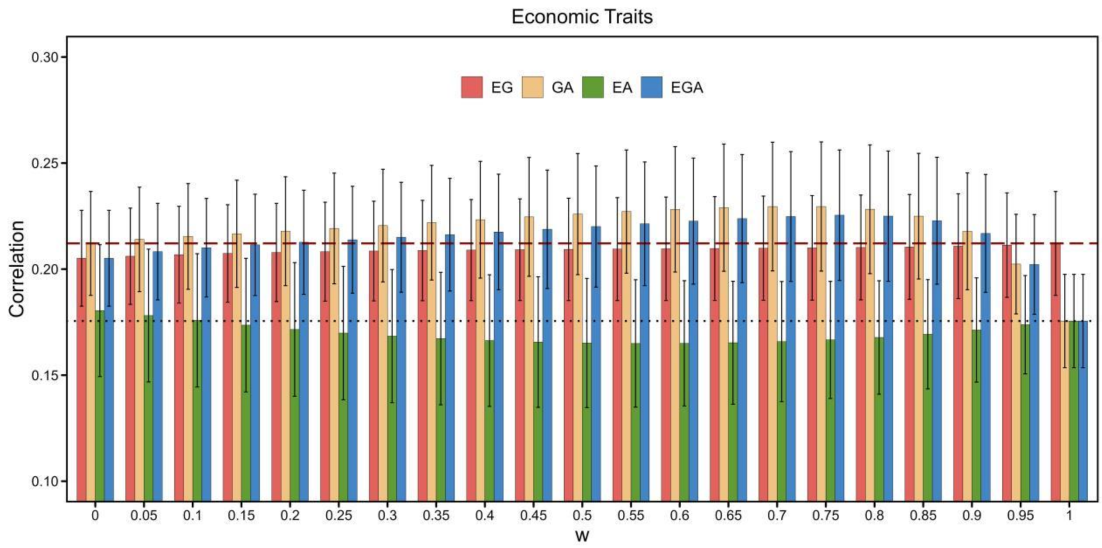

Figure S57. The Average Prediction Accuracy for Economic Traits
